# Diversely substituted sulfamides for fragment-based drug discovery of carbonic anhydrase inhibitors: synthesis and inhibitory profile

**DOI:** 10.1080/14756366.2022.2051023

**Published:** 2022-03-16

**Authors:** Tatiana Sharonova, Petr Zhmurov, Stanislav Kalinin, Alessio Nocentini, Andrea Angeli, Marta Ferraroni, Mikhail Korsakov, Claudiu T. Supuran, Mikhail Krasavin

**Affiliations:** a Institute of Chemistry, Saint Petersburg State University, Saint Petersburg, Russia; b Neurofarba Department, Universita Degli Studi di Firenze, Florence, Italy; c Pharmaceutical Technology Transfer Center, Ushinsky Yaroslavl State Pedagogical University, Yaroslavl, Russia

**Keywords:** Carbonic anhydrase, zinc-binding groups, sulfamides, co-operative fragment screening, solubility

## Abstract

A series of sulfamide fragments has been synthesised and investigated for human carbonic anhydrase inhibition. One of the fragments showing greater selectivity for cancer-related isoforms *h*CA IX and XII was co-crystalized with *h*CA II showing significant potential for fragment periphery evolution *via* fragment growth and linking. These opportunities will be identified in the future *via* the screening of this fragment structure for co-operative carbonic anhydrase binding with other structurally diverse fragments.

**Figure:**
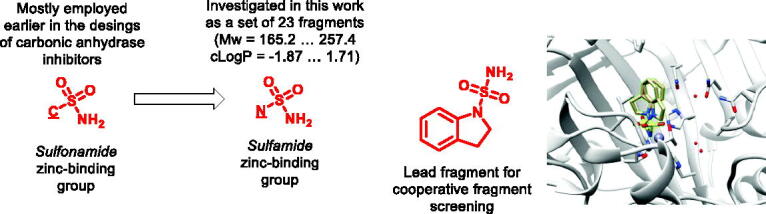

## 1. Introduction

The carbonic anhydrase (CA) family of Zn(II) metalloenzymes (EC 4.2.1.1) catalyses the reversible hydration of carbon dioxide to bicarbonate anion, a fundamental reaction that controls physiological processes requiring pH control as well as ion transport and fluid secretion^1^. Hyperactivity of specific CA isoforms^2^ in various disease states makes these enzymes potential (and sometimes already validated) targets for therapeutic intervention with small molecule carbonic anhydrase inhibitors (CAIs)^3^.

Clinically validated applications of CAIs currently include, among other diseases, the treatment of glaucoma^4^, idiopathic intracranial hypertension^5^, high-altitude sickness^6^, congestive heart failure^7^, peptic ulcers^8^ and epilepsy^9^. Another important potential application of CAIs (specifically, of related human (*h*) CA IX and XII isoforms^10^) is in neoplastic therapy^11^. The current state of development in this field is underscored by the *h*CA IX-selective drug SLC-0111^12^ which is undergoing phase 1 b clinical study for tumours overexpressing *h*CA IX^13^ and non-selective inhibitor E7070 (indisulam) developed by Eisai Co., Ltd. which successfully completed phase II clinical study^14^. The highly promising application of CAIs as antibacterials is based on the premise of selective inhibition of microbial CAs (crucial for the survival of bacteria) without affecting the CAs in the same concentration range^15^. Thus, CA inhibition from such microorganisms as *Vibrio cholerae*^16^*, Burkholderia pseudomallei*^17^*, Mycobacterium tuberculosis*^18^*, Salmonella enterica*^19^*, Helicobacter pylori*^20^*, Escherichia coli*^21^ and many others^15^.

**Figure 1.**
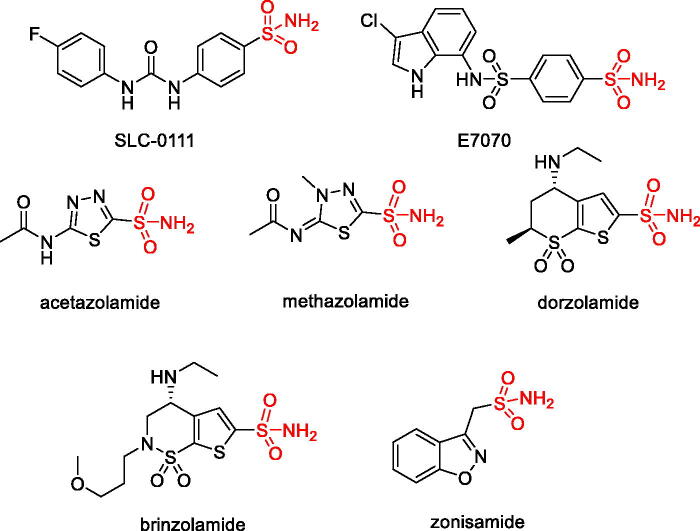
CAIs in clinical development and clinical use.

Considering the plethora of validated and potential therapeutic applications of CAIs, the discovery of new chemotypes endowed with CA inhibitory activity will continue to be a significant aim. CA contains a Zn^2+^ ion in its active site which mandates the zinc-binding nature of active site targeting pharmacophoric groups which can be employed in the design of new CAIs. Indeed, most of the clinically investigated (SLC-0111 and E7070) and used (e.g. acetazolamide, methazolamide, dorzolamide, brinzolamide and zonisamide) are primary sulphonamides (CSO_2_NH_2_) in which the sulphonamide group anchors to the prosthetic zinc ion and the molecular periphery defines the potency and isoform selectivity of these CAIs^2^ (Figure 1).

**Figure 2.**
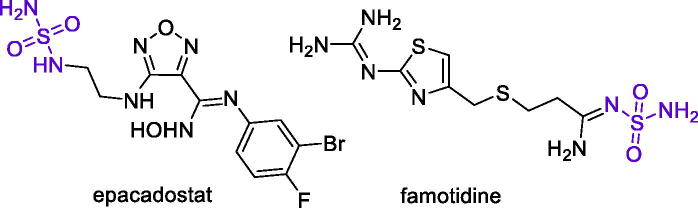
Examples of clinically used sulfamide drugs.

Many other zinc-binding motifs have been implicated as warheads in the CAI design^22^. Among them, sulfamides (NSO_2_NH_2_) appears as an attractive alternative to the frequently studied sulphonamides. Indeed, sulphonamides are expected to have greater polarity and solubility compared to sulphonamides, due to the presence of an additional nitrogen atom. Moreover, primary sulphonamide group is found in such drugs as anticancer epacadostat^23^ and gastric ulcer medication famotidine^24^, both of which (epacadostat^25^ and famotidine^26^) were also found to inhibit various CA isoforms (Figure 2).

The discovery of novel sulfamide CAIs would traditionally entail synthesis of structurally diverse libraries of compounds and their screening against an isoform panel of CAs. We have recently validated an approach^27^ to the discovery of new sulphonamide CAIs based on the simultaneous screening of a diverse set of chemical fragments (i.e. small, Mw <∼250 and polar, cLopP< 3.0^28^) along with sulphonamide zink-binding wasread (in particular, benzenesulfonamide or BSA). This led not only to the discovery of over 100 fragment hits which potentiated the binding of BSA but also to rediscovery of BSA-based CAIs with the molecular periphery replicating the fragment co-binders. In our intent aimed at the discovery of novel sulfamide CAIs, we decided to take a similar approach. Realisation of such an approach would require synthesis of a library of fragment-like sulfamides and profiling them against a panel of human CAs (in this case, anti-glaucoma target *h*CA II, two membrane-associated cancer-related targets *h*CA IX and XII and the usual cytosolic off-target *h*CA I). In this work, we aimed at the realisation of this approach and selection of a suitable sulfamide zinc-binding warhead for fragment-based discovery of novel sulfamide-type CAIs, a chemotype much less studied in the context of CA inhibition compared to sulphonamides^29^. Moreover, in this study, we were looking to identify: fragments that do not display apparently high intrinsic selectivity towards specific CA isoforms (mindful that such a selectivity will be gained in the future from co-operative screening of specific “tail” fragments^30^) and yet would show a tendency to inhibit cancer-related isoforms *h*CA IX and XII over cytosolic *h*CA I and II. Of particular interest would be fragments that do not display a pronounce potency against CA isoforms of interest, ideally in the 10^−7 ^M range of K_i_ values (so that the future contribution from co-operative fragment binding would be more pronounced). Specific emphasis was put on conformationally constrained fragments which would be structurally close to the classical BSA zinc-binding motif and would co-crystallize with any of the isoforms (e.g. the most readily available *h*CA II) to further guide further fragment evolution *via* growing, linking and merging^31^. Herein, we report on the successful realisation of this strategy.

## 2. Results and discussion

**Scheme 1.**
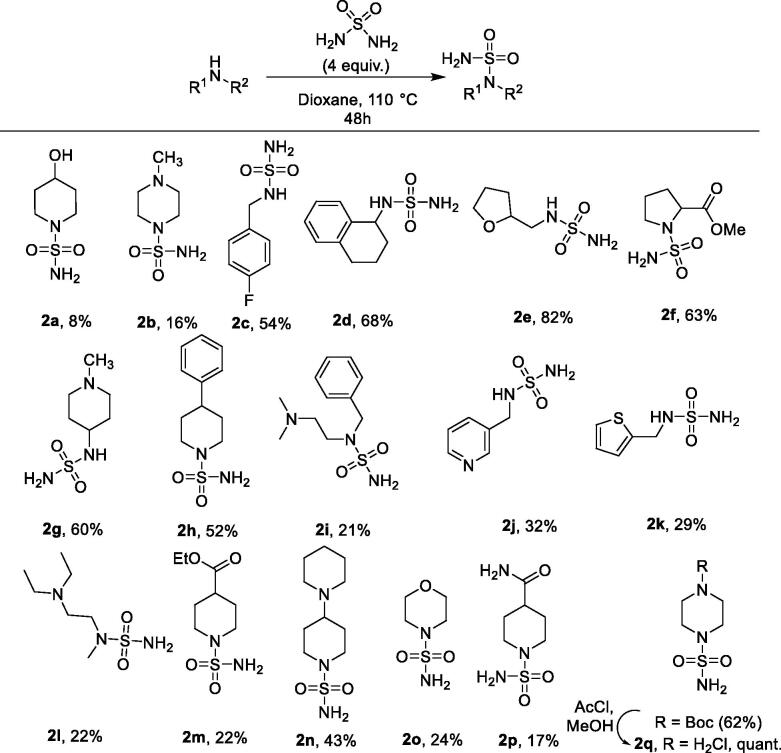
Synthesis of unsymmetrically substituted primary sulfamides **2a–q**.

Seventeen non-symmetrically substituted primary sulfamides were synthesised from inorganic sulfamide **1** *via* direct nucleophilic substitution at the sulphur atom, *via* the thermally promoted reaction in dioxane with a four-fold excess of **1**, conducted at 110 °C over 48 h^32^. The yields of the resulting compounds **2a–q** were generally modest to good (Scheme 1).

**Scheme 2.**
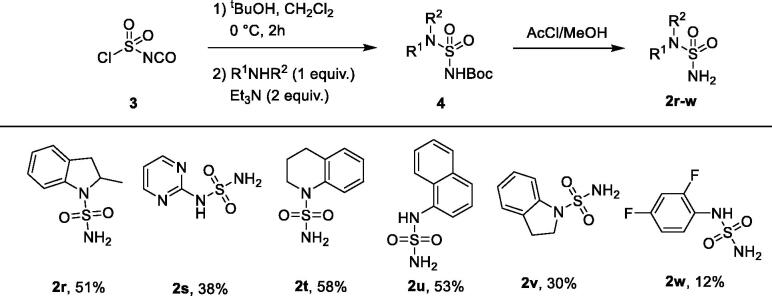
Synthesis of unsymmetrically (hetero)aromatic amine-substituted sulfamides **2r–w**.

The same thermally promoted protocol, not unexpectedly, did not work for less reactive (hetero)aromatic amines. Thus, an alternative approach was taken^33^. Instead of sulfamide, commercially available chlorosulfonyl isocyanate **3** dissolved in dichloromethane at 0 °C was reacted with 1 equiv of *tert*-butanol to give the Boc-protected amino-sulfonyl-chloride (**4**), which was subsequently added slowly to a solution of 1 equiv of the respective hetero(aromatic) amine in the presence of 2 equiv of triethylamine in dichloromethane at 0 °C. In this case, again, the yields of unsymmetrically substituted primary sulfamides **2r–w** were modest to good over two steps (Scheme 2).

From the physicochemical data summarised for fragments **2a–w** in Table 1, one can appreciate their being distinctly fragment-like (*M_w_* = 165.2 … 257.4, cLogP = −1.87 … 1.71). Furthermore, the inhibitory data reveal the absence of apparent isoform selectivity displayed by these fragments which is perfectly in line with the limited size of the molecular periphery (typically responsible for making additional contacts with the protein and ensuring higher potency and isoform selectivity). With our initial focus on the cancer-related, membrane-bound CA isoforms *h*CA IX and XII compounds displaying greater selectivity towards these isoforms against cytosolic *h*CA I and II (structural homologs of each other **2r** and **2v**) received our priority attention.

**Table 1.** Calculated physicochemical properties and *h*CA I, II, IX and XII inhibitory profile of compounds **2a–w**.

Compound	Mw	cLogP^*a*^	Ki (nM)^b^			
			*h*CA I	*h*CA II	*h*CA IX	*h*CA XII
**2a**	180.2	−1.87	75.6	42.7	54.2	25.3
**2b**	179.2	−1.01	64.2	32.1	58.6	52.5
**2c**	204.2	0.42	126.2	70.5	30.7	51.7
**2d**	226.3	1.22	120.7	42.6	36.8	59.7
**2e**	180.2	−0.77	38.4	63.4	92.7	98.0
**2f**	208.2	−0.65	52.1	69.8	78.2	83.6
**2g**	193.3	−0.72	89.7	52.1	63.2	38.4
**2h**	240.3	1.57	359.5	120.4	58.2	96.7
**2i**	257.4	0.53	28.4	39.8	65.1	19.7
**2j**	187.2	−0.98	98.5	90.2	29.5	16.5
**2k**	192.3	0.15	22.6	48.2	73.2	66.8
**2l**	209.3	−0.12	59.6	92.3	126.5	72.5
**2m**	236.3	0.11	62.8	42.8	58.1	25.9
**2n**	247.4	0.43	497.3	99.3	72.5	35.1
**2o**	166.2	−1.05	59.2	66.9	82.1	100.9
**2p**	207.3	−1.39	88.2	59.4	46.2	40.2
**2q**	165.2	−1.60	68.9	52.9	78.2	100.2
**2r**	212.3	1.12	867.9	315.7	94.7	116.2
**2s**	174.2	−1.27	29.6	59.4	68.0	39.3
**2t**	212.3	1.31	231.4	89.5	34.2	114.2
**2u**	222.3	1.71	160.1	56.7	25.4	72.3
**2v**	198.2	0.79	723.5	472.2	102.5	95.7
**2w**	208.2	0.81	45.7	76.3	38.5	224.0
Acetazolamide			250	125	25	5.7

^a^Caclculated using Molinspiration Chemoinformatics [34].

^b^Mean from three different assays, by a stopped flow technique (errors were in the range of ± 5–10% of the reported values).

**Figure 3.**
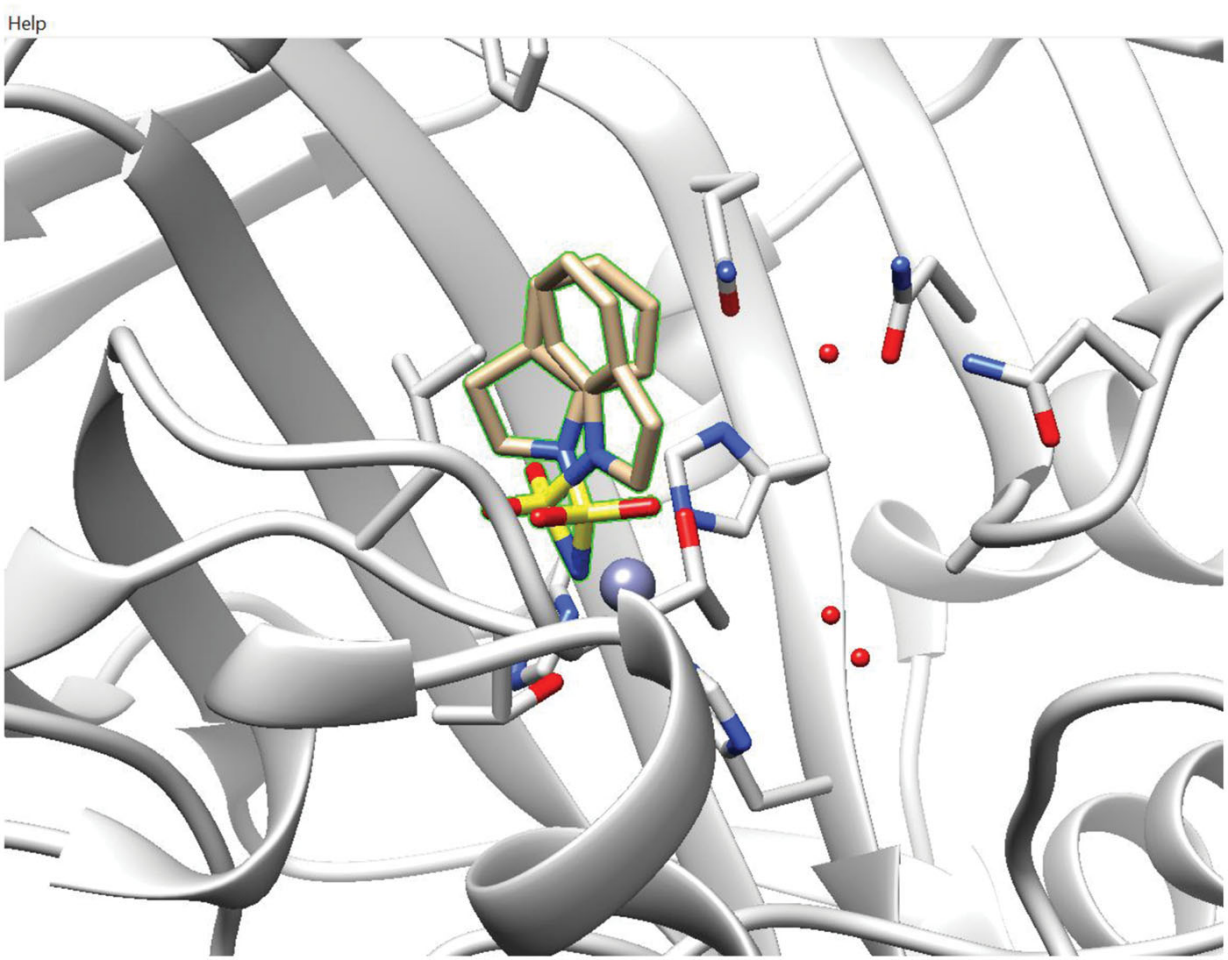
Co-crystal structure of fragment sulfamide **2v** with *h*CA II (PDB code 7QSI).

After much experimentation, compound **2v** was co-crystallized with recombinant *h*CA II and its structure was resolved (Figure 3).

As one can see from the crystal structure of **2v** with *h*CA II isoform, the small (Mw = 198.2) *N*-(aminosulfonyl)indoline fragment displayed two finding poses within the *h*CA II active site. In both poses, the sulfonylamino groups are anchored to the prosthetic zinc ion (displayed as a grey sphere). The validity of the two binding poses signifies the fact that the binding of **2v** leaves a significant room for the periphery growth around this fragment and makes it a highly suitable candidate for co-operative screening with other structurally diverse fragments in order to identify the starting points for the structural evolution of this fragment.

## 3. Materials and methods

### 3.1. Chemical synthesis – general

NMR spectra were recorded on a Bruker Avance III 400 spectrometer (^1^H: 400.13 MHz; ^13^С: 100.61 MHz; chemical shifts are reported as parts per million (*δ*, ppm); the residual solvent peaks were used as internal standards: δ 7.28 ^1^H in CDCl_3_, δ 77.02 ppm for ^13 ^C in CDCl_3_; multiplicities are abbreviated as follows: s = singlet, d = doublet, t = triplet, q = quartette, m = multiplet, br = broad; coupling constants, *J*, are reported in Hz. Mass spectra were recorded on a Bruker microTOF spectrometer (ESI ionization).

#### 3.1.1. General procedure for the synthesis of compounds 2a–q

A mixture of corresponding amine (2 mmol) and sulphuric diamide (8 mmol, 768 mg) in dry 1.4-dioxane (4 mL) was stirred at 110 ^o^C for 48 h. If amine was in the form of hydrochloride salt, an additional equivalent of triethylamine was added. CH_2_Cl_2_ (5 mL) was added and the resulting precipitate was filtered off, washed with ethyl acetate (5 mL). The filtrate and the washings were combined and solvent was evaporated under reduced pressure. The crude product was purified by column chromatography on silica gel using CH_2_Cl_2_:2-propanol 20:1–10:1 gradient as eluent (*R_f_* values are given for solvent system indicated).

#### 3.1.2. 4-Hydroxypiperidine-1-sulphonamide (2a)

*R_f_* = 0.30 (EtOAc); ^1^H NMR (400 MHz, DMSO-*d*_6_) *δ* = 6.66 (s, 2H), 4.69 (d, *J* = 3.9 Hz, 1H), 3.58 (dq, *J* = 7.8, 4.0 Hz, 1H), 3.22 (ddd, *J* = 11.0, 6.5, 3.8 Hz, 2H), 2.74 (ddd, *J* = 12.0, 9.0, 3.3 Hz, 2H), 1.77 (ddt, *J* = 13.7, 7.2, 3.6 Hz, 2H), 1.46 (dtd, *J* = 12.5, 8.6, 3.7 Hz, 2H) ppm; ^13 ^C NMR (101 MHz, DMSO-*d*_6_, Dept) *δ* = 65.049 (C-OH), 43.80 and 33.47 (2 CH_2_) ppm; HRMS (ESI) C_5_H_11_N_2_O_3_S^+^ *m/z*: [M – H]^−^ 179.0492 (calc 179.0485).

#### 3.1.3. 4-Methylpiperazine-1-sulphonamide (2 b)

*R_f_* = 0.61 (MeOH); ^1^H NMR (400 MHz, DMSO-*d*_6_) *δ* = 6.76 (s, 2H), 2.95 (t, *J* = 4.9 Hz, 4H), 2.38 (t, *J* = 5.0 Hz, 4H), 2.19 (s, 3H) ppm; ^13 ^C NMR (101 MHz, DMSO-*d*_6_, DEPT) *δ* = 54.08 (CH_2_), 46.14 (CH_2_), 45.89 (CH_3_) ppm; HRMS (ESI) C_5_H_12_N_3_O_2_S+ *m/z*: [M − H]^−^ 178.0644 (calc 178.0645).

#### 3.1.4. N*-(4-Fluorobenzyl)sulfamide (2c)*

^1^H NMR (400 MHz, DMSO-*d*_6_) *δ* = 7.39 (dd, *J* = 8.5, 5.7 Hz, 2H), 7.15 (t, *J* = 8.9 Hz, 2H), 7.07 (t, *J* = 6.5 Hz, 1H), 6.63 (s, 2H), 4.07 (d, *J* = 6.5 Hz, 2H) ppm; ^13 ^C NMR (101 MHz, DMSO-*d*_6_, DEPT) *δ* = 161.70 (d, *J* = 242.2 Hz), 135.39 (d, *J* = 2.9 Hz), 130.05 (d, *J* = 8.1 Hz, CH), 115.29 (d, *J* = 21.3 Hz, CH), 45.77 (s, CH_2_) ppm; ^19 ^F NMR (470 MHz, DMSO-*d*_6_) *δ* = −116.15 ppm; HRMS (ESI) C_7_H_9_FN_2_NaO_2_S^+^ *m/z*: [M + Na]^+^ 227.0272 (calc 227.0261).

#### 3.1.5. N*-(1,2,3,4-Tetrahydronaphthalen-1-yl)sulfamide (2d)*

*R_f_* = 0.80 (CH_2_Cl_2_-CH_3_OH 4:1); ^1^H NMR (400 MHz, DMSO-*d*_6_) *δ* = 7.57 − 7.50 (m, 1H), 7.19 − 7.11 (m, 2H), 7.08 − 7.02 (m, 1H), 6.96 (d, J = 8.9 Hz, 1H), 6.65 (s, 2H), 4.39 (td, *J* = 8.0, 5.2 Hz, 1H), 2.79 − 2.57 (m, 2H), 2.11 − 2.00 (m, 1H), 1.96 − 1.80 (m, 2H), 1.74 − 1.60 (m, 1H) ppm;

^13 ^C NMR (101 MHz, DMSO-*d*_6_, DEPT) *δ* = 138.15, 137.41, 129.43, 128.94, 127.07, 126.02, 51.32 (*C*H), 30.89, 29.23 and 20.39 (3*C*H_2_) ppm; HRMS (ESI) C_10_H_14_N_2_NaOS^+^ *m/z*: [M + Na]^+^ 249.0671 (calc 249.0668).

#### 3.1.6. N*-((tetrahydrofuran-2-yl)methyl)sulfamide (2e)*

*R_f_* = 0.59 (DCM:*i*-PrOH = 9:1); ^1^H NMR (400 MHz, CDCl_3_) *δ* = 4.94 (br. t, J = 6.0 Hz, 1H), 4.88 (br. s, 2H), 4.08 (ddt, *J* = 10.5, 7.3, 3.4 Hz, 1H), 3.88 (dt, *J* = 8.3, 6.7 Hz, 1H), 3.82 − 3.73 (m, 1H), 3.28 (ddd, *J* = 13.3, 6.4, 3.2 Hz, 1H), 3.14 (ddd, *J* = 13.4, 7.8, 5.8 Hz, 1H), 2.05 − 1.85 (m, 3H), 1.68 − 1.58 (m, 1H) ppm; ^13 ^C NMR (101 MHz, CDCl_3_, DEPT) *δ* = 77.65 (CH), 68.21, 47.52, 28.61 and 25.76 (4 CH_2_) ppm; HRMS (ESI) C_5_H_12_N_2_NaO_3_S+ *m/z*: [M + Na]^+^ 203.0464 (calc 203.0461).

#### 3.1.7. Methyl sulfamoylprolinate (2f)

*R_f_* = 0.75 (EtOAc); ^1^H NMR (400 MHz, DMSO-*d*_6_) = 6.82 (s, 2H), 4.18 (dd, *J* = 8.9, 4.1 Hz, 1H), 3.27 (dq, *J* = 6.2, 3.5, 2.9 Hz, 2H), 2.18 − 2.07 (m, 1H), 1.92 − 1.80 (m, 3H) ppm; ^13 ^C NMR (101 MHz, DMSO-*d*_6_, DEPT) *δ* = 173.24, 60.47 and 52.28 (CH and CH_3_), 49.11, 31.06 and 24.92 (3 CH_2_) ppm; HRMS (ESI) C_6_H_13_N_2_O_4_S^+^*m/z*: [M + H]^+^ 209.0599 (calc 209.0591).

#### 3.1.8. (1-Methylpiperidin-4-yl)sulfamide (2g)

*R_f_* = 0,13 (MeOH); ^1^H NMR (400 MHz, DMSO-*d*_6_) *δ* = 6.47 (s, 3H), 3.01 (tt, *J* = 10.6, 4.2 Hz, 1H), 2.68 (d, *J* = 11.9 Hz, 2H), 2.12 (s, 3H), 1.85 (qd, 11.9, 2.0 Hz, 4H), 1.42 (qd, *J* = 12.5, 4.2 Hz, 2H) ppm; ^13 ^C NMR (101 MHz, DMSO-*d*_6_) *δ* = 54.78, 50.36, 46.41, 32.86 ppm; HRMS (ESI) C_6_H_16_N_3_O_2_S+ *m/z*: [M + H]^+^ 194.0955 (calc 194.0958).

#### 3.1.9. 4-Phenylpiperidine-1-sulphonamide (2 h)

*R_f_* = 0.86 (EtOAc); ^1^H NMR (400 MHz, DMSO-*d*_6_) *δ* = 7.34 − 7.25 (m, 4H), 7.21 (td, *J* = 6.8, 1.7 Hz, 1H), 6.75 (br.s, 2H), 3.59 (br.d, *J* = 12.1 Hz, 2H), 2.63 (td, *J* = 12.2, 2.7 Hz, 2H), 2.59 − 2.53 (m, 1H), 1.86 (br.d, *J* = 12.2 Hz, 2H), 1.69 (qd, *J* = 12.5, 4.0 Hz, 2H) ppm;^13^C NMR (101 MHz, DMSO-*d*_6_, DEPT) *δ* = 145.97, 128.84 (*C*_Ar_H), 127.20 (*C*_Ar_H), 126.67 (*C*_Ar_H), 46.96 (*C*H_2_), 41.45 (*C*H), 32.45 (*C*H_2_) ppm; HRMS (ESI) C_11_H_16_N_2_NaO_2_S^+^ *m/z*: [M + Na]^+^ 263.0830 (calc 263.0825).

#### 3.1.10. Benzyl (2-(dimethylamino)ethyl)sulfamide (2i)

*R_f_* = 0.58 (MeOH); ^1^H NMR (400 MHz, DMSO-*d*_6_) *δ* = 7.40 − 7.33 (m, 4H), 7.31 − 7.26 (m, 1H), 6.91 (s, 2H), 4.26 (s, 2H), 3.09 (t, *J* = 6.8 Hz, 2H), 2.30 (t, *J* = 6.8 Hz, 2H), 2.07 (s, 6H) ppm; ^13 ^C NMR (101 MHz, DMSO-*d*_6_, DEPT) *δ* = 138.06, 128.76, 128.56 and 127.74 (3 C_Ar_H), 57.05, 51.41 and 45.72 (3 CH_2_), 45.52 (CH_3_) ppm; HRMS (ESI) C_11_H_20_N_3_O_2_S^+^ *m/z*: [M + H]^+^ 258.1276 (calc 258.1271).

#### 3.1.11. (Pyridin-3-ylmethyl)sulfamide (2j)

*R_f_* = 0.90 (MeOH); ^1^H NMR (400 MHz, CD_3_CN) *δ* = 8.55 (d, *J* = 2.2 Hz, 1H), 8.48 (dd, *J* = 4.8, 1.6 Hz, 1H), 7.76 (dt, *J* = 7.9, 1.9 Hz, 1H), 7.33 (dd, *J* = 7.8, 4.8 Hz, 1H), 5.62 (br. s, 1H), 5.31 (br. s, 2H), 4.20 (d, *J* = 6.3 Hz, 2H) ppm; ^13 ^C NMR (101 MHz, CD_3_CN) *δ* = 149.23, 148.59, 135.72, 133.71, 123.46, 44.32 ppm; HRMS (ESI) C_6_H_9_N_3_NaO_2_S^+^ *m/z*: [M + Na]^+^ 210.0308 (calc 210.0308).

#### 3.1.12. (Thiophen-2-ylmethyl) sulfamide (2k)

*R_f_* = 0.49 (DCM:*i*-PrOH = 20:1); ^1^H NMR (400 MHz, Acetone-*d_6_*) = 7.37 (dd, *J* = 5.2, 1.2 Hz, 1H), 7.07 (dq, *J* = 3.3, 1.1 Hz, 1H), 6.97 (dd, *J* = 5.1, 3.5 Hz, 1H), 6.20 (s, 1H), 5.99 (s, 2H), 4.45 (dd, *J* = 6.4, 1.0 Hz, 2H) ppm; ^13 ^C NMR (101 MHz, Acetone-*d_6_*, DEPT) *δ* = 141.21 (>C=), 126.62, 125.80, and 125.08 (3 C_Ar_H), 41.97 (CH_2_) ppm; HRMS (ESI) C_5_H_8_N_2_NaO_2_S_2_^+^ *m/z*: [M + Na]^+^ 214.9919 and 216.9877 (calc 214.9919 and 216.9878).

#### 3.1.13. (2-(Diethylamino)ethyl)(methyl)sulfamide (2 l)

*R_f_* = 0.58 (MeOH); ^1^H NMR (400 MHz, DMSO-*d*_6_) = 6.73 (s, 2H), 3.05 (t, *J* = 6.8 Hz, 2H), 2.68 (s, 2H), 2.55 (t, *J* = 6.8 Hz, 2H), 2.49 (q, *J* = 7.1 Hz, 4H), 0.96 (t, *J* = 7.1 Hz, 6H) ppm; ^13 ^C NMR (101 MHz, DMSO-*d*_6_, DEPT) *δ* = 50.53, 48.57 and 46.95(3 CH_2_), 35.57 and 12.01 (2 CH_3_) ppm; HRMS (ESI) C_7_H_20_N_3_O_2_S^+^ *m/z*: [M + H]^+^ 210.1274 (calc 210.1271).

#### 3.1.14. Ethyl 1-sulfamoylpiperidine-4-carboxylate (2 m)

*R_f_* = 0.81 (EtOAc); ^1^H NMR (400 MHz, DMSO-*d*_6_) *δ* = 6.71 (s, 2H), 4.07 (q, *J* = 7.1 Hz, 2H), 3.36 (dt, *J* = 12.3, 3.8 Hz, 2H), 2.62 (td, *J* = 11.6, 2.8 Hz, 2H), 2.42 (tt, *J* = 10.7, 4.0 Hz, 1H), 1.90 (br. dd, *J* = 13.5, 3.8 Hz, 2H), 1.60 (qd, *J* = 11.1, 3.8 Hz, 2H), 1.18 (t, *J* = 7.1 Hz, 3H) ppm; ^13 ^C NMR (101 MHz, DMSO-*d*_6_) *δ* = 174.25, 60.44, 45.55, 27.47, 14.54 ppm; HRMS (ESI) C_8_H_16_N_2_NaO_4_S^+^ *m/z*: [M + Na]^+^ 259.0722 (calc 259.0723).

#### 3.1.15. [1,4'-Bipiperidine]-1'-sulphonamide (2n)

*R_f_* = 0.24 (MeOH); ^1^H NMR (400 MHz, DMSO-*d*_6_) *δ* = 6.68 (br.s, 2H), 3.48 (br.d, J = 12.4 Hz, 2H), 2.53 − 2.41 (m, 3H), 2.44 (t, J = 5.1 Hz, 3H), 2.24 (tt, J = 11.3, 3.6 Hz, 1H), 1.76 (br.d, J = 11.7 Hz, 2H), 1.52 − 1.43 (m, 6H), 1.38 (q, J = 5.9 Hz, 2H) ppm; ^13 ^C NMR (101 MHz, DMSO-*d*_6_, DEPT) *δ* = 61.56, 50.16, 46.22, 27.21, 26.52, 25.01; HRMS (ESI) C_10_H_22_N_3_O_2_S^+^ *m/z*: [M + H]^+^ 248.1431 (calc 248.1428).

#### 3.1.16. Morpholine-4-sulphonamide (2o)

*R_f_* = 0.56 (EtOAc); ^1^H NMR (400 MHz, DMSO-*d*_6_) *δ* = 6.82 (s, 2H), 3.86 − 3.50 (m, 4H), 3.02 − 2.77 (m, 4H) ppm; ^13 ^C NMR (101 MHz, DMSO-*d*_6_) *δ* = 65.73, 46.43 ppm; HRMS (ESI) C_4_H_9_N_2_O_3_S^+^ *m/z*: [M − H]^−^ 165.0326 (calc 165.0339)

#### 3.1.17. 1-Sulfamoylpiperidine-4-carboxamide (2p)

*R_f_* = 0.61 (EtOAc:MeOH 3:1); ^1^H NMR (400 MHz, DMSO-*d*_6_) *δ* = 7.27 (s, 1H), 6.82 (s, 1H), 6.79 − 6.44 (m, 2H), 3.52 − 3.39 (m, 2H), 2.21 − 2.05 (m, 1H), 1.78 (d, *J* = 13.1 Hz, 2H), 1.57 (t, *J* = 12.7 Hz, 2H) ppm; ^13 ^C NMR (101 MHz, DMSO-*d*_6_) *δ* = 176.30, 45.96, 41.12, 28.07 ppm; HRMS (ESI) C_6_H_13_N_3_NaO_3_S^+^ *m/z*: [M + Na]^+^ 230.0565 (calc 230.0570)

#### 3.1.18. Piperazine-1-sulphonamide hydrochloride (2q)

^1^H NMR (400 MHz, DMSO-*d*_6_) *δ* = 9.60 (s, 2H), 7.08 (s, 2H), 3.21 (dd, *J* = 7.1, 3.6 Hz, 4H), 3.14 (dd, *J* = 7.0, 3.7 Hz, 4H) ppm; ^13 ^C NMR (101 MHz, DMSO-*d*_6_) *δ* = 43.27, 42.37 ppm; HRMS (ESI) C_4_H_12_N_3_O_2_S^+^ *m/z*: [M + H]^−^ 166.0649 (calc 166.0645).

#### 3.1.19. 2-Methylindoline-1-sulphonamide (2r)

*R_f_* = 0.81 (n-Hexane:EtOAc 1:1); ^1^H NMR (400 MHz, CDCl_3_) = 7.40 (d, *J* = 8.2 Hz, 1H), 7.19 (dt, *J* = 7.7, 3.7 Hz, 2H), 7.04 (t, *J* = 7.4 Hz, 1H), 4.46 (s br., 1H), 4.07 (s br., 2H), 3.52 − 3.43 (m, 2H), 2.67 (dd, *J* = 15.9, 3.5 Hz, 1H), 1.43 (d, *J* = 6.1 Hz, 3H) ppm; ^13 ^C NMR (101 MHz, CDCl_3_) *δ* = 141.32, 131.15, 127.79, 125.40, 124.10, 115.77, 59.19, 36.64, 22.63 ppm; HRMS (ESI) C_9_H_12_N_2_NaO_2_S^+^ *m/z*: [M + Na]^+^ 235.0515 (calc 235.0512).

#### 3.1.20. N*-(Pyrimidin-2-yl)sulfamide (2s)*

^1^H NMR (400 MHz, DMSO-*d*_6_) = 8.56 (d, *J* = 4.8 Hz, 4H), 7.06 (t, *J* = 4.9 Hz, 2H) ppm; ^13 ^C NMR (101 MHz, DMSO-*d*_6_) *δ* = 158.73, 158.26, 115.28 ppm; HRMS (ESI) C_4_H_6_N_4_NaO_2_S^+^ *m/z*: [M + Na]^+^ 197.1668 (calc 197.1672)

#### 3.1.21. 3,4-Dihydroquinoline-1(2H)-sulphonamide (2t)

*R_f_* = 0.55 (*n*-Hexane:EtOAc 3:1); ^1^H NMR (400 MHz, CDCl_3_) *δ* = 7.67 (d, *J* = 8.2 Hz, 1H), 7.17 (t, *J* = 7.6 Hz, 1H), 7.14 − 7.02 (m, 2H), 4.56 (s, 2H), 3.87 − 3.53 (m, 2H), 2.86 (t, *J* = 6.6 Hz, 2H), 2.06 (p, *J* = 6.5 Hz, 2H) ppm; ^13 ^C NMR (400 MHz, CDCl_3_, DEPT) *δ* = 137.20, 129.92, 129.53, 126.60, 124.65 and 123.61 (4 C_Ar_H), 47.28, 26.94 and 21.87 (3 CH_2_) ppm; HRMS (ESI) C_9_H_12_N_2_NaO_2_S^+^ *m/z*: [M + Na]^+^ 235.05110 (calc 235.0511)

#### 3.1.22. N*-(Naphthalen-1-yl)sulfamide (2 u)*

*R_f_* = 0.44 (*n*-Hexane:EtOAc 1:1); ^1^H NMR (400 MHz, DMSO-*d*_6_) *δ* = 9.27 (s, 1H), 8.35 − 8.25 (m, 1H), 7.96 − 7.87 (m, 1H), 7.75 (d, *J* = 8.1 Hz, 1H), 7.59 (dd, *J* = 7.6, 1.2 Hz, 1H), 7.55 − 7.47 (m, 3H), 7.00 (s, 2H) ppm; ^13 ^C NMR (101 MHz, DMSO-*d*_6_, Dept) *δ* = 134.74, 134.36, 129.17, 128.26, 126.43, 126.10, 125.49, 124.10, 121.57 ppm; HRMS (ESI) C_10_H_10_N_2_NaO2S^+^ *m/z*: [M + Na]^+^ 245.0359 (calc 245.0356)

#### 3.1.23. Indoline-1-sulphonamide (2v)

*R_f_* = 0.77 (EtOAc); ^1^H NMR (400 MHz, DMSO-*d*_6_) = 7.28 (d, *J* = 8.0 Hz, 1H), 7.25 − 7.20 (m, 1H), 7.23 (s, 2H), 7.16 (t, *J* = 7.7 Hz, 1H), 6.96 (t, *J* = 7.4 Hz, 1H), 3.80 (t, *J* = 8.4 Hz, 2H), 3.05 (t, *J* = 8.4 Hz, 2H) ppm; ^13 ^C NMR (101 MHz, DMSO-*d*_6_, DEPT) *δ* = 143.51 and 131.99 (2 >C_Ar_=), 127.63, 125.40, 122.83 and 114.21 (4 C_Ar_H), 50.39 (*C*H_2_), 27.76 (*C*H_2_) ppm; HRMS (ESI) C_8_H_10_N_2_NaO_2_S+ *m/z*: [M + Na]^+^ 221.0357 (calc 221.0355)

#### 3.1.24. N*-(2,4-Difluorophenyl)sulfamide (2w)*

^1^H NMR (400 MHz, CDCl_3_) *δ* = 7.40 (d, *J* = 8.2 Hz, 1H), 7.19 (dt, *J* = 7.7, 3.7 Hz, 2H), 7.04 (t, *J* = 7.4 Hz, 1H), 4.46 (s br., 1H), 4.07 (s br., 2H), 3.52 − 3.43 (m, 2H), 2.67 (dd, *J* = 15.9, 3.5 Hz, 1H), 1.43 (d, *J* = 6.1 Hz, 3H) ppm; ^13 ^C NMR (101 MHz, CDCl_3_) *δ* = 160.29 (dd, J = 248.7, 11.3 Hz), 154.88 (dd, J = 247.7, 12.1 Hz), 125.60 (dd, *J* = 9.6, 1.4 Hz, C_Ar_H), 121.02 (dd, *J* = 12.5, 3.7 Hz), 111.97 (dd, *J* = 22.3, 3.8 Hz), 104.40 (dd, *J* = 26.7, 23.7 Hz) ppm; ^19 ^F NMR (376 MHz, CDCl_3_) *δ* = −111.98, −123.53 ppm; HRMS (ESI) C_6_H_6_F_2_N_2_NaO_2_S+ *m/z*: [M + Na]^+^ 231.0010 (calc 231.0011)

### 3.2. Carbonic anhydrase inhibition testing

An Applied Photophysics stopped-flow instrument has been used for assaying the CA catalysed CO_2_ hydration activity^35^. Phenol red (at a concentration of 0.2 mM) has been used as indicator, working at the absorbance maximum of 557 nm, with 20 mM Hepes (pH 7.5) as buffer, and 20 mM Na_2_SO_4_ (for maintaining constant the ionic strength), following the initial rates of the CA-catalysed CO_2_ hydration reaction for a period of 10–100 s. The CO_2_ concentrations ranged from 1.7 to 17 mM for the determination of the kinetic parameters and inhibition constants. For each inhibitor at least six traces of the initial 5–10% of the reaction have been used for determining the initial velocity. The uncatalyzed rates were determined in the same manner and subtracted from the total observed rates. Stock solutions of inhibitor (0.1 mM) were prepared in distilled deionised water and dilutions up to 0.01 nM were done thereafter with the assay buffer. Inhibitor and enzyme solutions were preincubated together for 15 min at room temperature prior to assay, in order to allow for the formation of the EI complex. The inhibition constants were subsequently obtained by nonlinear least-squares methods using PRISM 3 and the ChengPrusoff equation, as reported earlier, and represent the mean from at least three different determinations. All CA isoforms were recombinant ones obtained in-house as reported earlier^36–39^.

### 3.3. Co-crystallization and X-ray data collection

Crystals of *h*CA II complexed with compound **2v** were obtained using the sitting drop vapour diffusion method. An equal volume of 0.8 mM solution of *h*CA II in Tris pH = 8.0 and 1.6 mM of the inhibitors in Hepes 20 mM pH = 7.4 was mixed and incubated for 15 min. 2 mL of the complex solution were mixed with 2 mL of a solution of 1.6 M sodium citrate, 50 mM Tris pH 8.0 and were equilibrated against the same solution at 296 K. Crystals of the complex grew in a few days. The crystals were flash-frozen at 100 K using a solution obtained by adding 25% (v/v) glycerol to the mother liquor solution as cryoprotectant. A data set on a crystal of the complex with the inhibitor **2v** was collected at the Centro di Cristallografia Strutturale (CRIST) in Florence using an Oxford Diffraction instrument equipped with a sealed tube Enhance Ultra (Cu) and a Onyx CCD detector. Data were integrated and scaled using the program XDS.24 Data processing statistics are showed in Table 2.

**Table 2.** Summary of data collection and atomic model refinement statistics for *h*CAII.^a^

	*h*CAII + **2v**
PDB ID	7QSI
Wavelength (Å)	0.999900
Space Group	P21
Unit cell (a, b, c, α, β, γ) (Å,°)	42.363, 41.557, 72.068 90.000, 104.495, 90.000
Limiting resolution (Å)	41.02–1.30 (1.34–1.30)
Unique reflections	52,866 (2056)
Rmerge (%)	6.4 (73.2)
Rmeas (%)	7.0 (84.4)
Redundancy	6.11 (3.98)
Completeness overall (%)	88.9 (46.7)
<I/σ(I)>	19.54 (2.1)
CC (1/2)	99.9 (63.8)
Refinement statistics	
Resolution range (Å)	41.02–1.30
Rfactor (%)	16.27
Rfree(%)	18.45
r.m.s.d. bonds(Å)	0.0135
r.m.s.d. angles (°)	1.8542
Ramachandran statistics (%)	
Most favoured	96.9
additionally allowed	3.1
outlier regions	0.0
Average B factor (Å^2^)	
All atoms	15.116
inhibitors	11.348
Solvent	24.123

^a^Values in parentheses are for the highest resolution shell.

### 3.4. Structure determination

The crystal structure of *h*CA II (PDB accession code: 7QSI) without solvent molecules and other heteroatoms was used to obtain initial phases of the structures using Refmac5^40^. 5% of the unique reflections were selected randomly and excluded from the refinement data set for the purpose of Rfree calculations. The initial |Fo – Fc| difference electron density maps unambiguously showed the inhibitor molecules. An electron density, which could be interpreted as a second molecule of inhibitor **2v**, was present near the *N*-terminus of the protein. Thus, a second **2v** molecule was introduced in the model with 0.75 occupancy. Atomic model for the inhibitor was calculated and energy minimised using the program JLigand 1.0.39. Refinements proceeded using normal protocols of positional, isotropic atomic displacement parameters alternating with manual building of the models using COOT^41^. Solvent molecules were introduced automatically using the program ARP^42^. The quality of the final model was assessed with COOT and Rampage^43^. Crystal parameters and refinement data are summarised in Table 2. Atomic coordinates were deposited in the Protein Data Bank (PDB accession code: 7QSI). Graphical representations were generated with Chimaera^44^.

## 4. Conclusions

We have described the synthesis and testing against a panel of human carbonic anhydrases (*h*CA I, II, IX and XII) of a series of hydrophilic, fragment sulfamides intended for fragment-based drug discovery of isoform-selective carbonic anhydrase inhibitors via cooperative screening with other, non-zinc-binding fragments. As expected from the minimal-periphery zinc-binding moieties, these fragment sulfamides demonstrated little selectivity across the panel of *h*CAs. However, for one of the fragment inhibitors (**2v**) which showed higher selectivity towards the cancer-related *h*CA isoforms (IX and CII), we obtained a crystal structure with the most abundant cytosolic isoform *h*CA II which showed two possible binding modes and thus significant room for cooperative fragment biding and subsequent periphery evolution.

## Supplementary Material

Supplemental Material

## Supplementary materials

Copies of ^1^H and ^13 ^C NMR spectra of compounds **2a–w**.
