# Supplementary material for: Diversely substituted sulfamides for fragment-based drug discovery of carbonic anhydrase inhibitors: synthesis and inhibitory profile
Source: J Enzyme Inhib Med Chem. 2022 Mar 16;37(1):857–65. doi: 10.1080/14756366.2022.2051023 (PMC8933014; doi:10.1080/14756366.2022.2051023)
Supplement: Supplemental Material [file IENZ_A_2051023_SM2031.pdf]

Supplementary materials for

Diversely Substituted Sulfamides for Fragment-Based Drug Discovery of Carbonic Anhydrase Inhibitors: Synthesis and Inhibitory Profile

Tatiana Sharonova, Petr Zhmurov, Stanislav Kalinin, Alessio Nocentini, Andrea Angeli, Marta Ferraroni, Mikhail Korsakov, Claudiu T. Supuran * and Mikhail Krasavin *

Contents:

Copies of ^{1}H and ^{13}C spectra for compounds **2a-w**

^{1}H NMR and ^{13}C (DEPT) spectra of compound **2a**

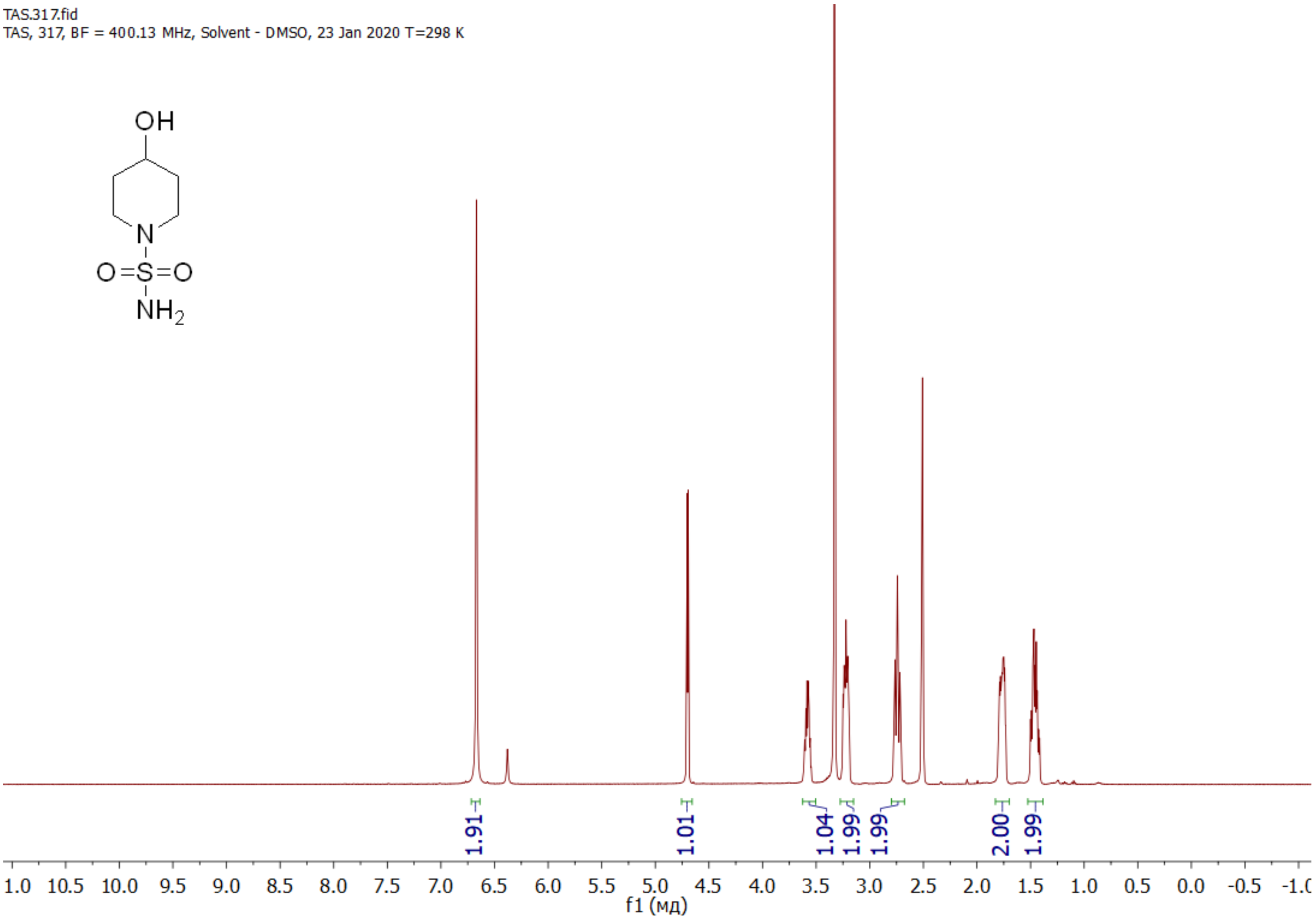

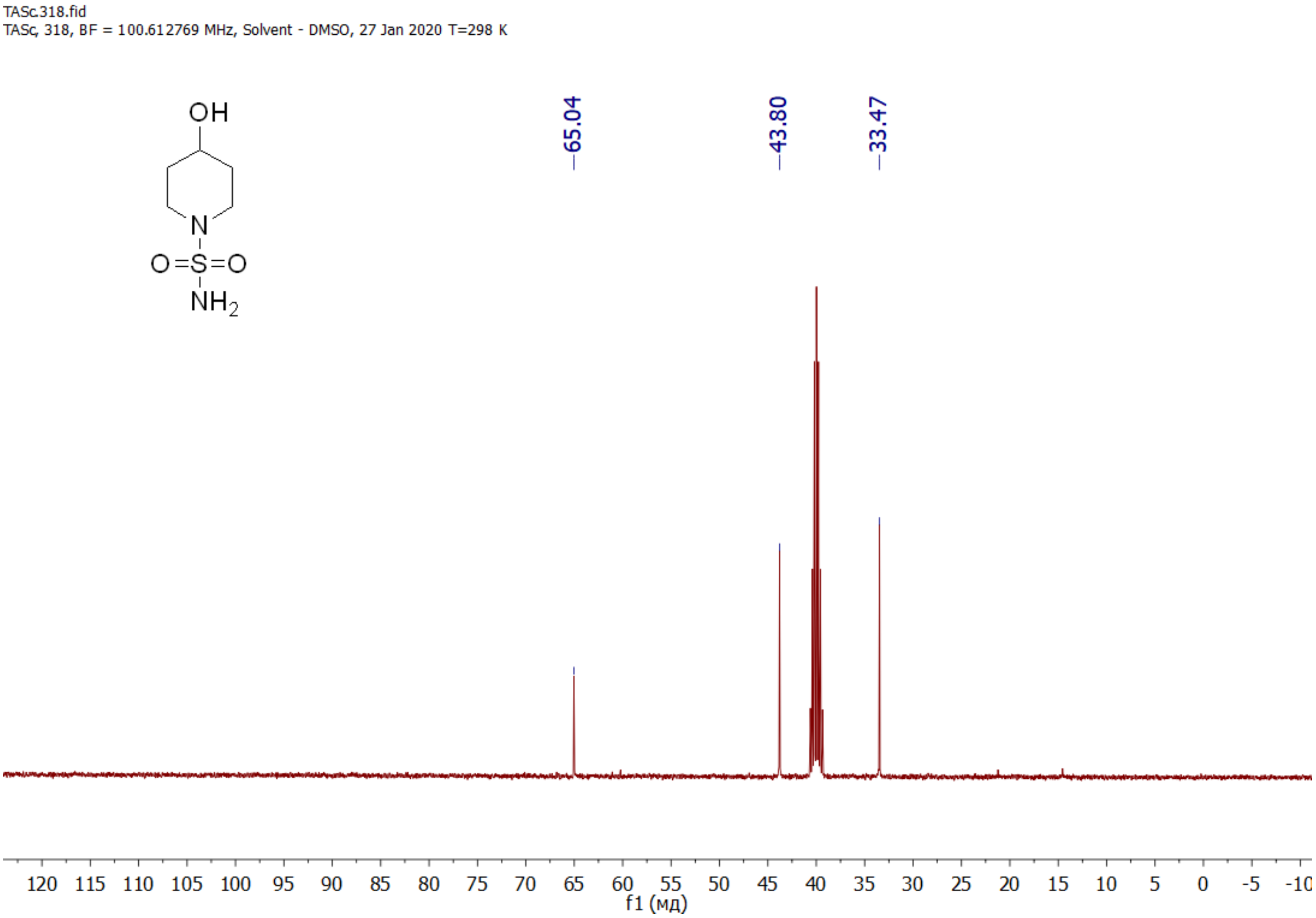

TASd.318.fid
TASd, 318, BF = 100.612769 MHz, Solvent - DMSO, 27 Jan 2020 T=298 K

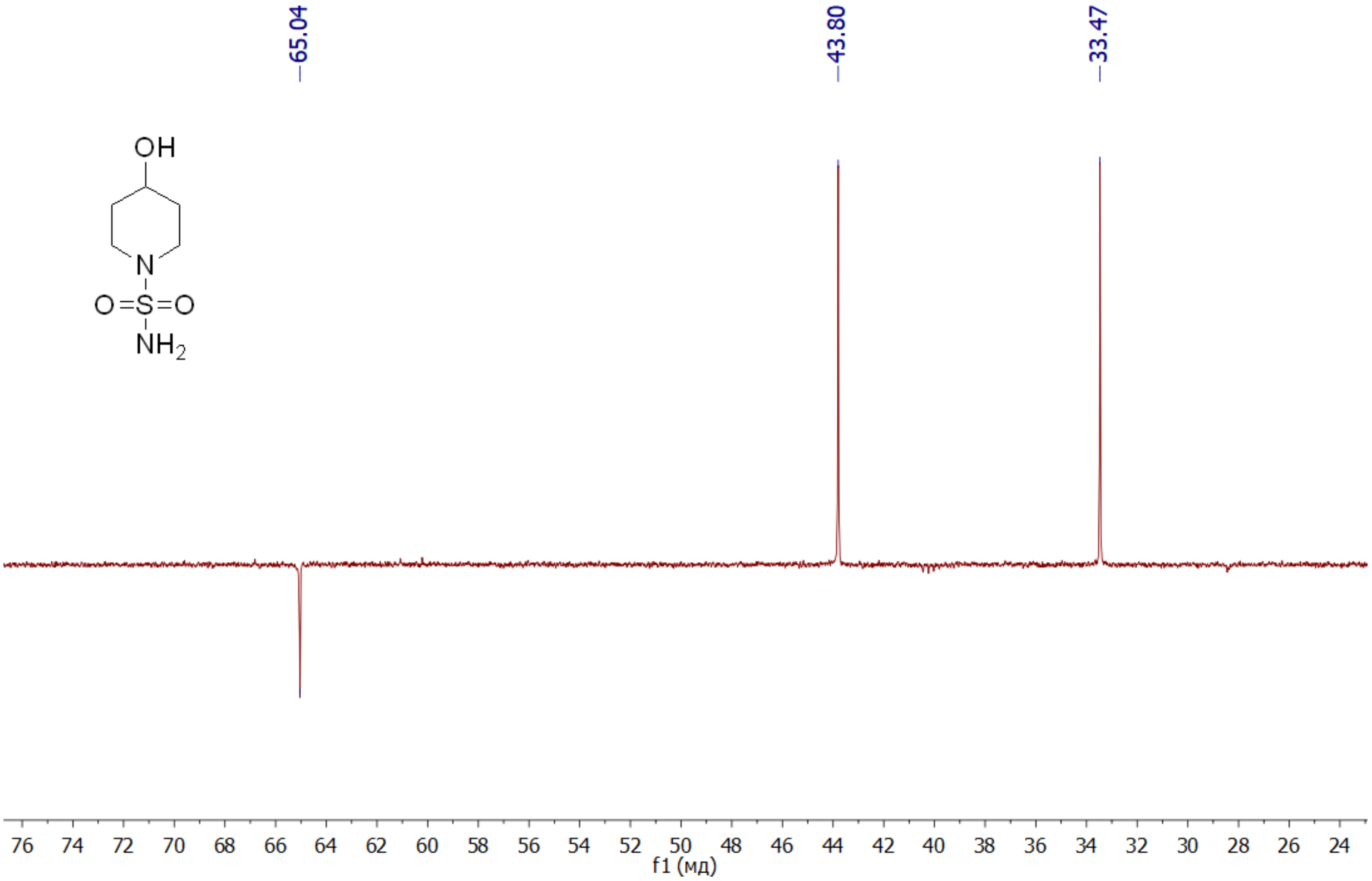

^{1}H NMR and ^{13}C (DEPT) spectra of compound **2b**

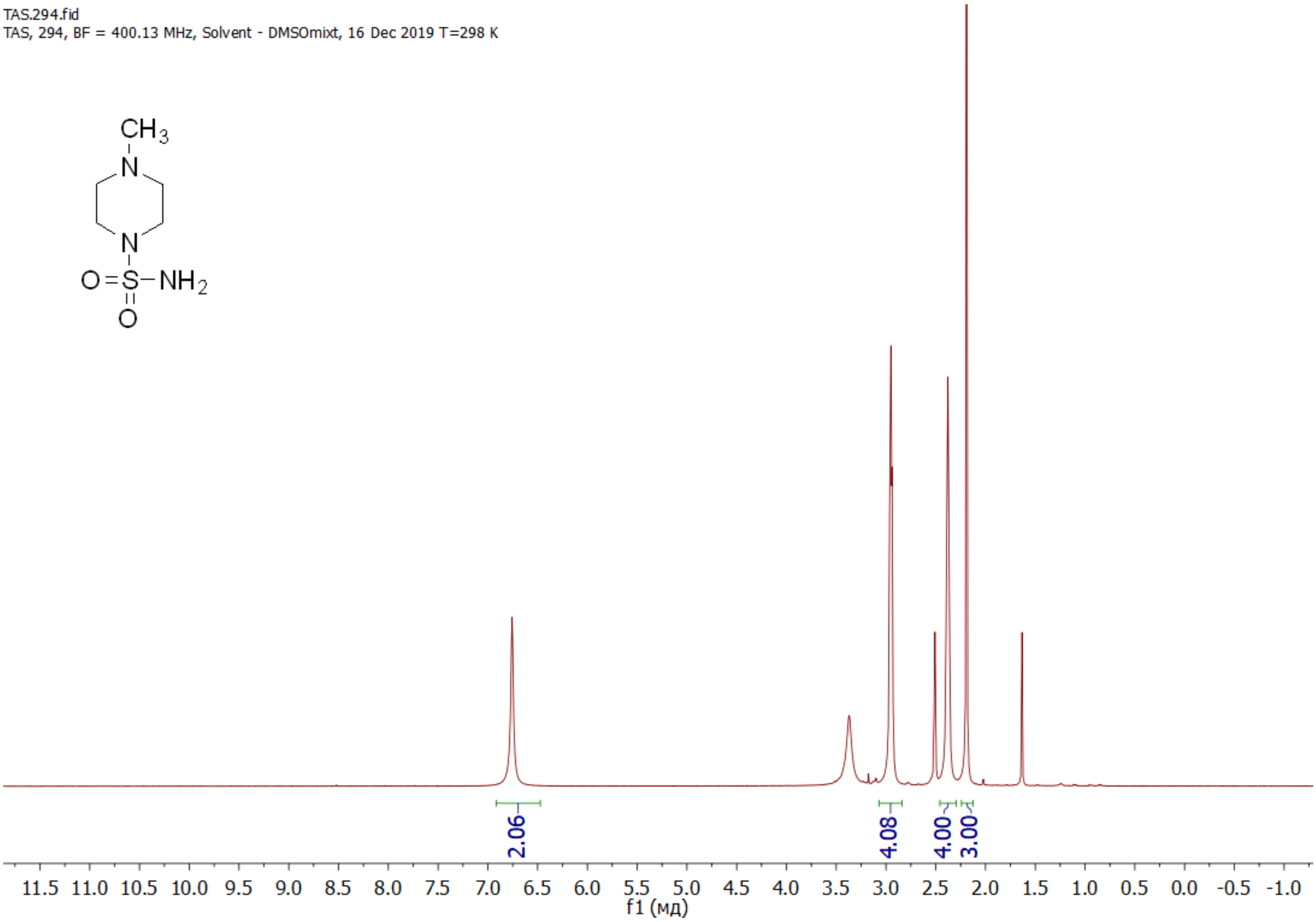

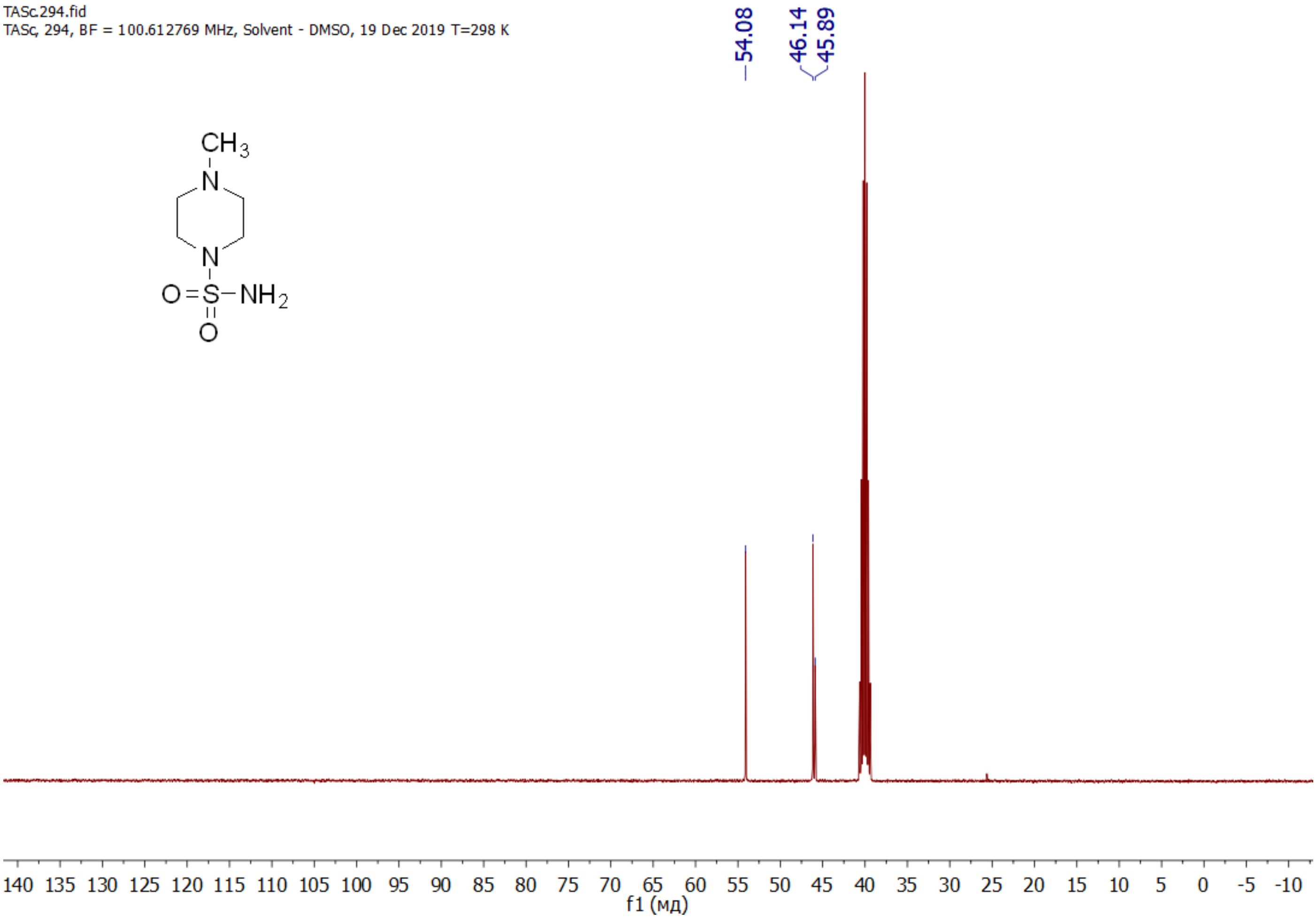

TASd.294.fid
TASd, 294, BF = 100.612769 MHz, Solvent - DMSO, 20 Jan 2020 T=298 K

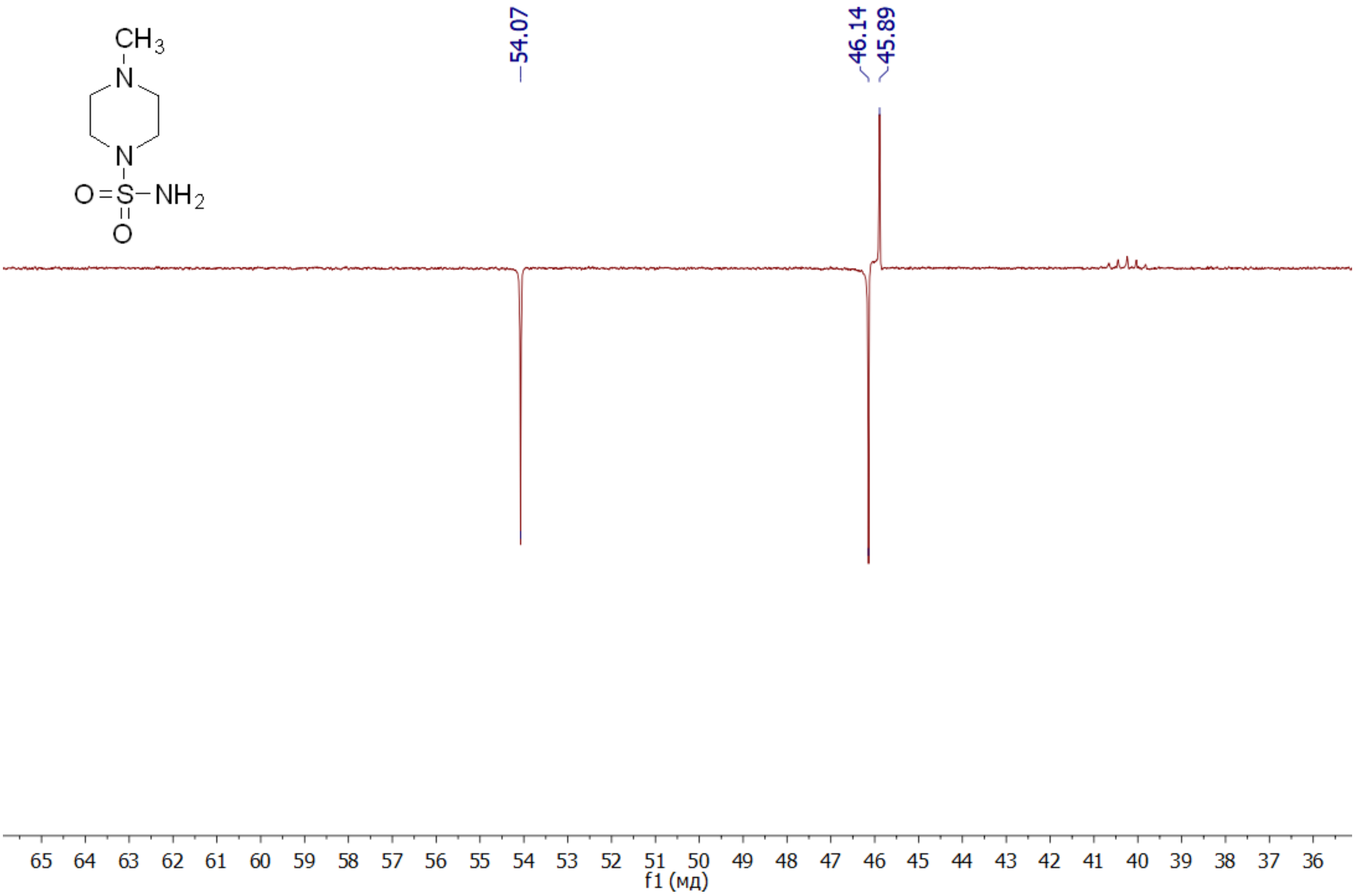

^{1}H NMR, ^{13}C (DEPT) and ^{19}F spectra of compound **2c**

TAS.334.fid
TAS, 334, BF = 400.13 MHz, Solvent - DMSO, 07 Feb 2020 T=298 K

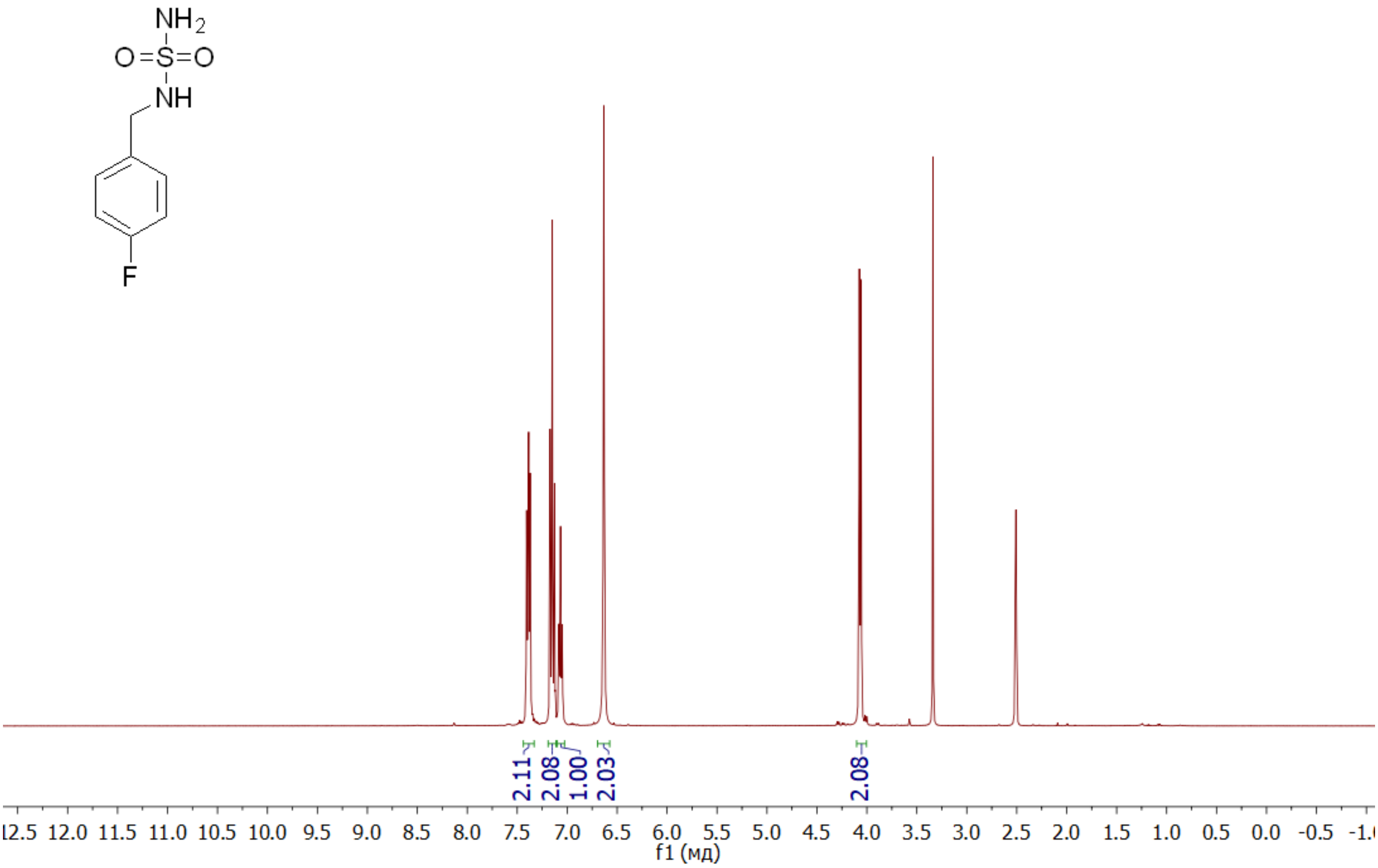

13C/391
PAZc, 391, BF = 125.732643506 MHz, Solvent - DMSO, 13 Jan 2021 T=297 K

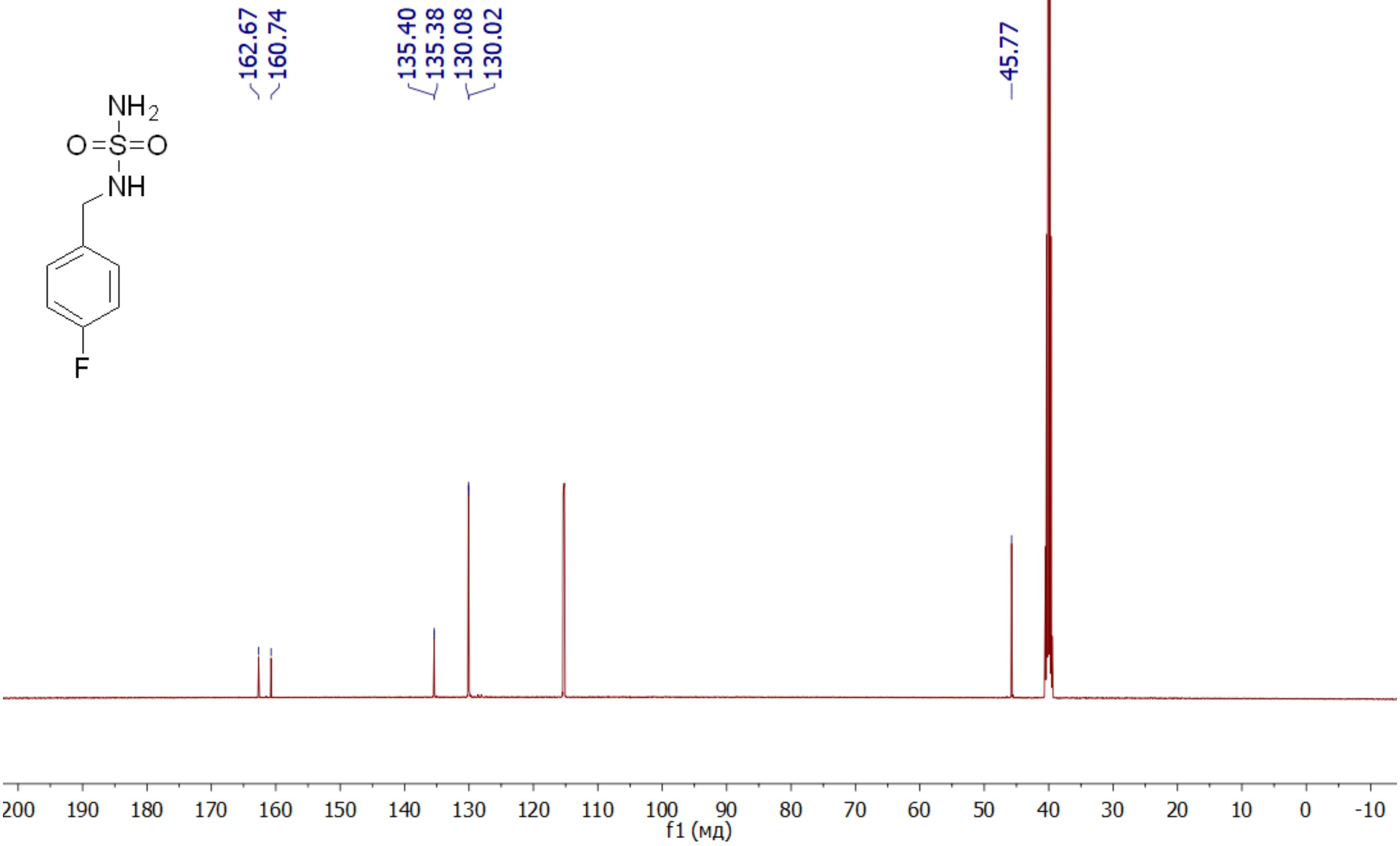

13Dept/391
PAZd, 391, BF = 125.732643506 MHz, Solvent - DMSO, 13 Jan 2021 T=297 K

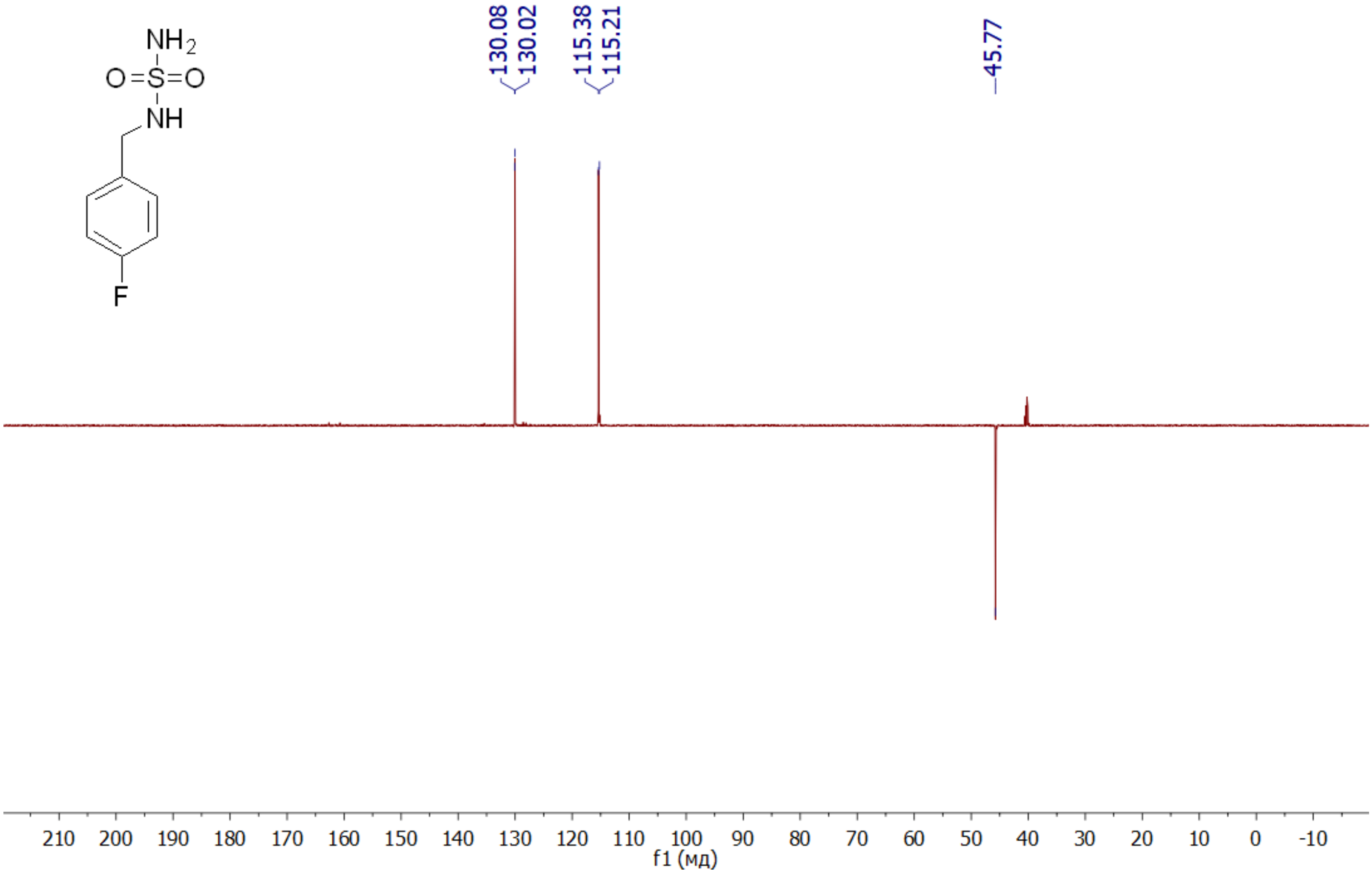

19F/391
PAZf, 391, BF = 470.498283203 MHz, Solvent - DMSO, 13 Jan 2021 T=298 K

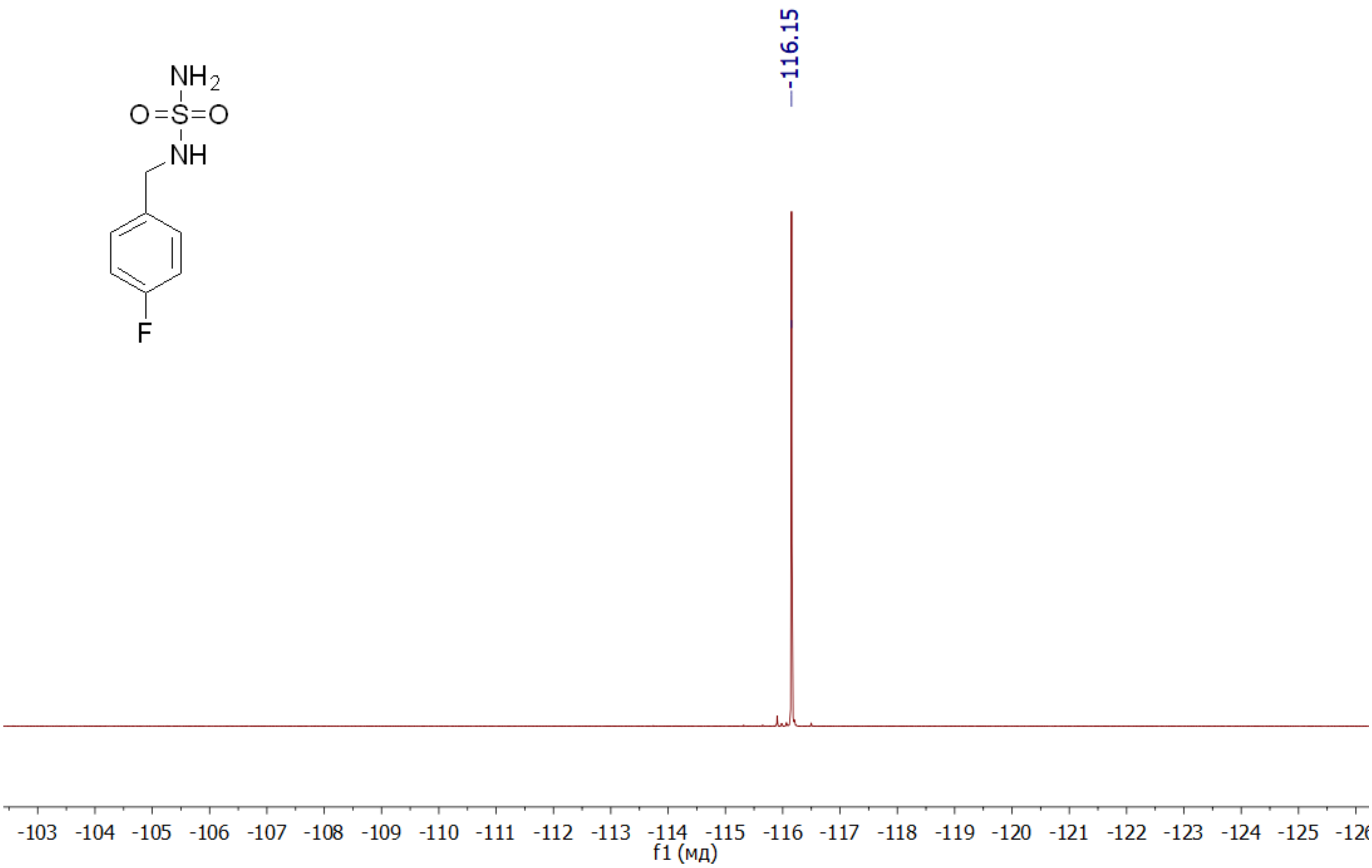

^{1}H NMR and ^{13}C (DEPT) spectra of compound **2d**

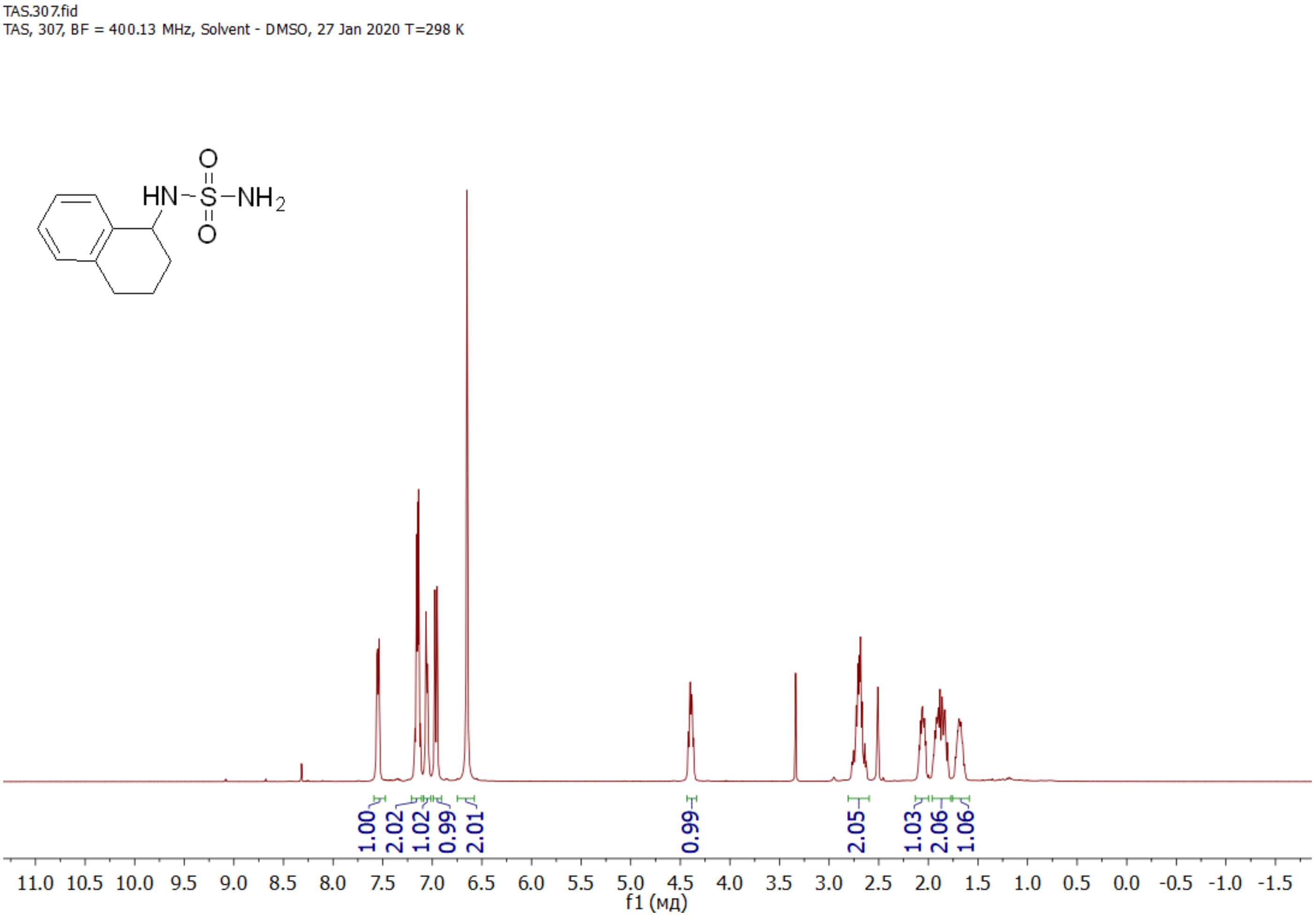

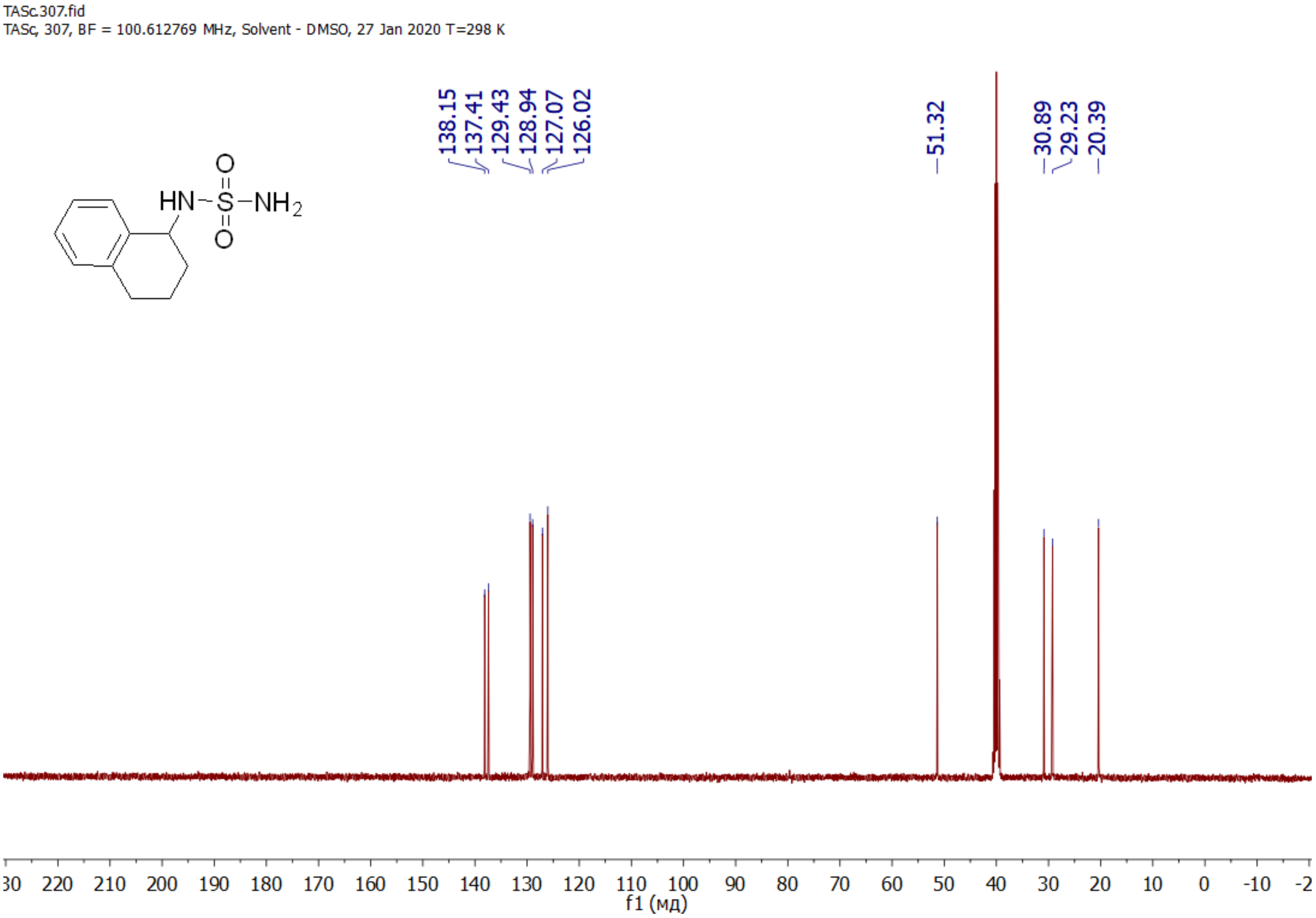

TASd.307.fid
TASd, 307, BF = 100.612769 MHz, Solvent - DMSO, 27 Jan 2020 T=298 K

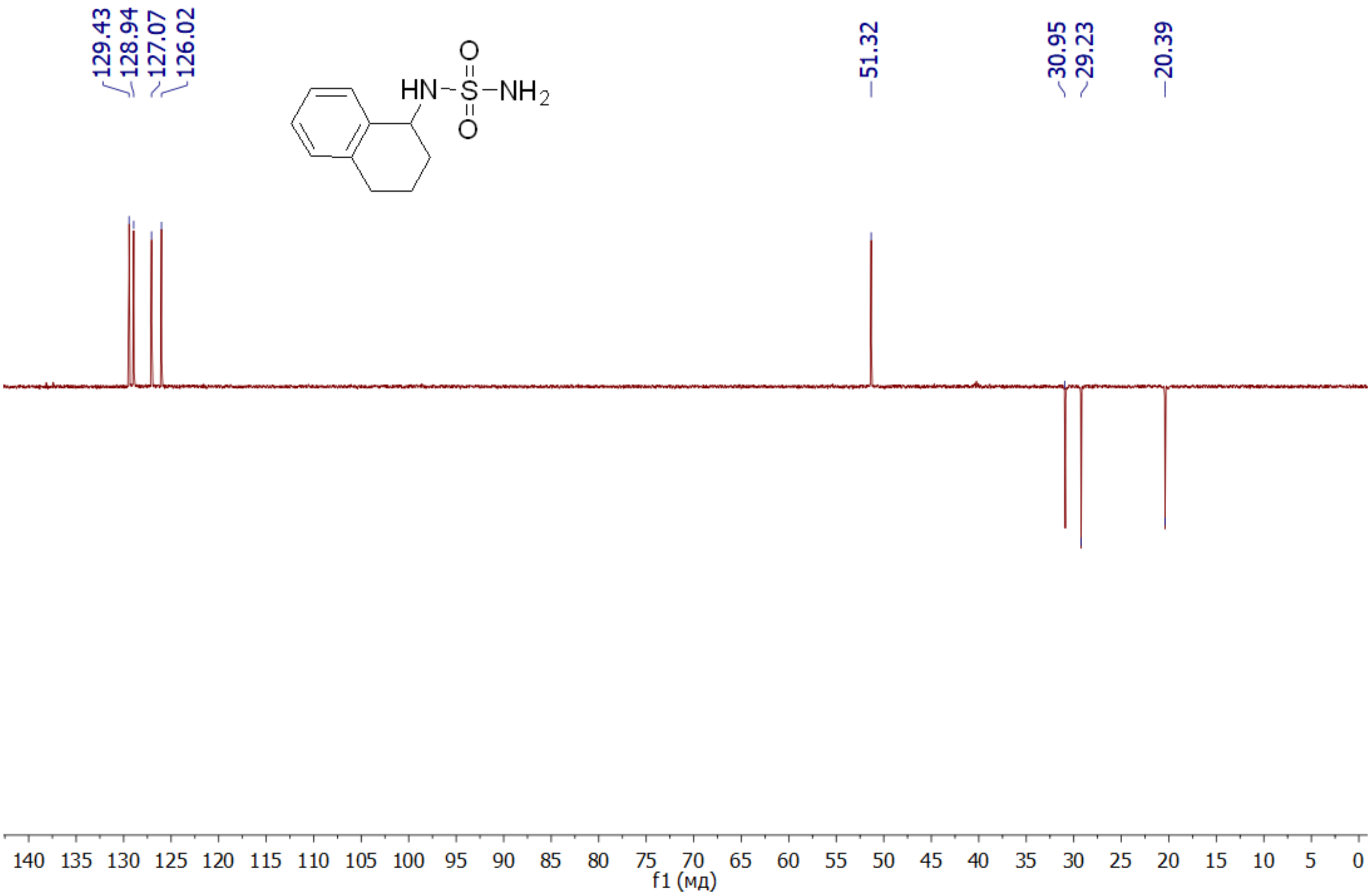

^{1}H NMR and ^{13}C (DEPT) spectra of compound **2e**

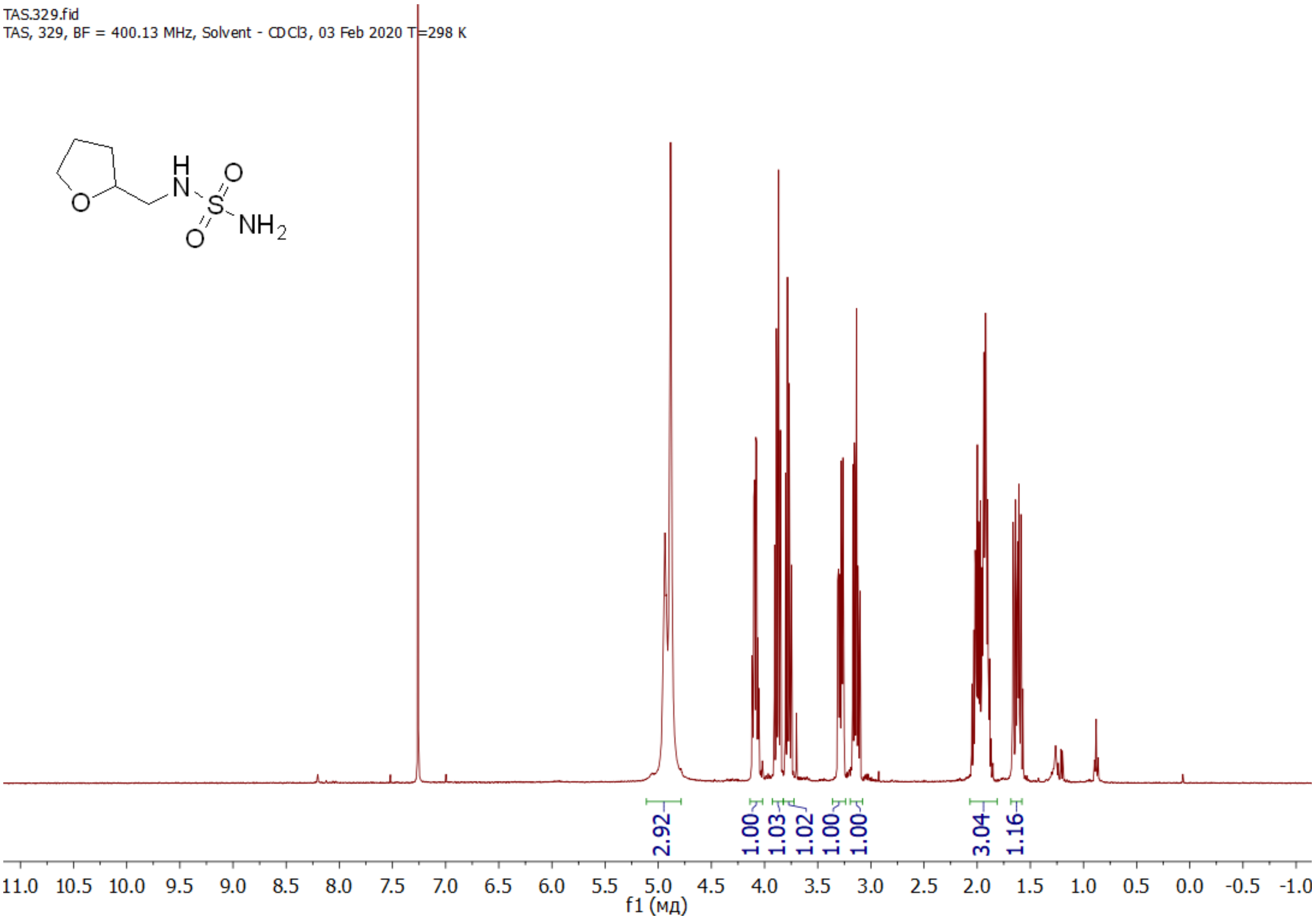

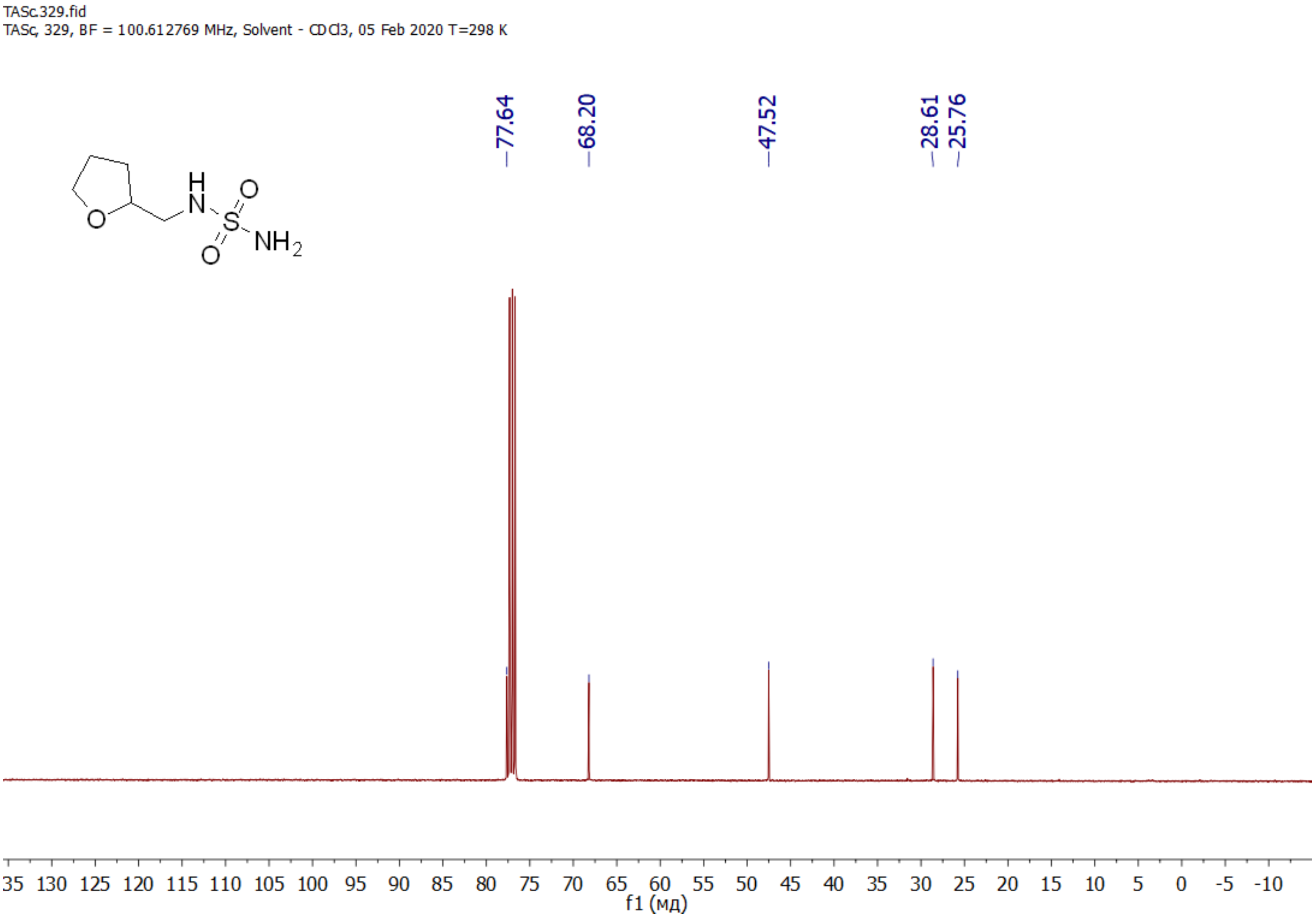

TASd.329.fid
TASd, 329, BF = 100.612769 MHz, Solvent - CDCl3, 05 Feb 2020 T=298 K

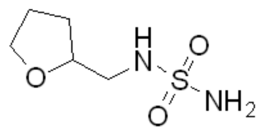

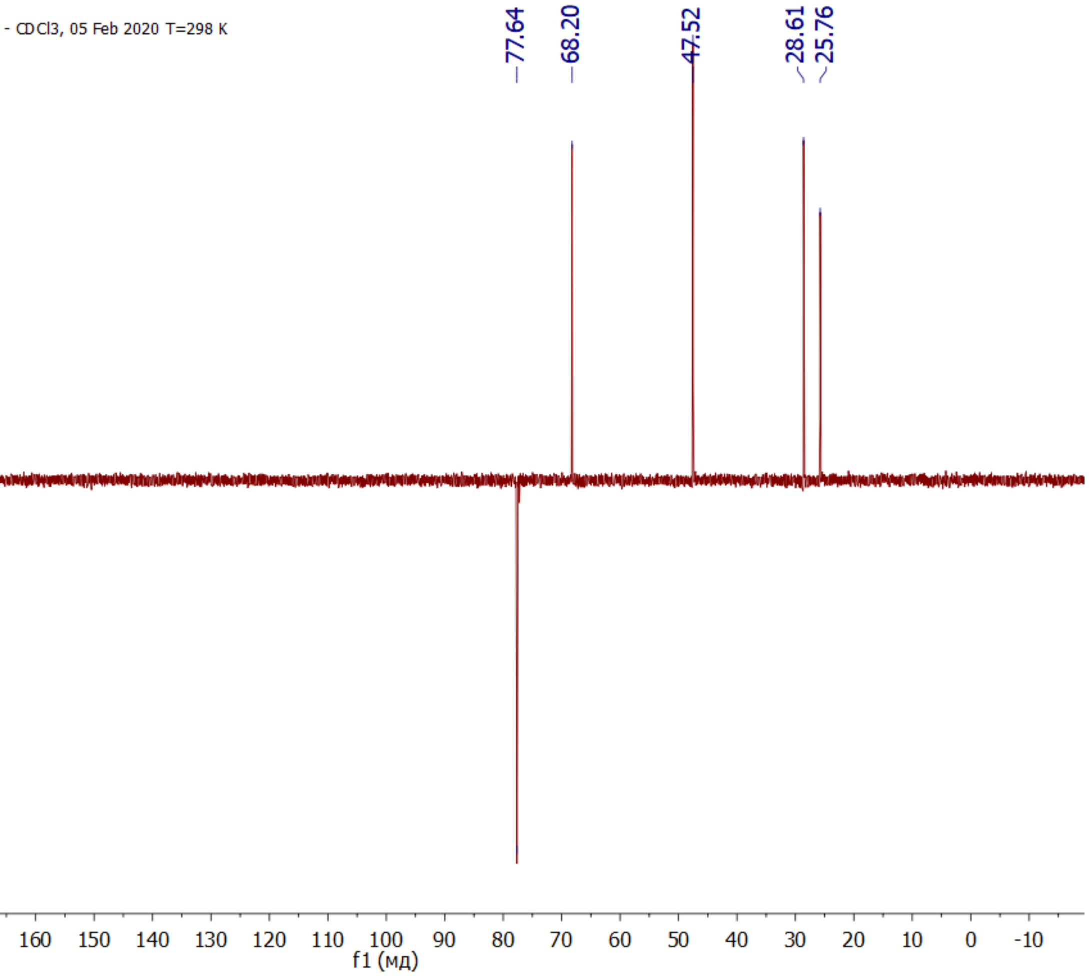

^{1}H NMR and ^{13}C (DEPT) spectra of compound **2f**

PAZ.355.fid
PAZ, 355, BF = 400.13 MHz, Solvent - DMSO, 10 Dec 2019 T=298 K

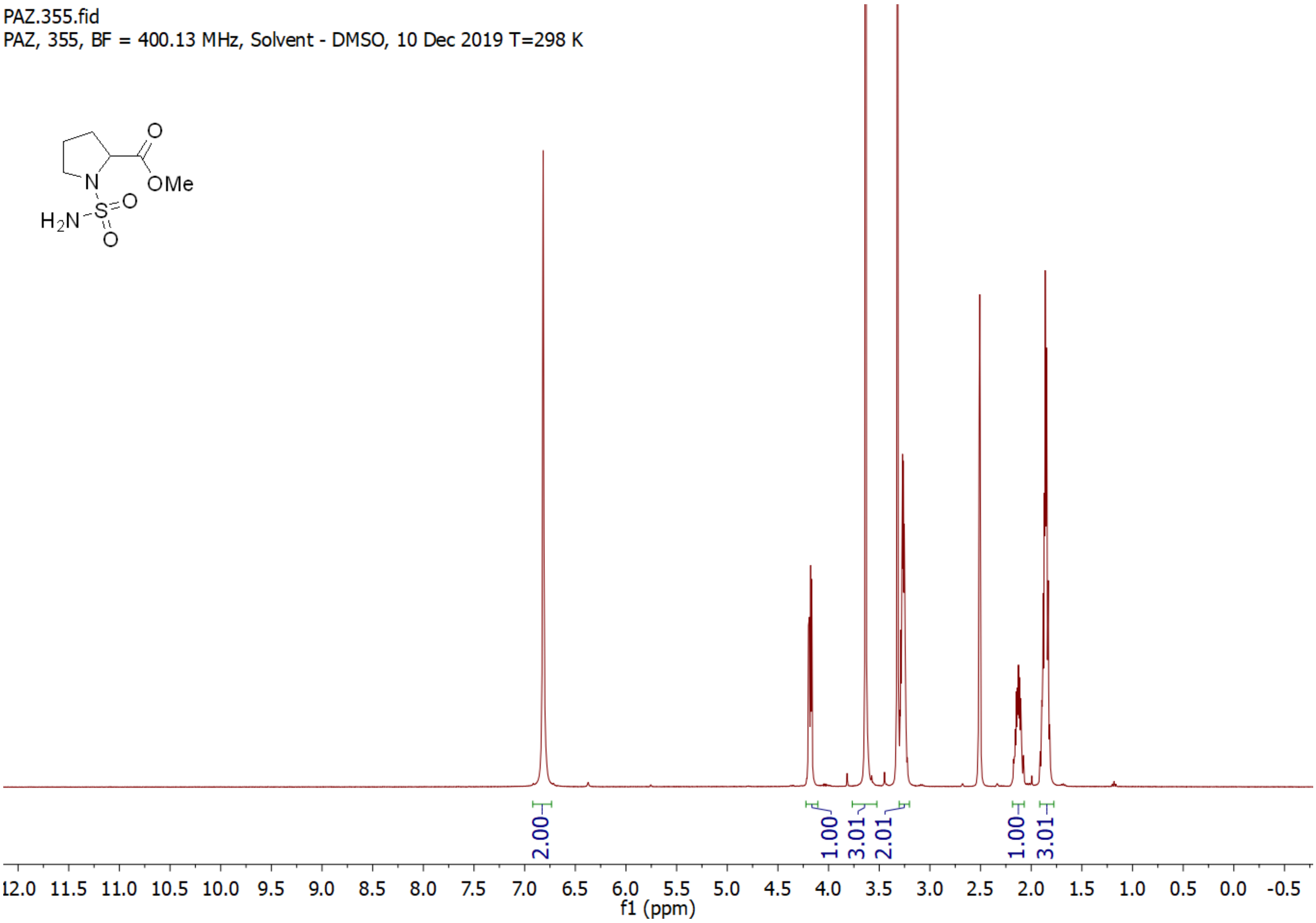

PAZc.355.fid
PAZc, 355, BF = 100.612769 MHz, Solvent - DMSO, 18 Dec 2019 T=298 K

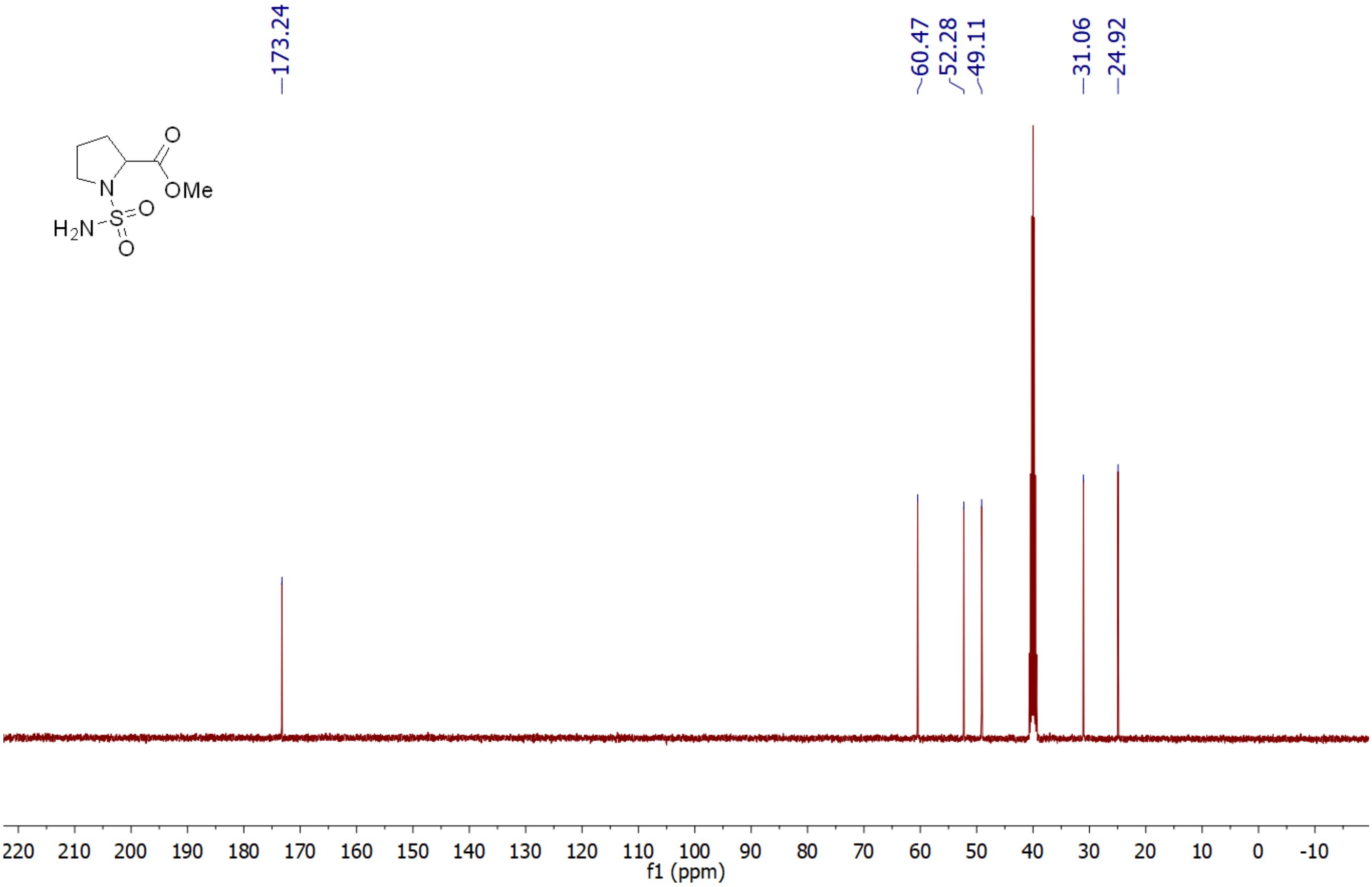

PAZd.355.fid
PAZd, 355, BF = 100.612769 MHz, Solvent - DMSO, 18 Dec 2019 T=298 K

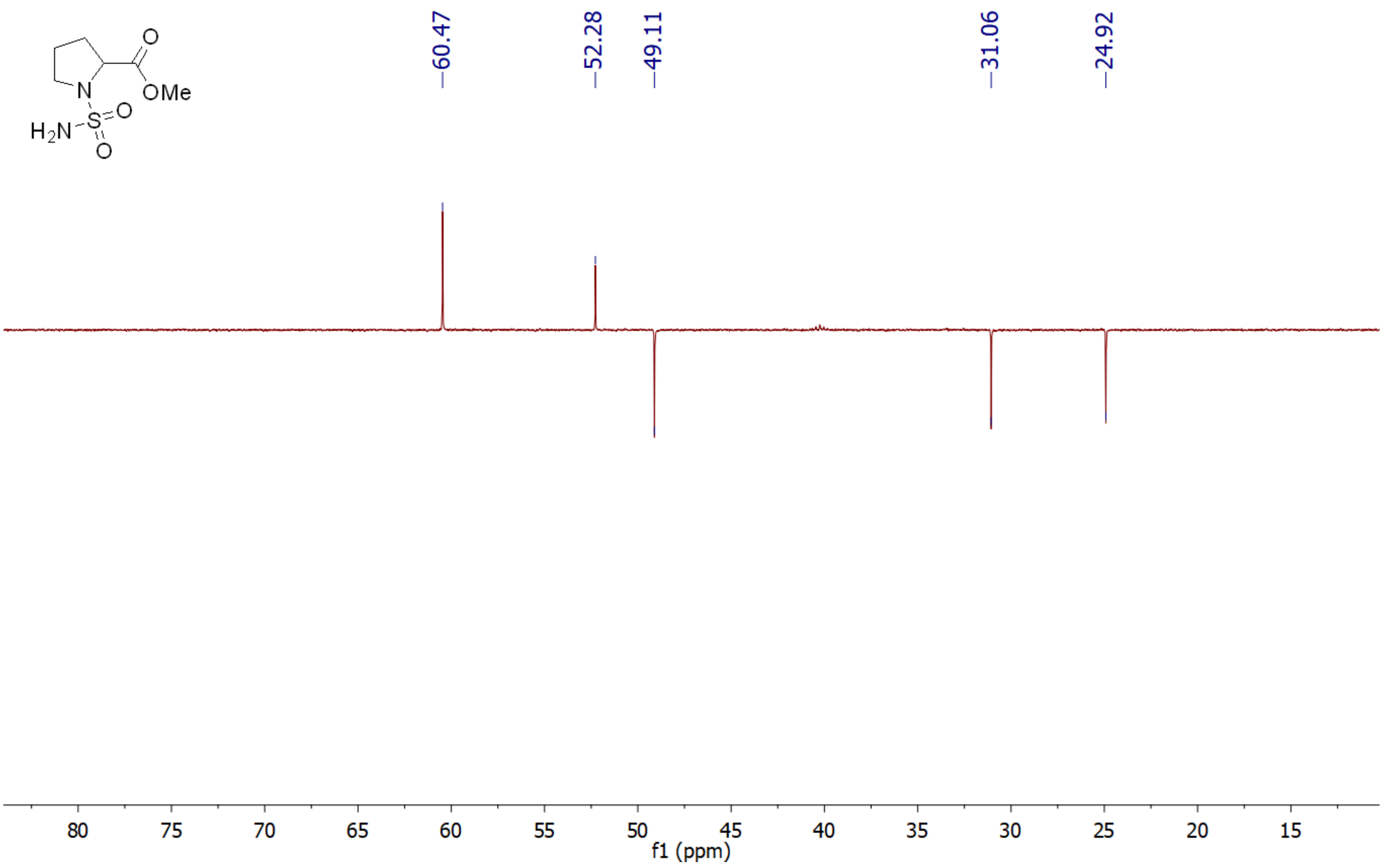

^{1}H NMR and ^{13}C spectra of compound **2g**

TAS.293.fid
TAS, 293, BF = 400.13 MHz, Solvent - DMSOmixt, 13 Dec 2019 T=298 K

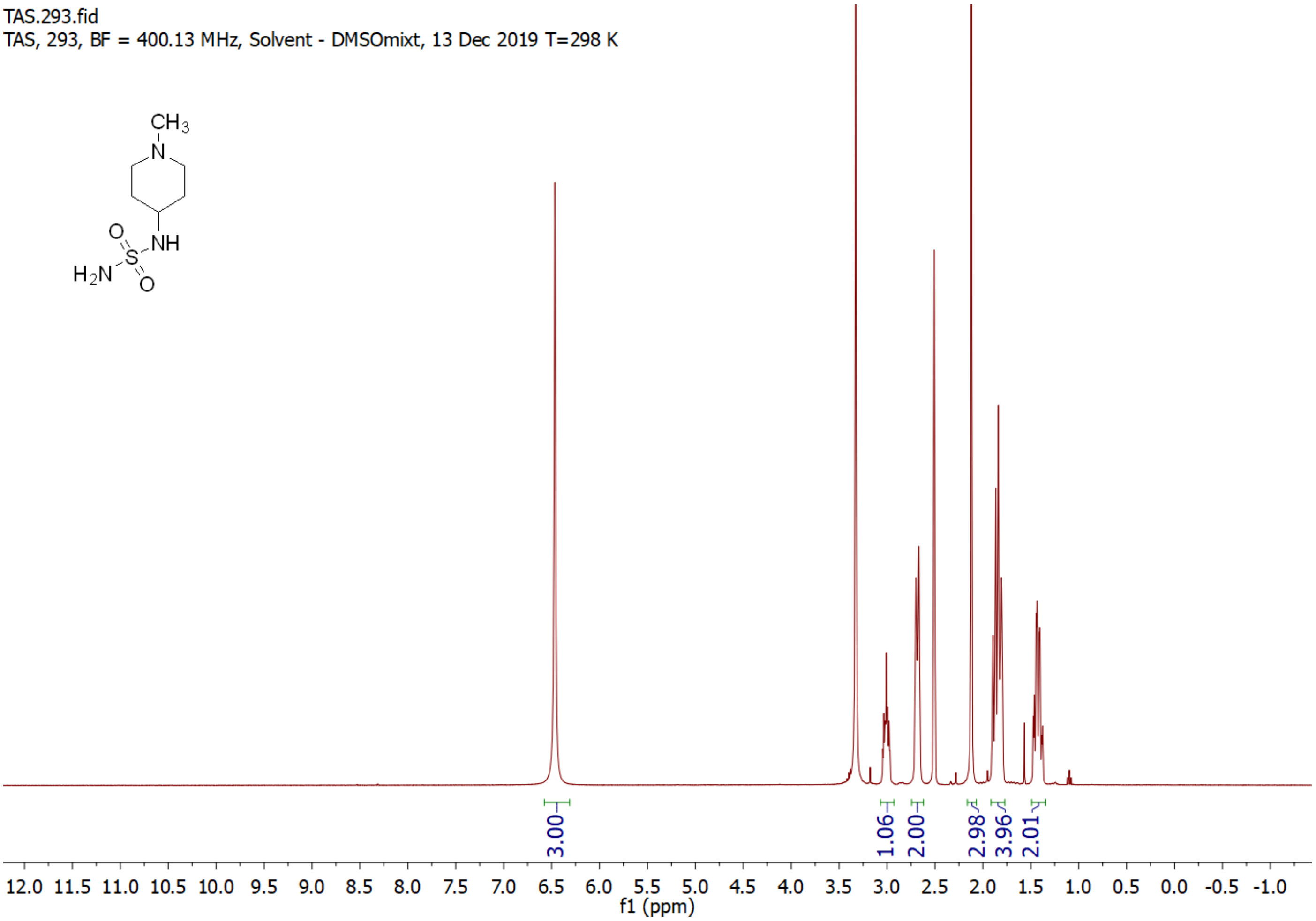

TASc.293.fid
TASc, 293, BF = 100.612769 MHz, Solvent - DMSO, 15 Dec 2019 T=298 K

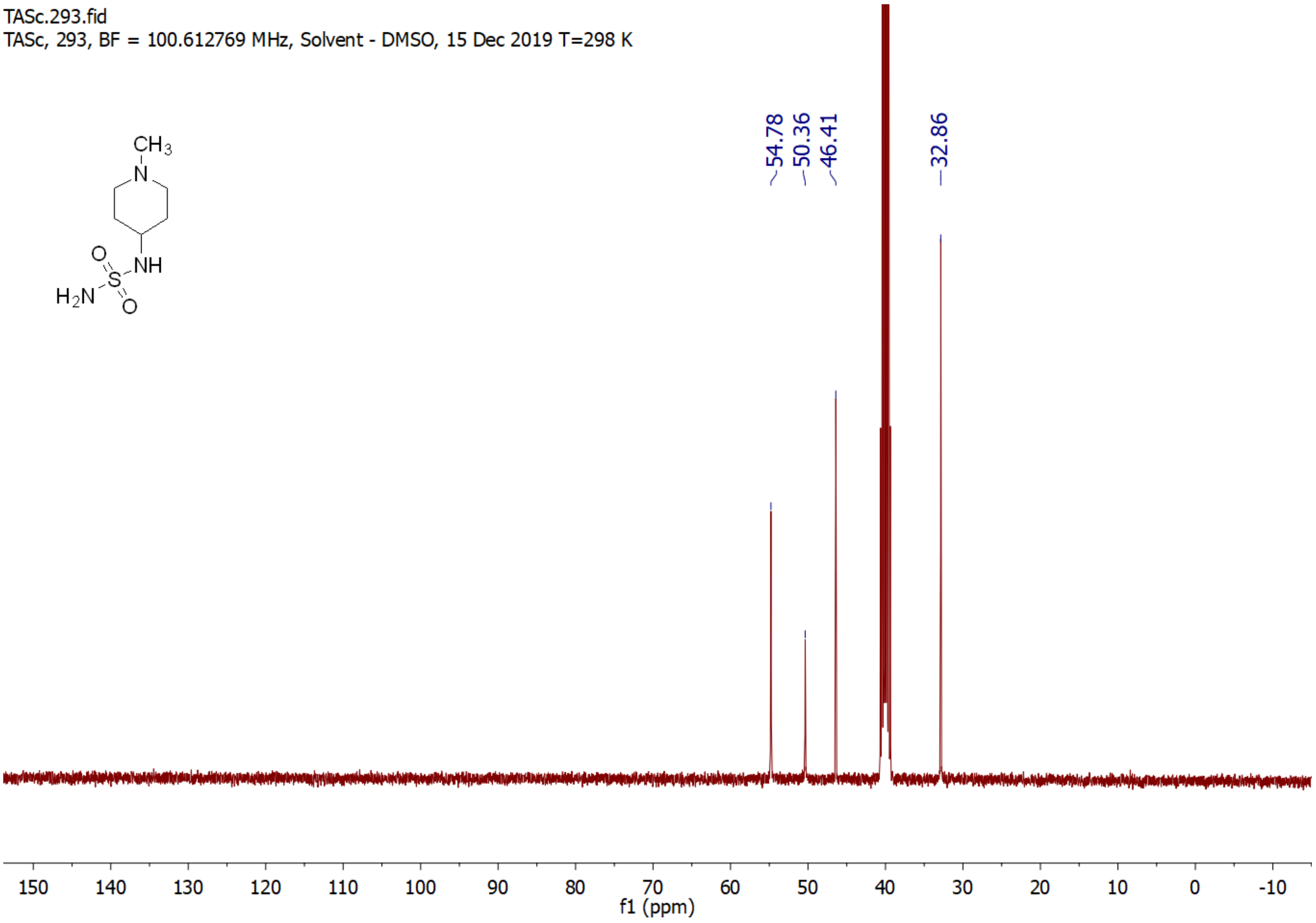

^{1}H NMR and ^{13}C (DEPT) spectra of compound **2h**

PAZ.342.fid
PAZ, 342, BF = 400.13 MHz, Solvent - DMSO, 05 Dec 2019 T=298 K

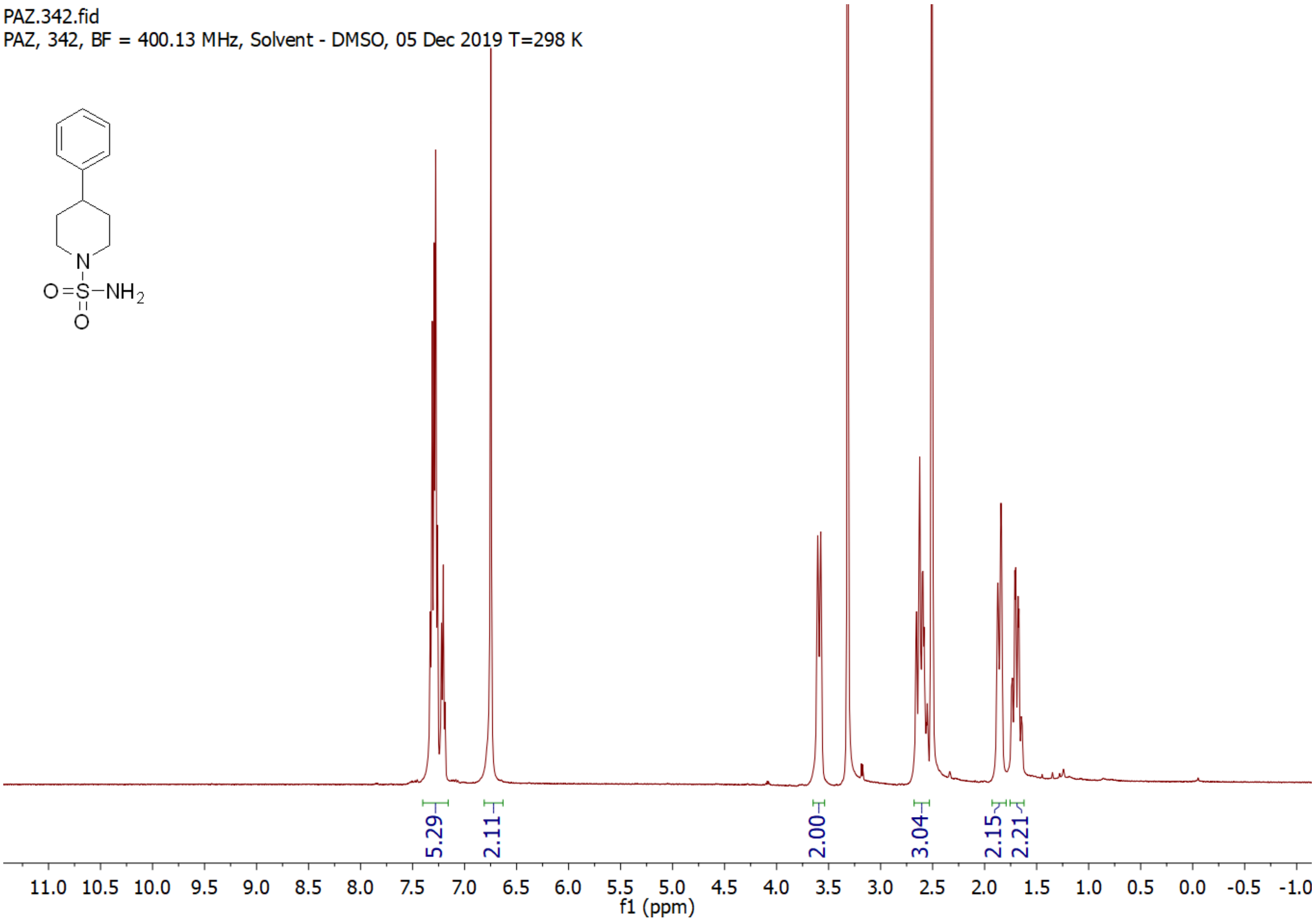

PAZc.342.fid
PAZc, 342, BF = 100.612769 MHz, Solvent - DMSO, 10 Dec 2019 T=298 K

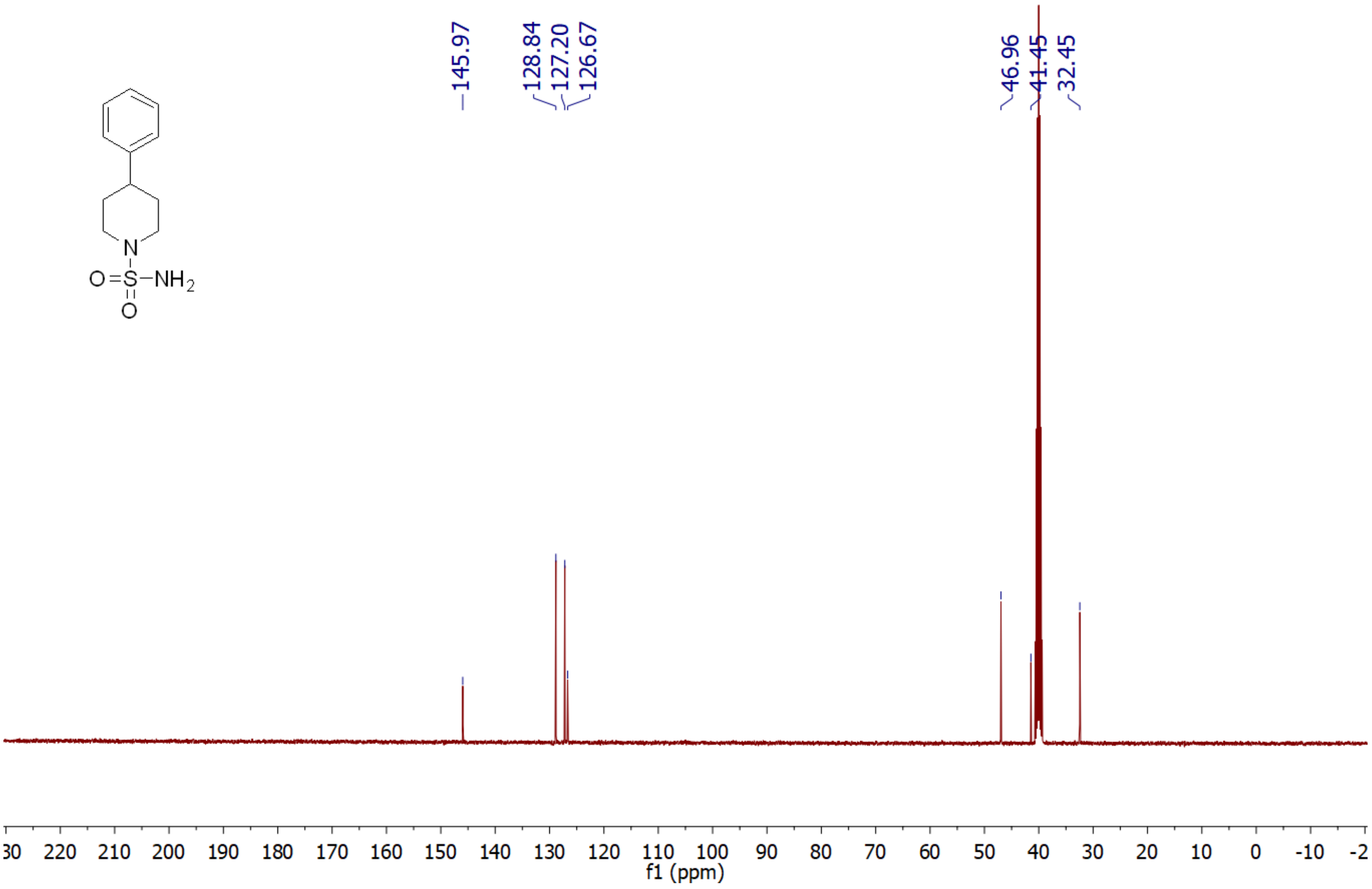

PAZd.342.fid
PAZd, 342, BF = 100.612769 MHz, Solvent - DMSO, 10 Dec 2019 T=298 K

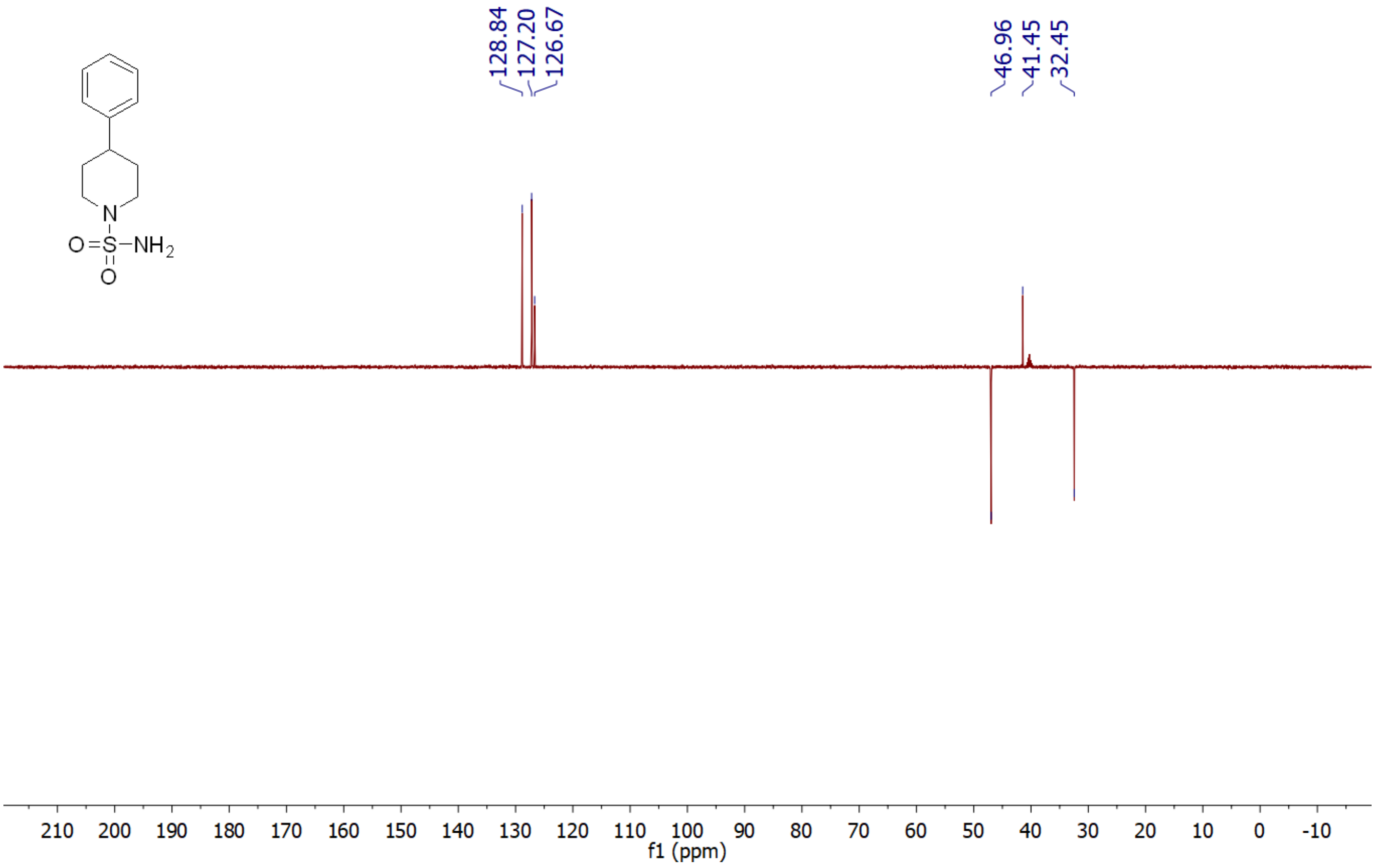

^{1}H NMR and ^{13}C (DEPT) spectra of compound **2i**

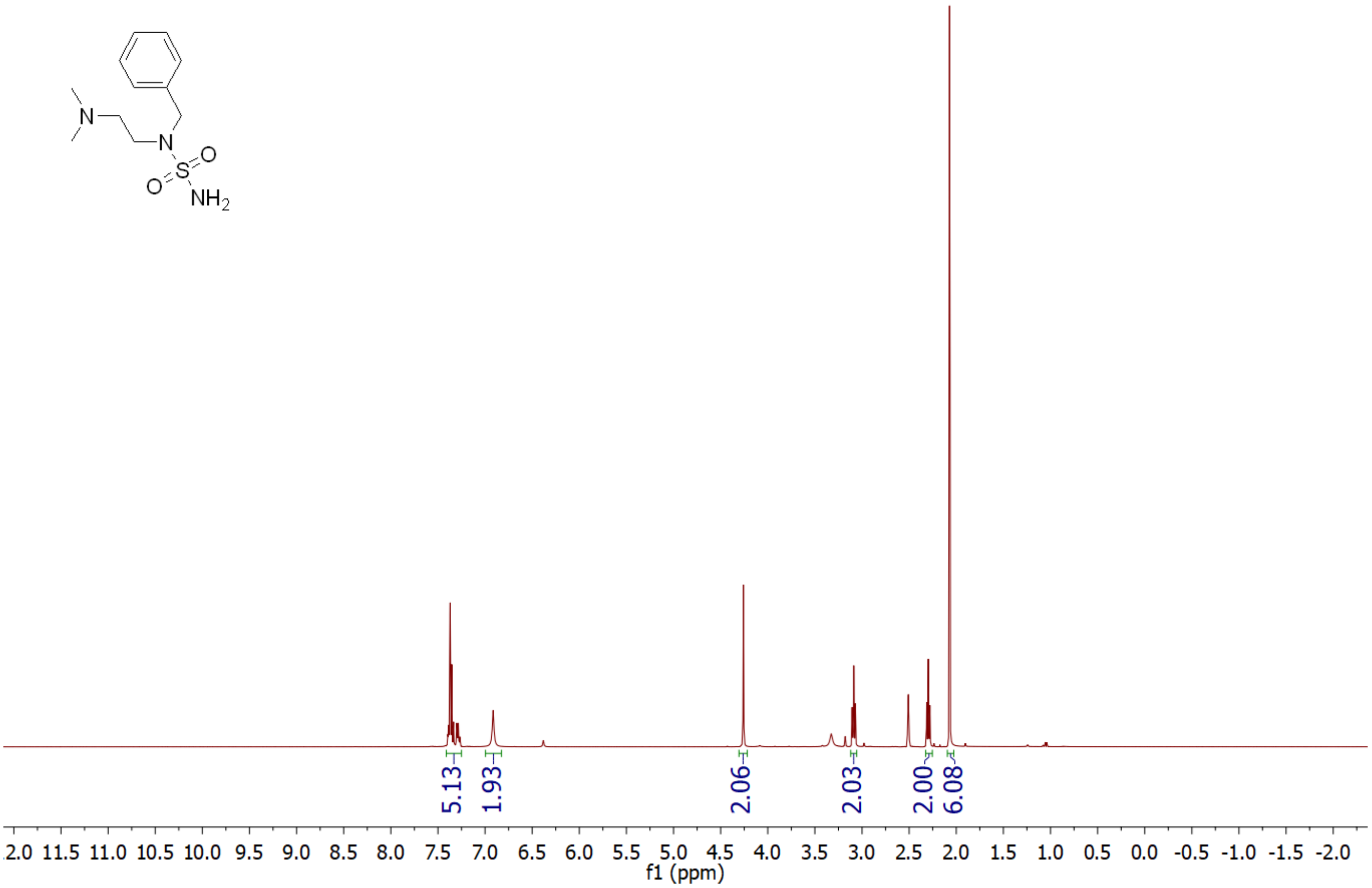

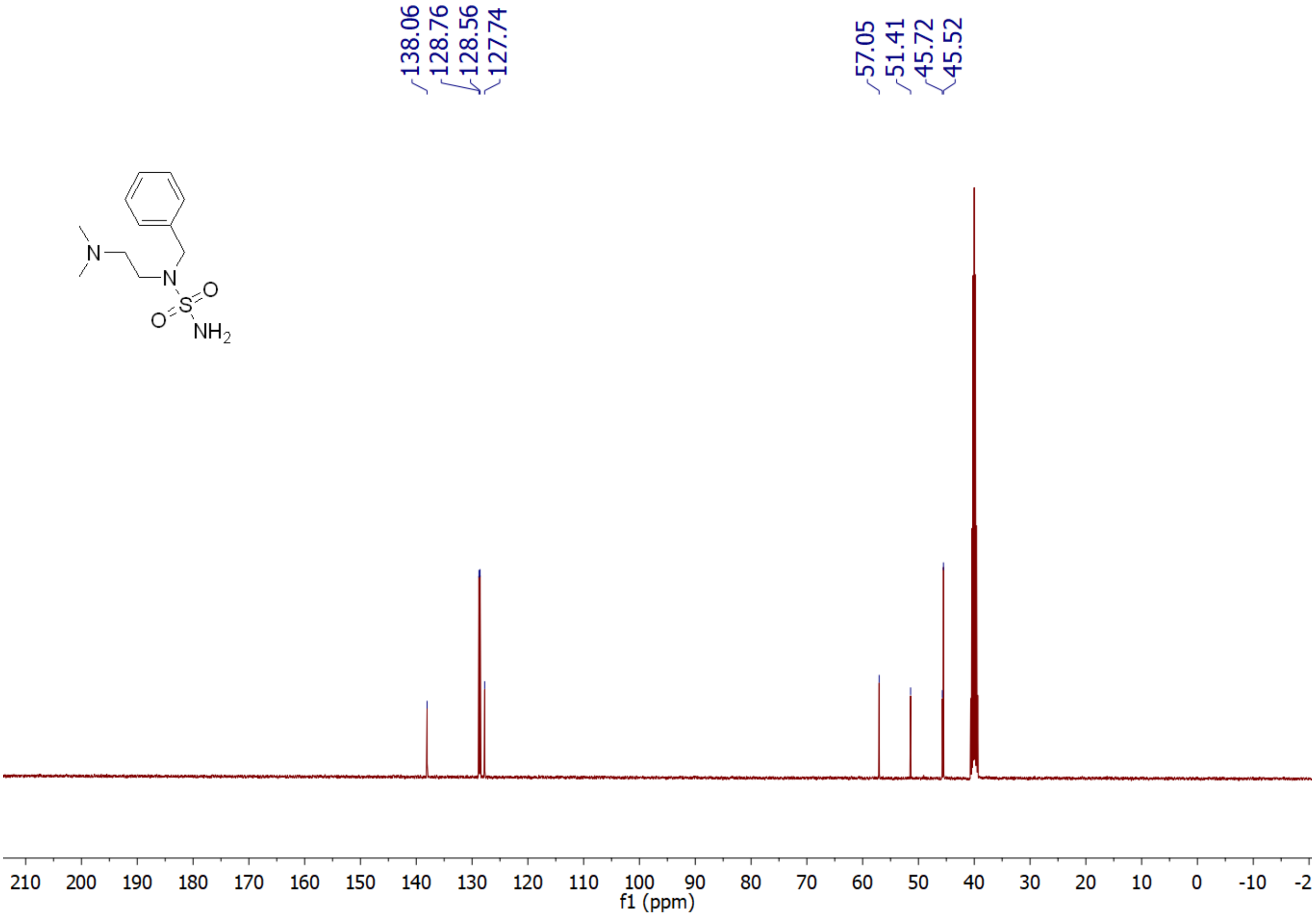

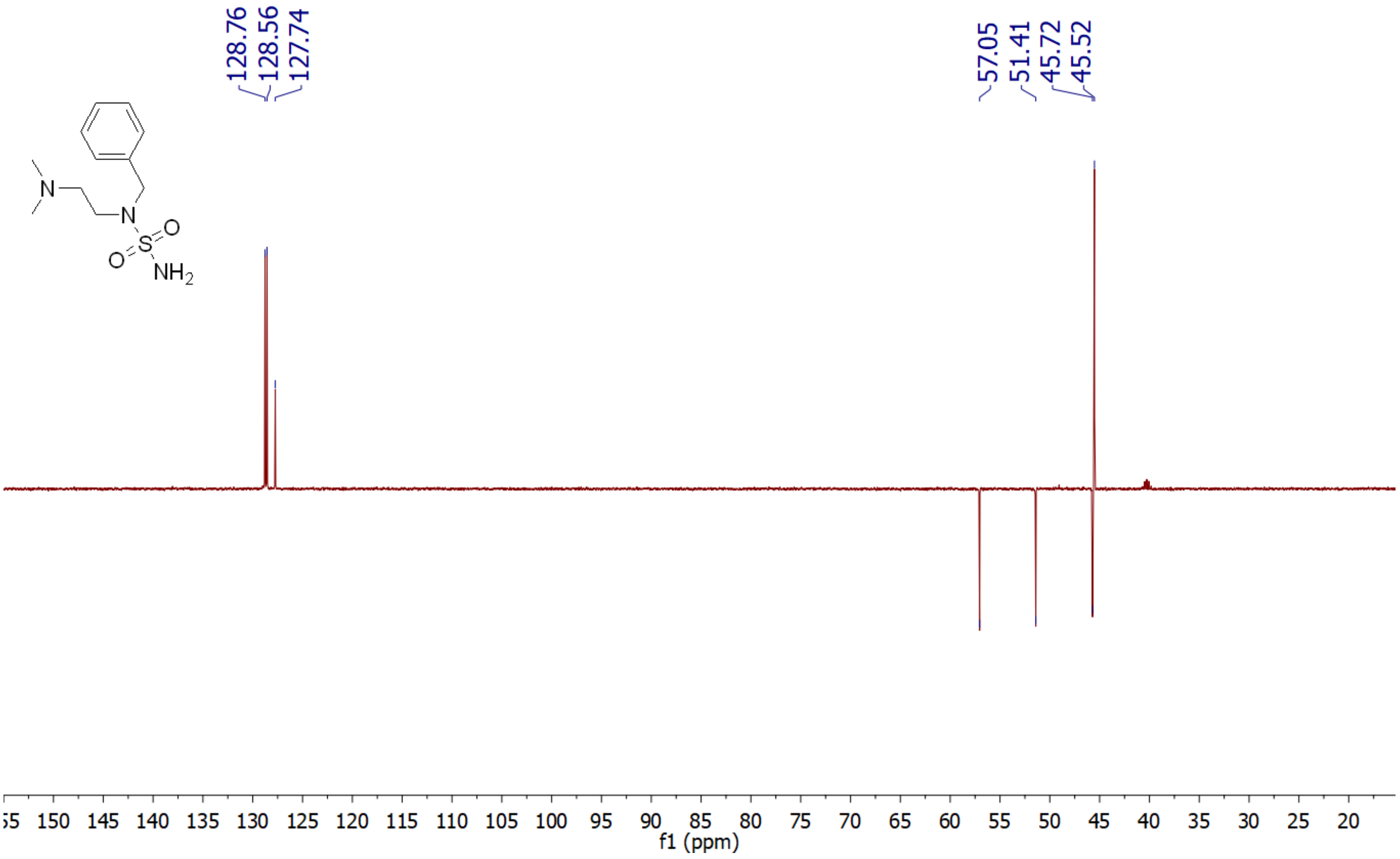

128.76
128.56
127.74
57.05
51.41
45.72
45.52
N
N
S
O
O
NH2
55 150 145 140 135 130 125 120 115 110 105 100 95 90 85 80 75 70 65 60 55 50 45 40 35 30 25 20
f1 (ppm)

^{1}H NMR and ^{13}C (DEPT) spectra of compound **2j**

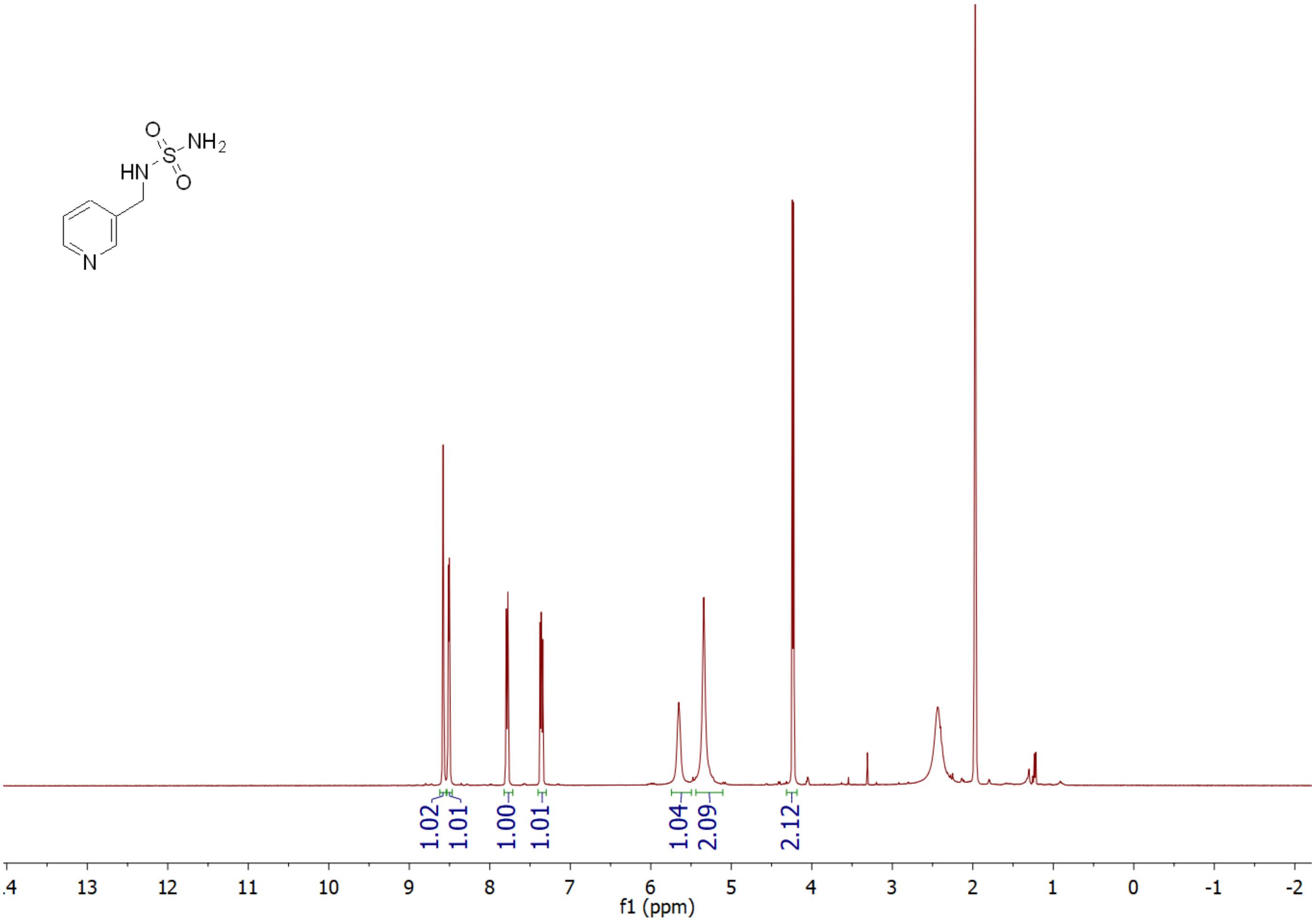

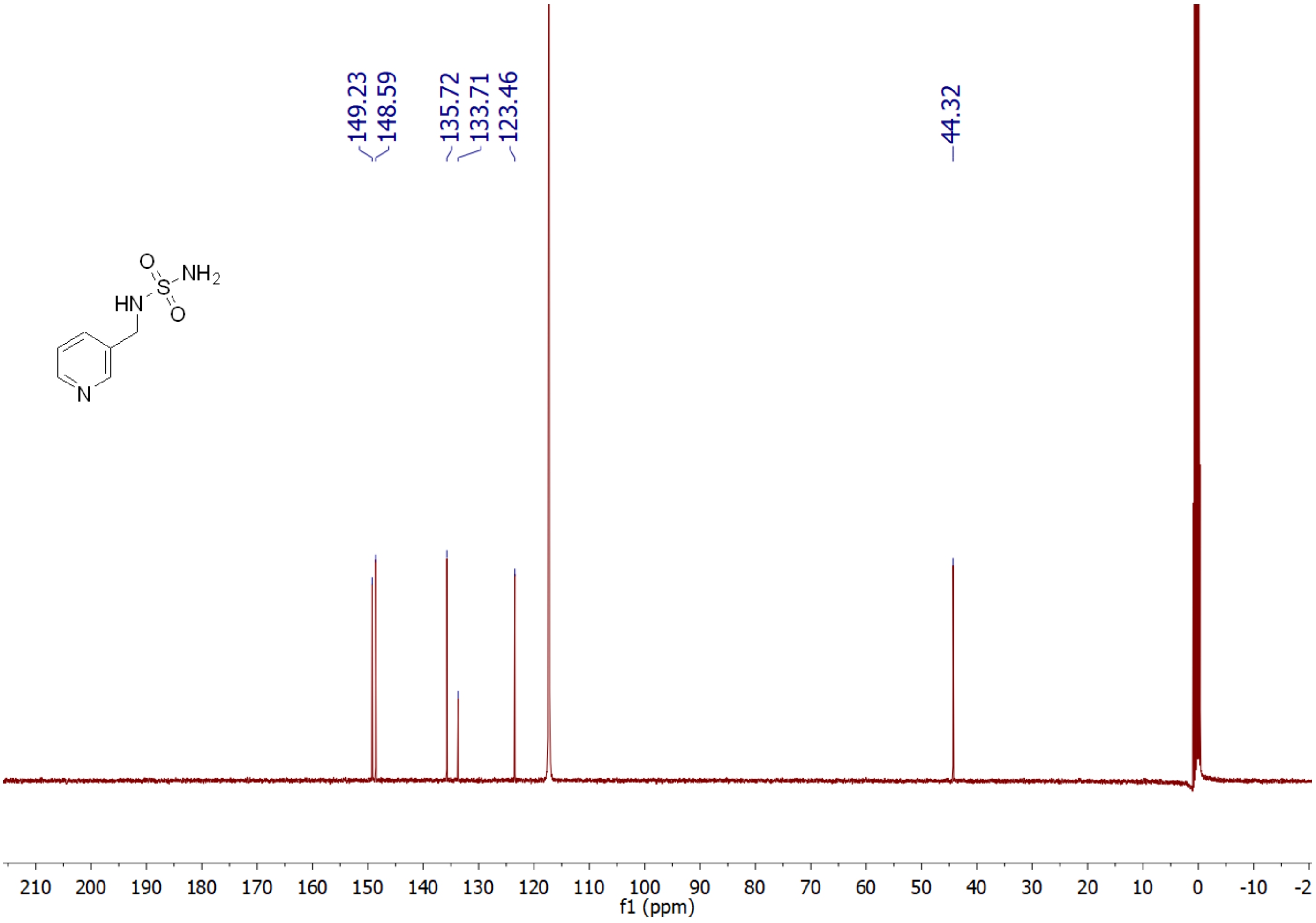

^{1}H NMR and ^{13}C (DEPT) spectra of compound **2k**

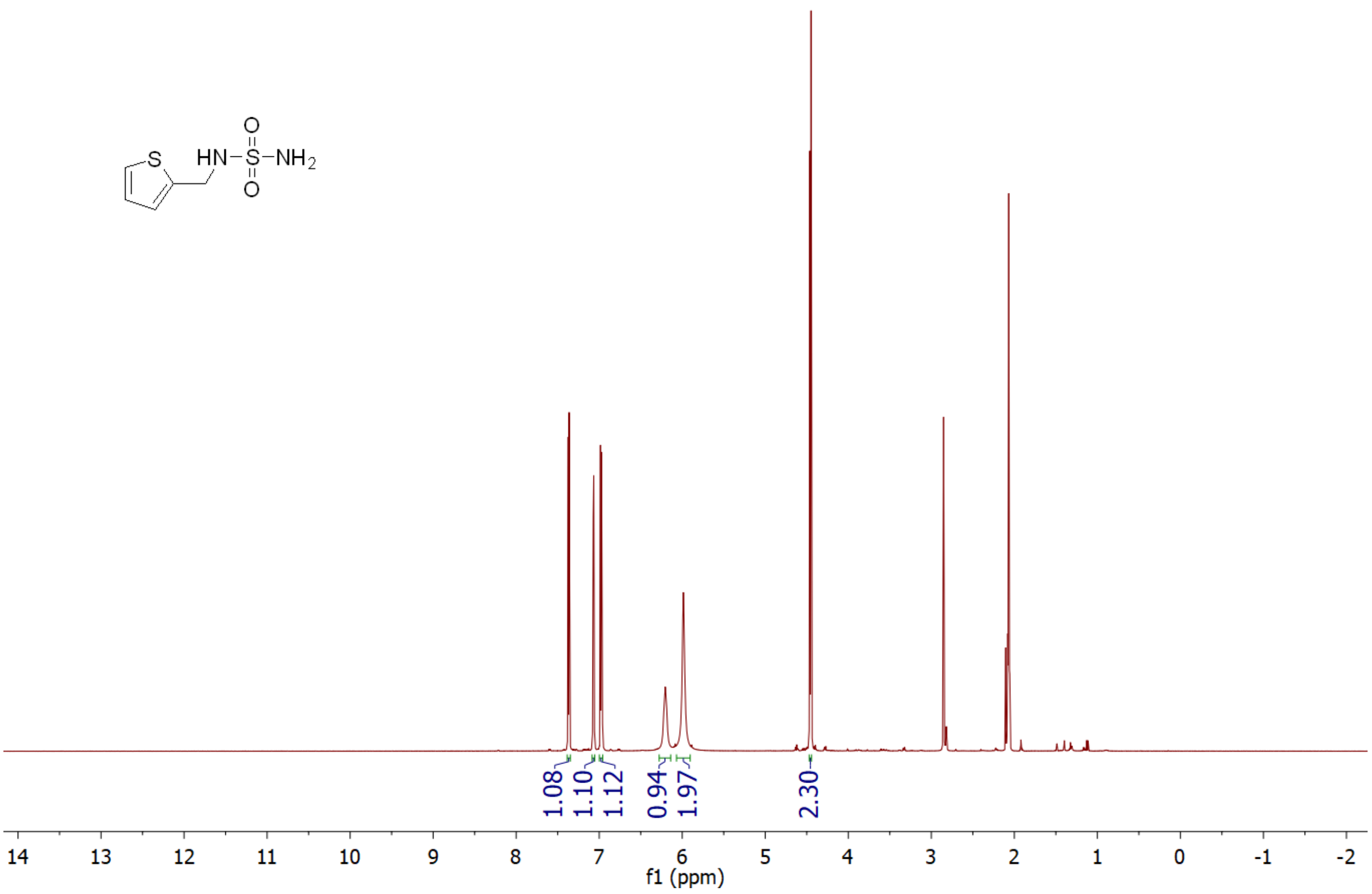

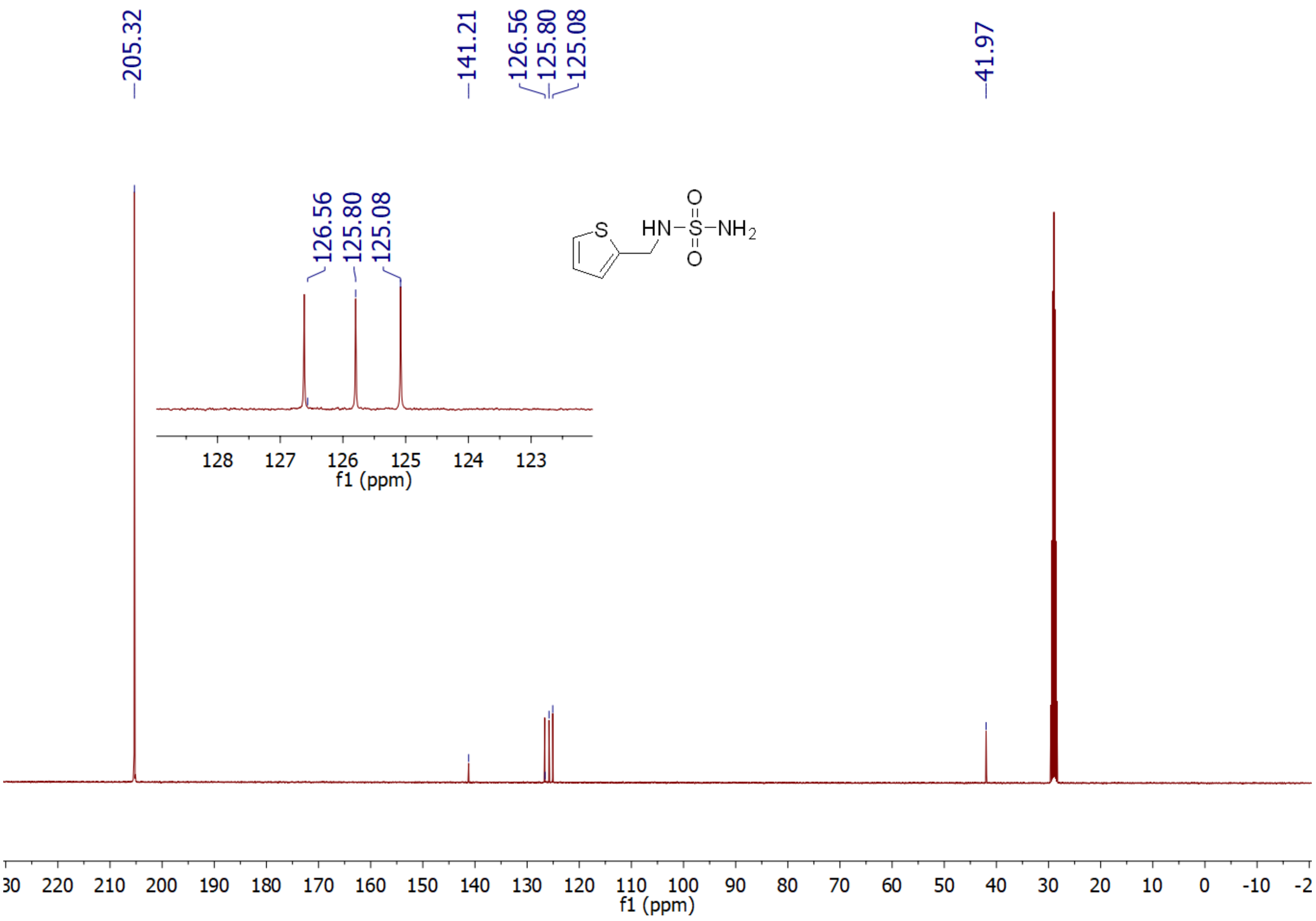

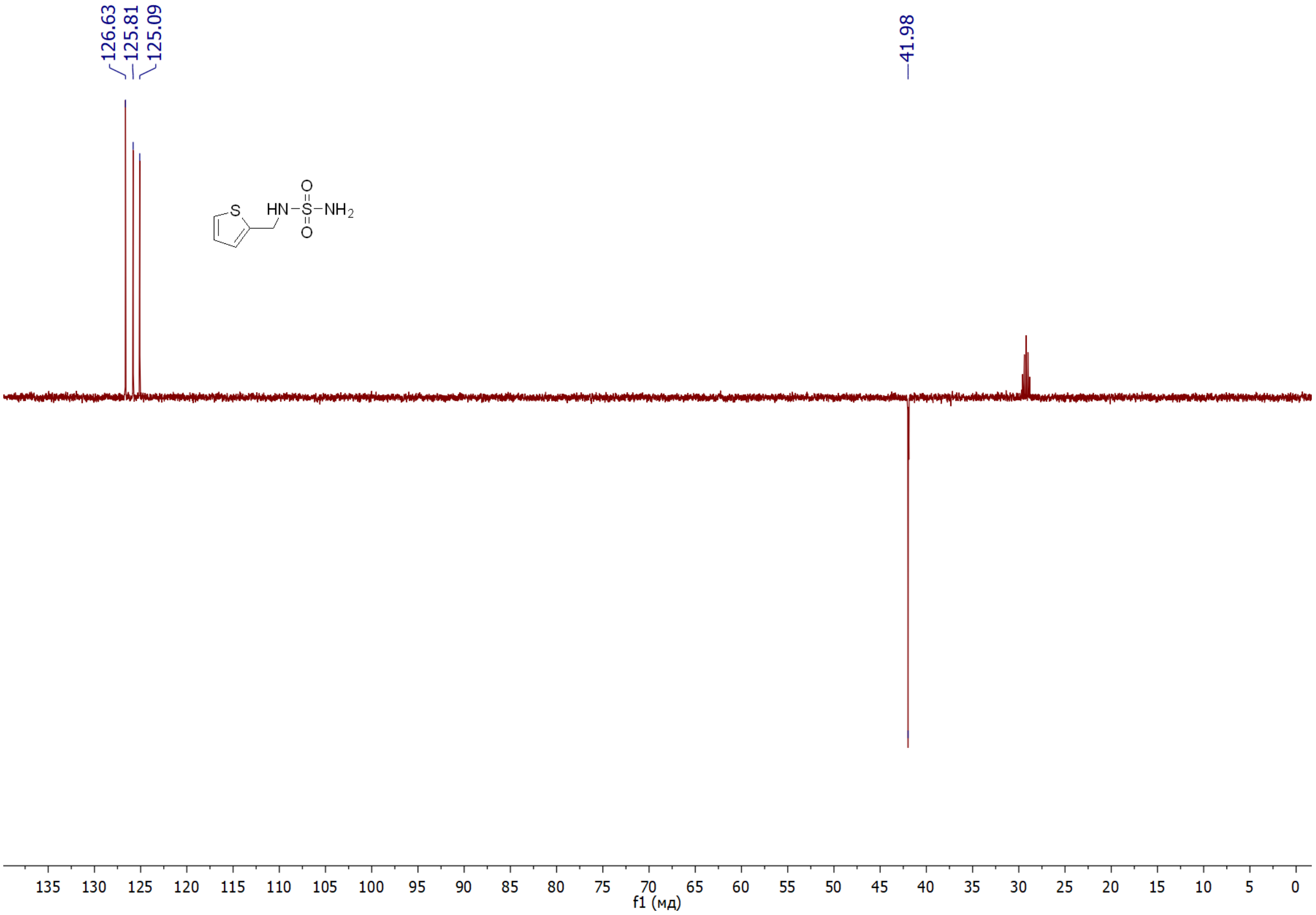

126.63
125.81
125.09
41.98
S
HN
S
O
O
NH2
135
130
125
120
115
110
105
100
95
90
85
80
75
70
65
60
55
50
45
40
35
30
25
20
15
10
5
0
f1 (мд)

^{1}H NMR and ^{13}C (DEPT) spectra of compound **2l**

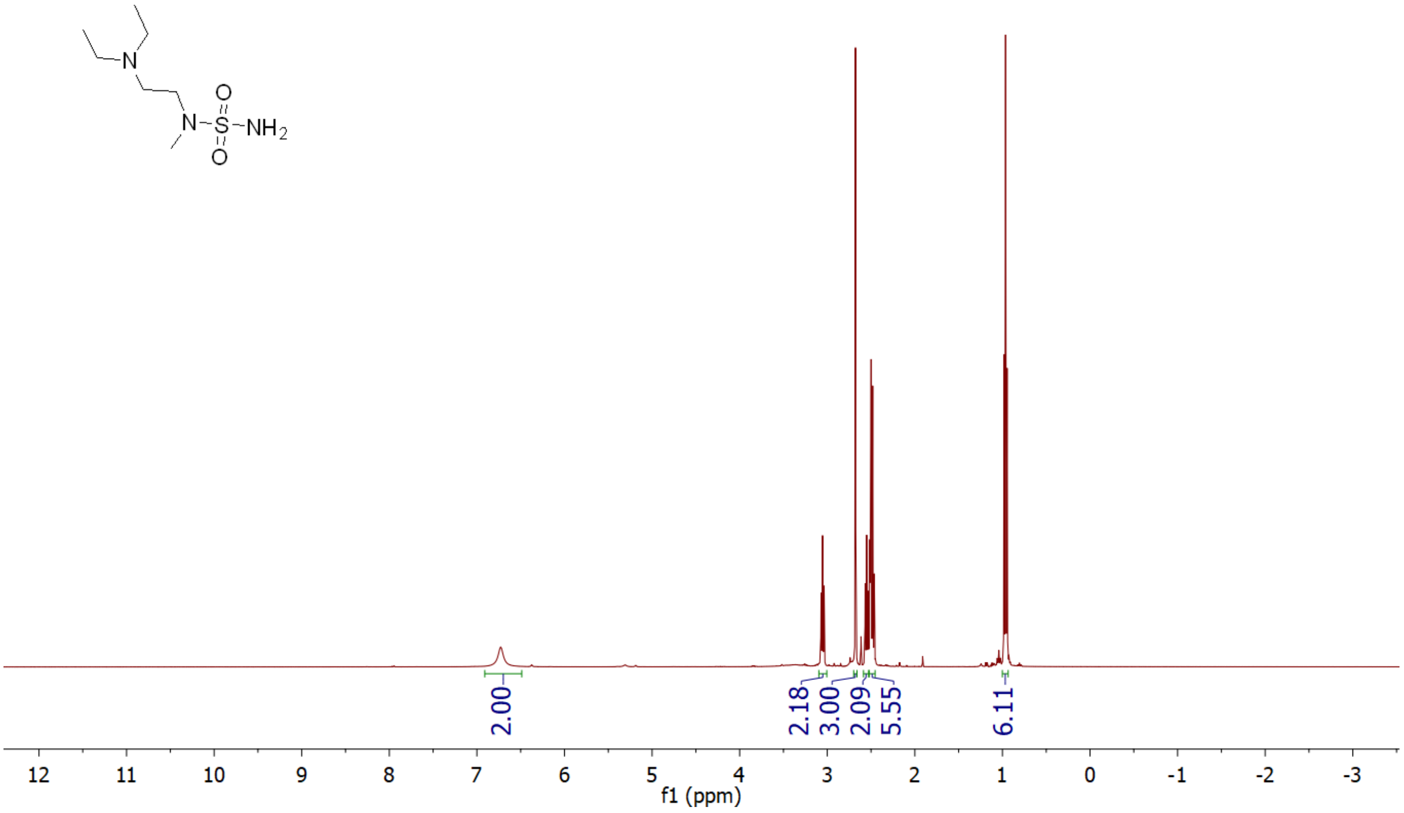

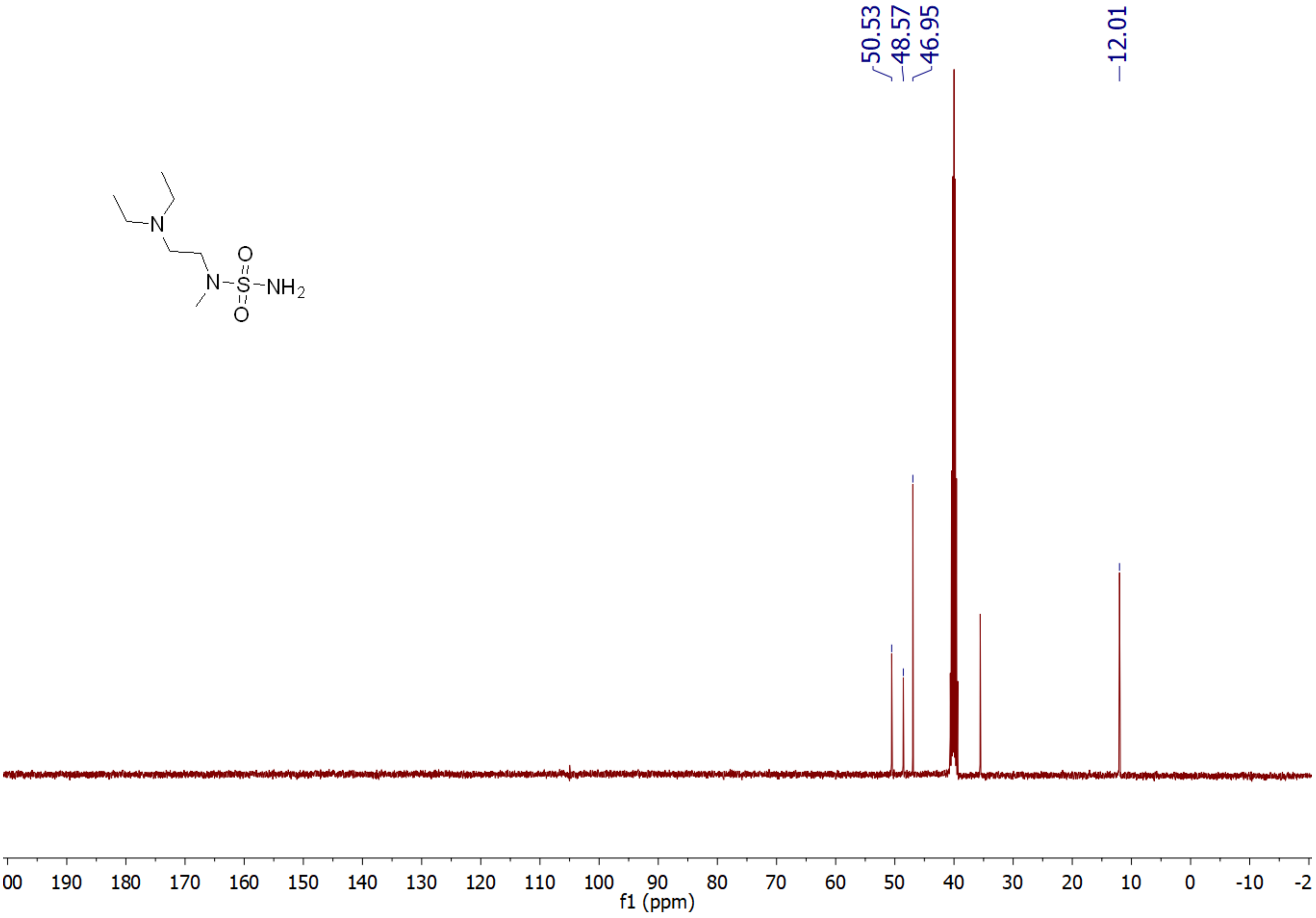

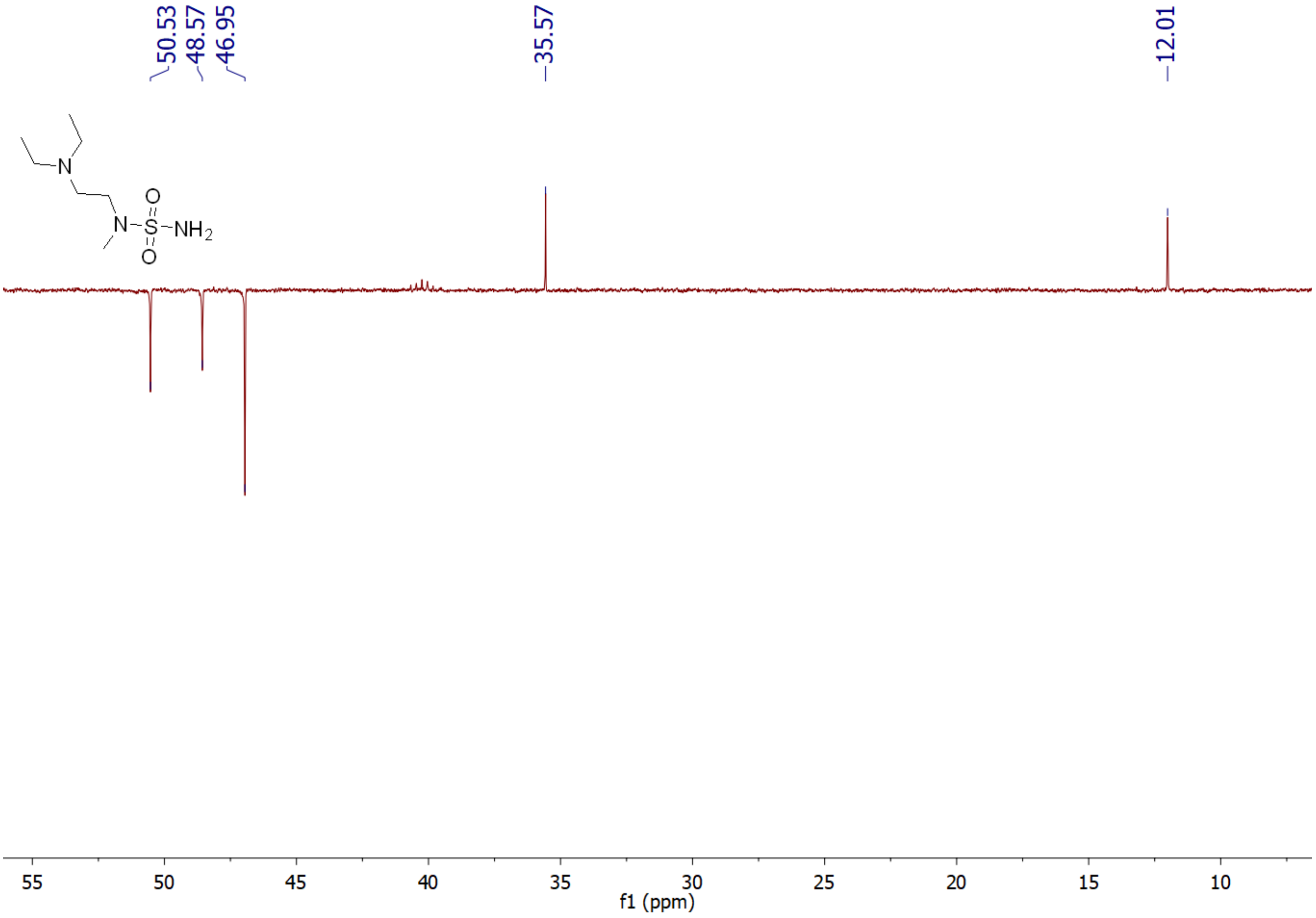
50.53
48.57
46.95
35.57
12.01
O
N
S
NH2
O
N
55
50
45
40
35
30
25
20
15
10
f1 (ppm)

^1H NMR and ^{13}C (DEPT) spectra of compound **2m**

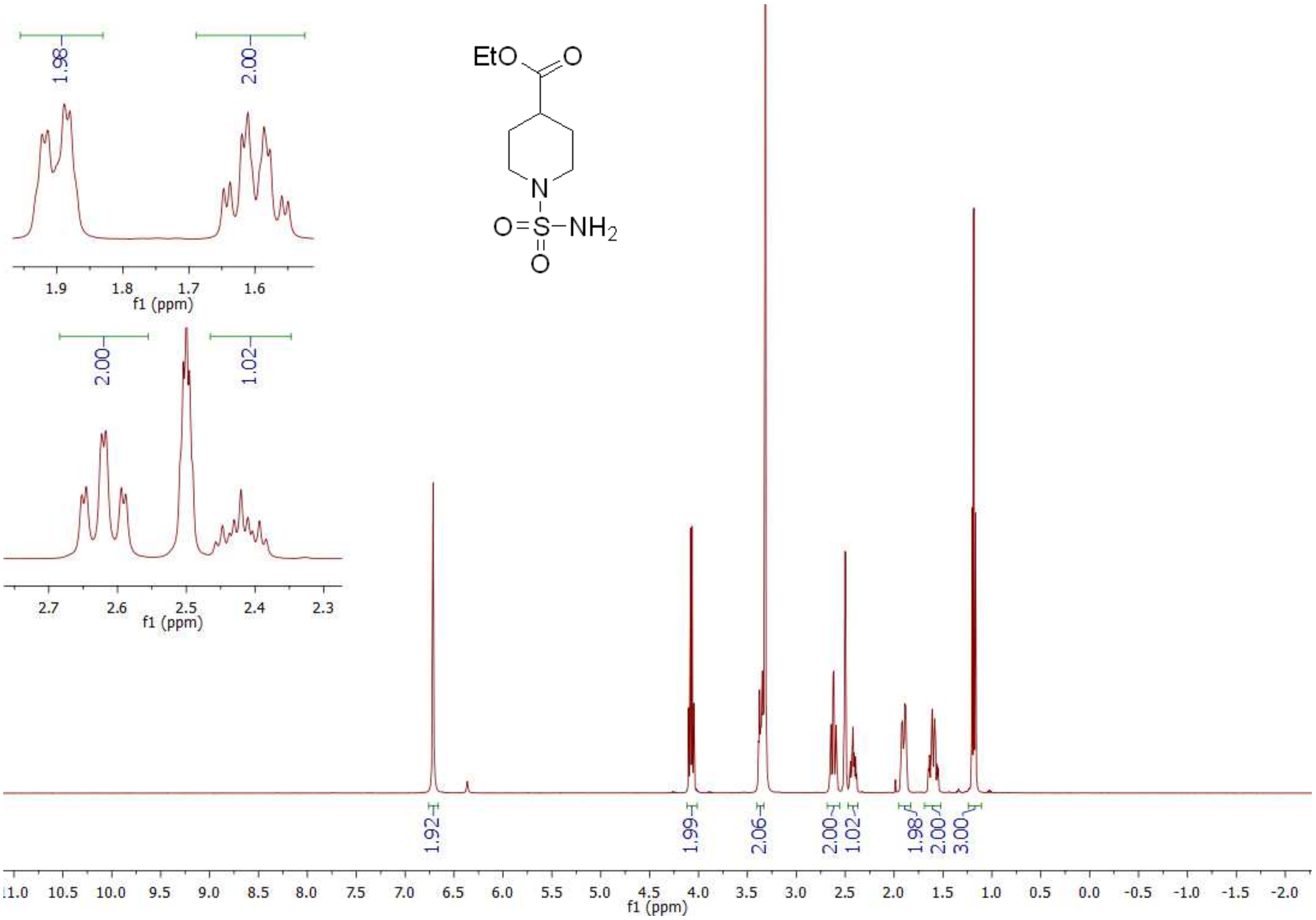

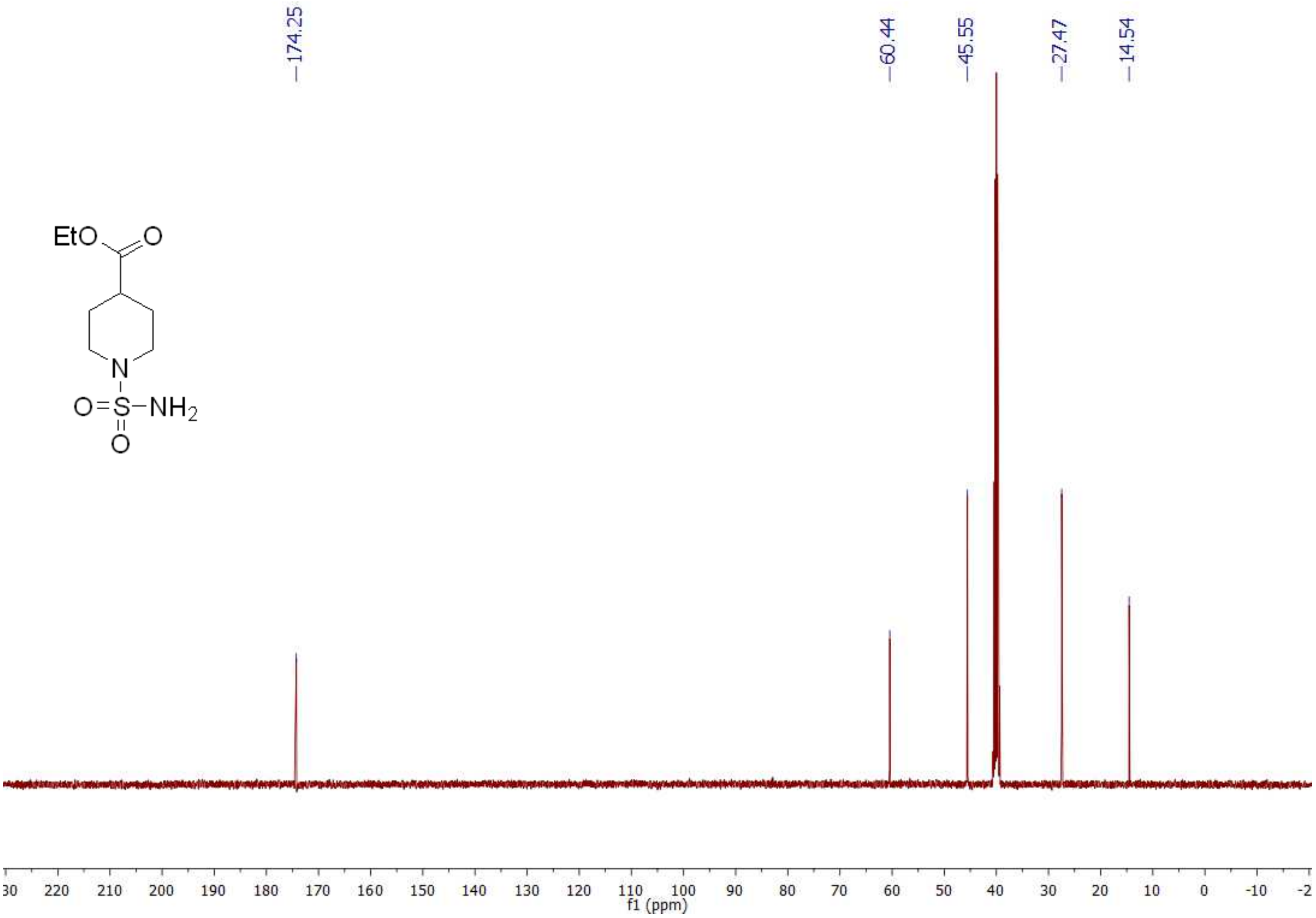

^{1}H NMR and ^{13}C (DEPT) spectra of compound **2n**

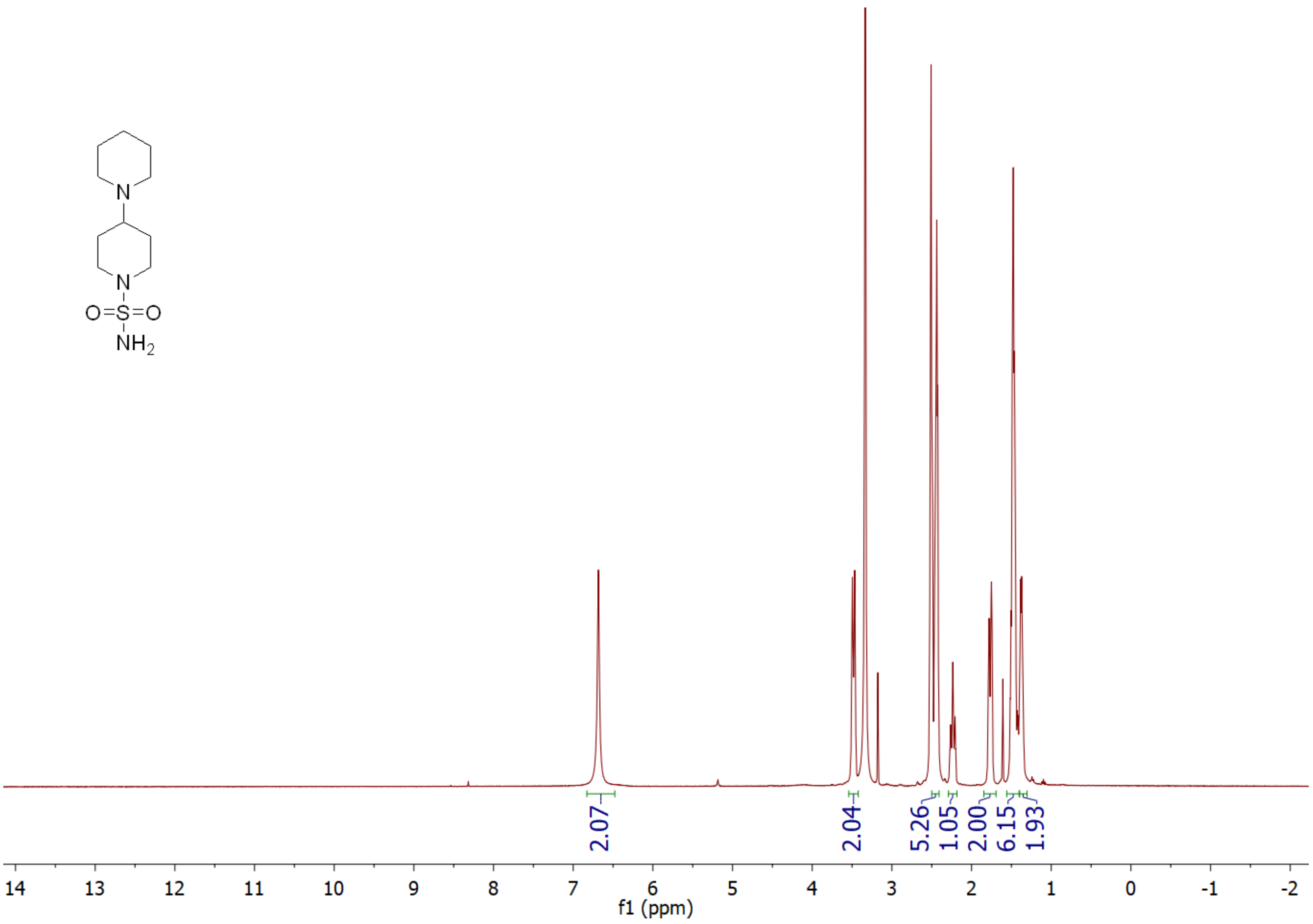

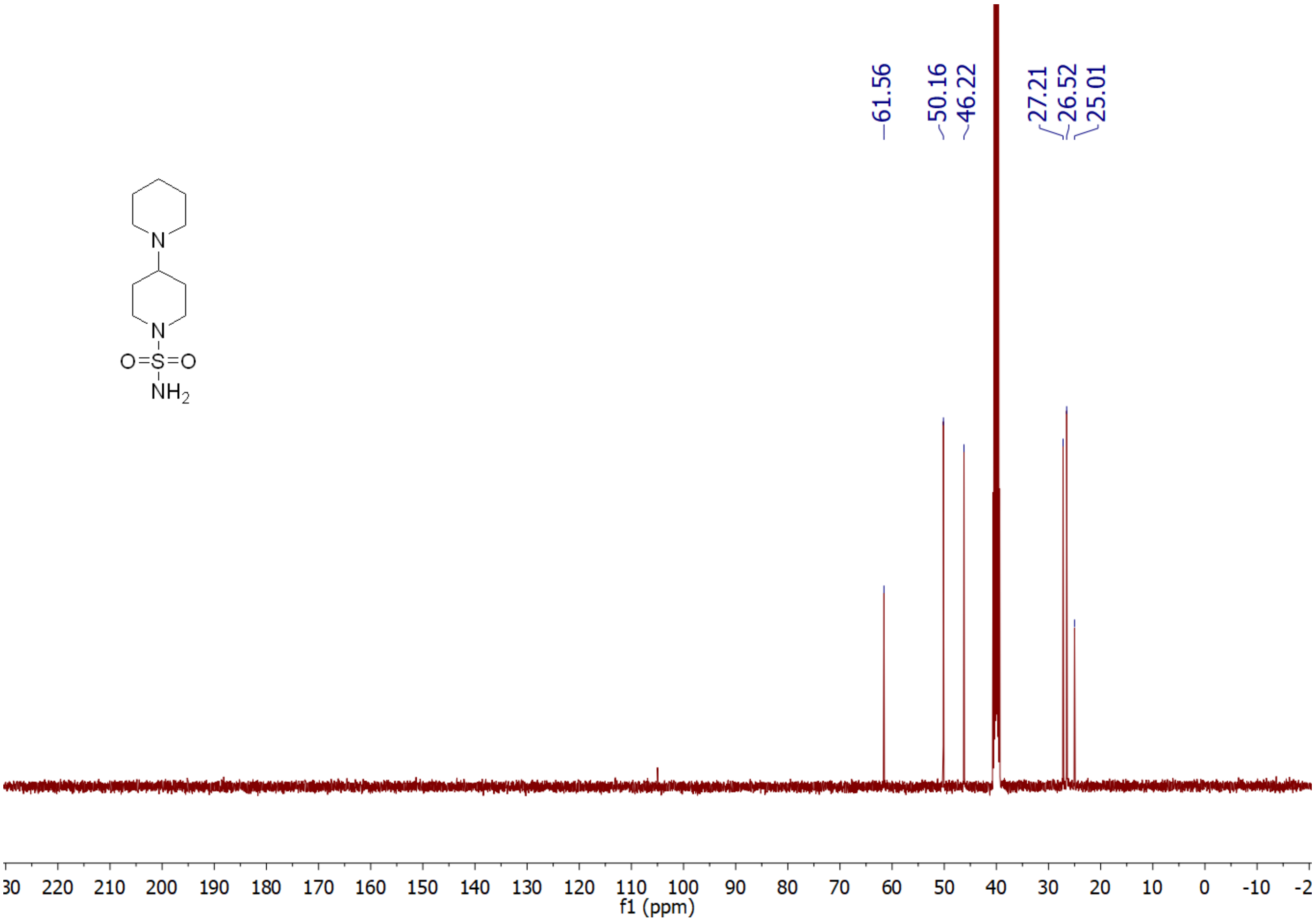

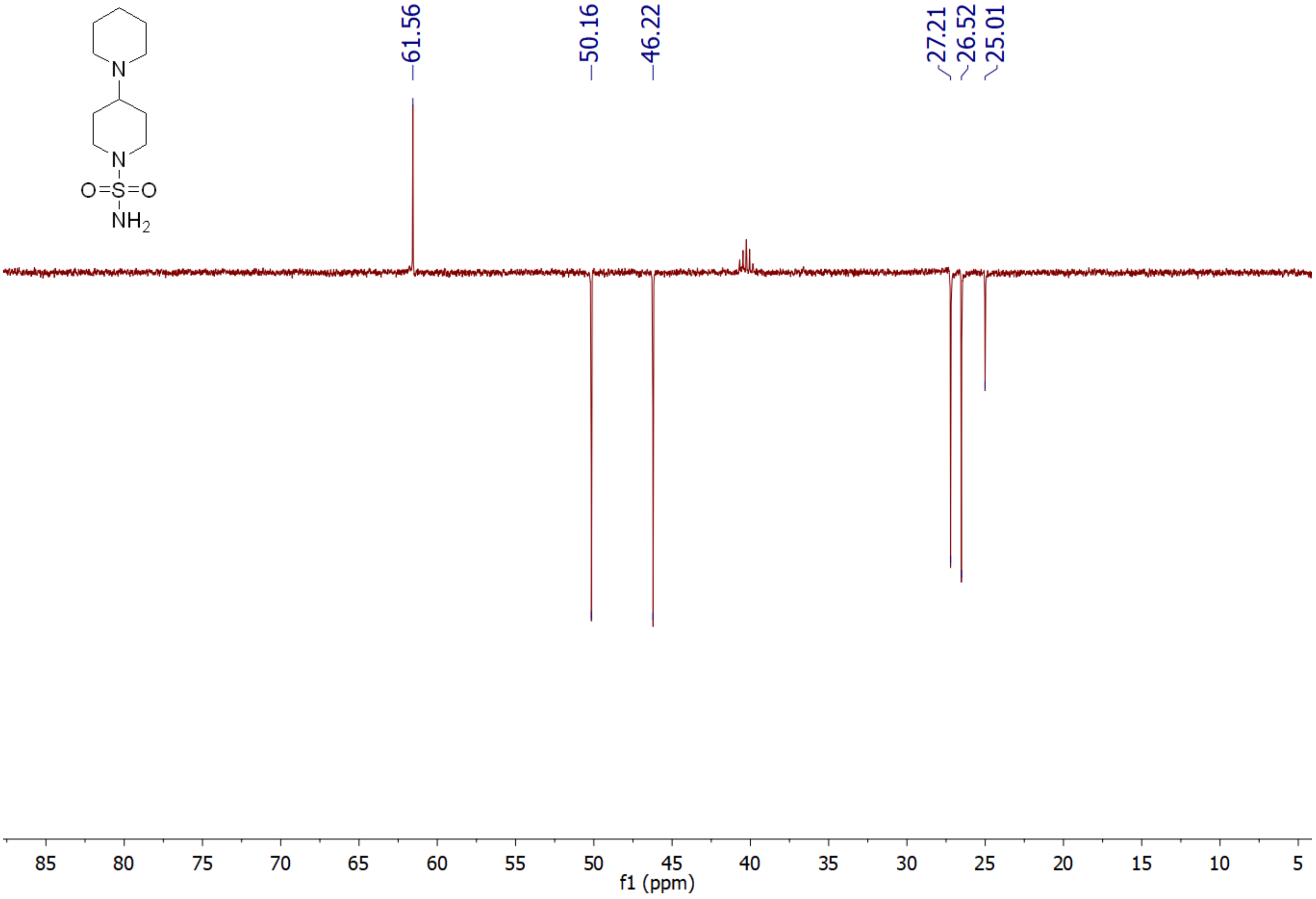

O=S=O
NH2
N
61.56
50.16
46.22
27.21
26.52
25.01
85
80
75
70
65
60
55
50
45
40
35
30
25
20
15
10
5
f1 (ppm)

^{1}H NMR and ^{13}C spectra of compound **2o**

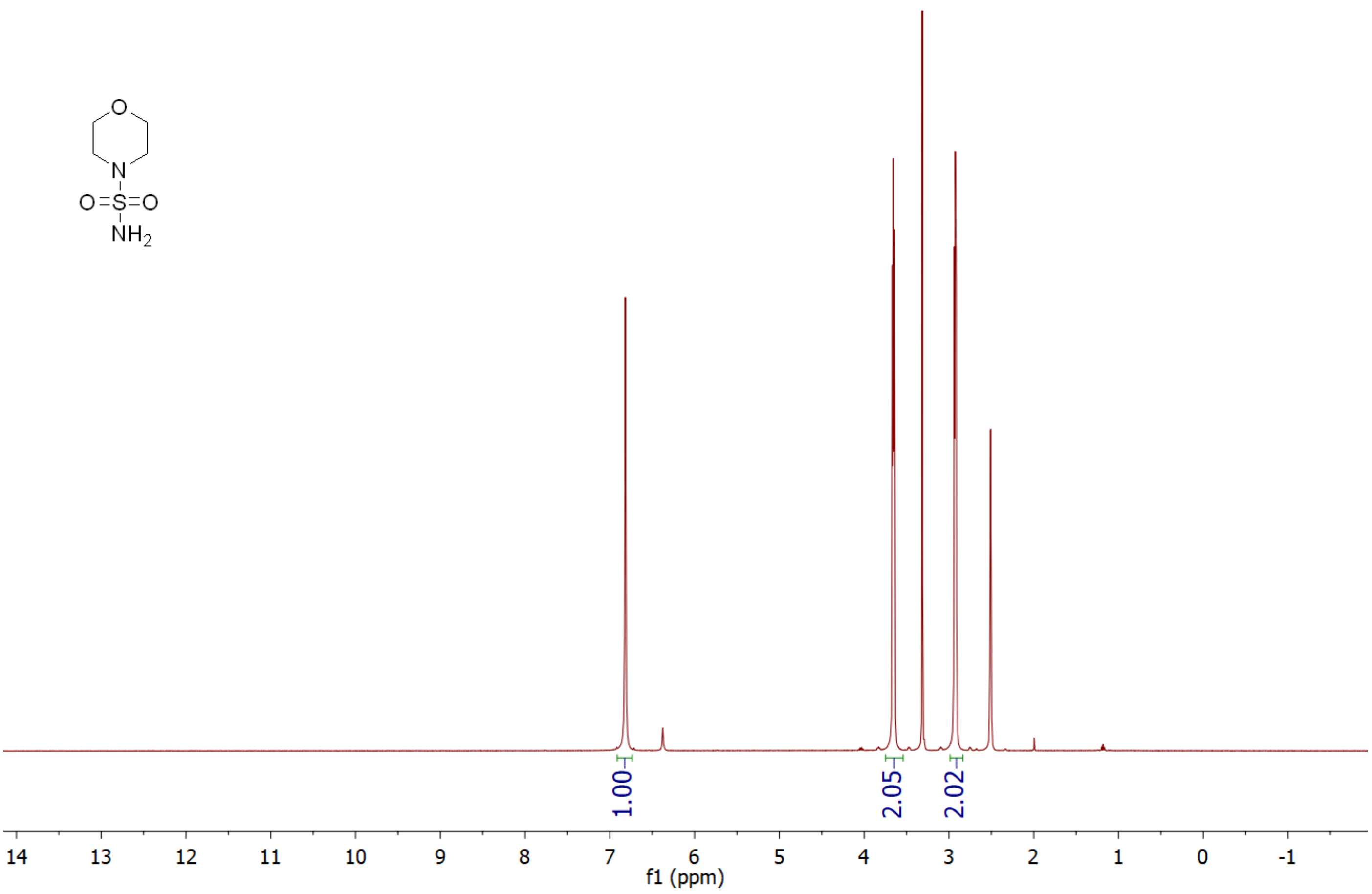

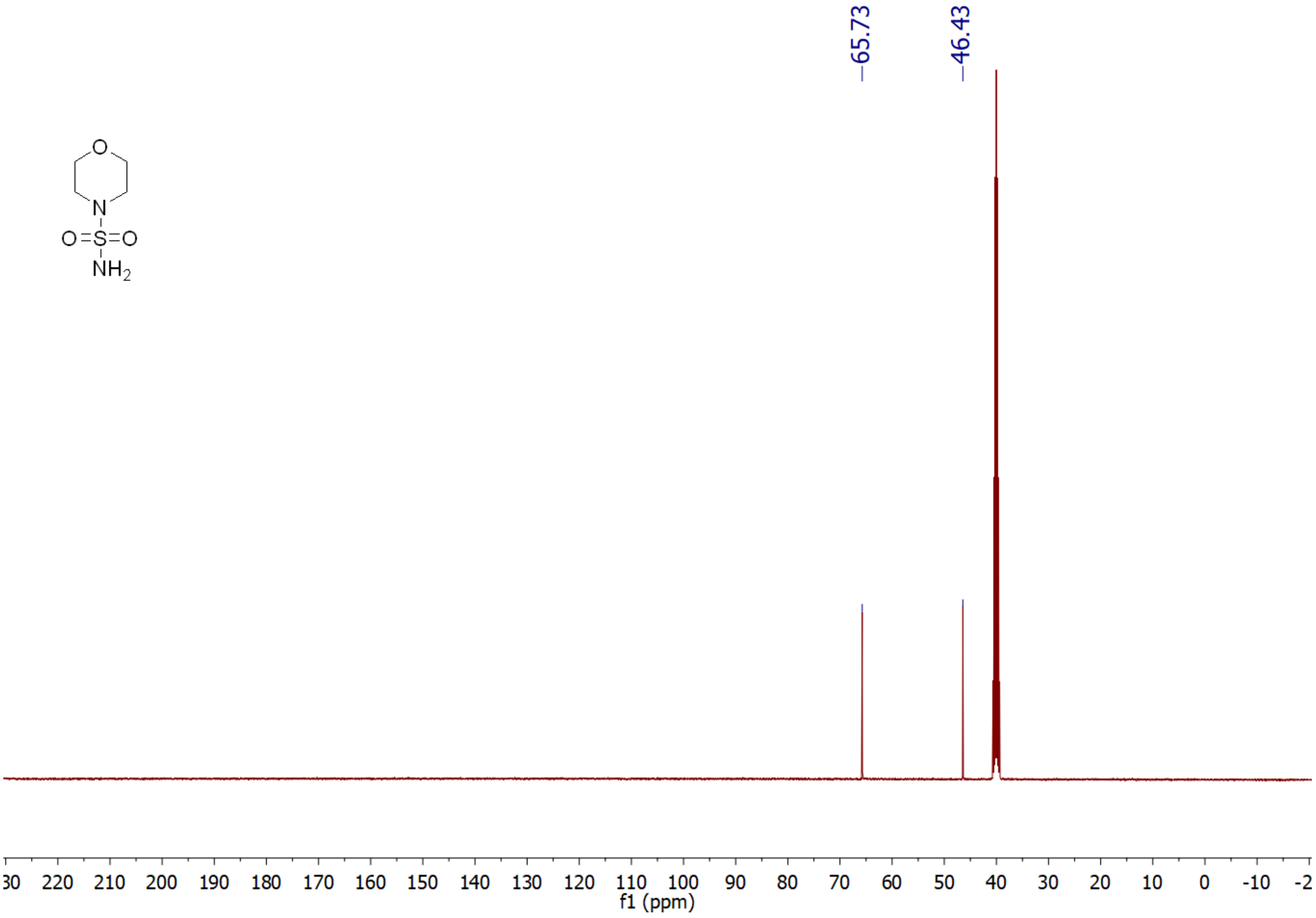

^{1}H NMR and ^{13}C (DEPT) spectra of compound **2p**

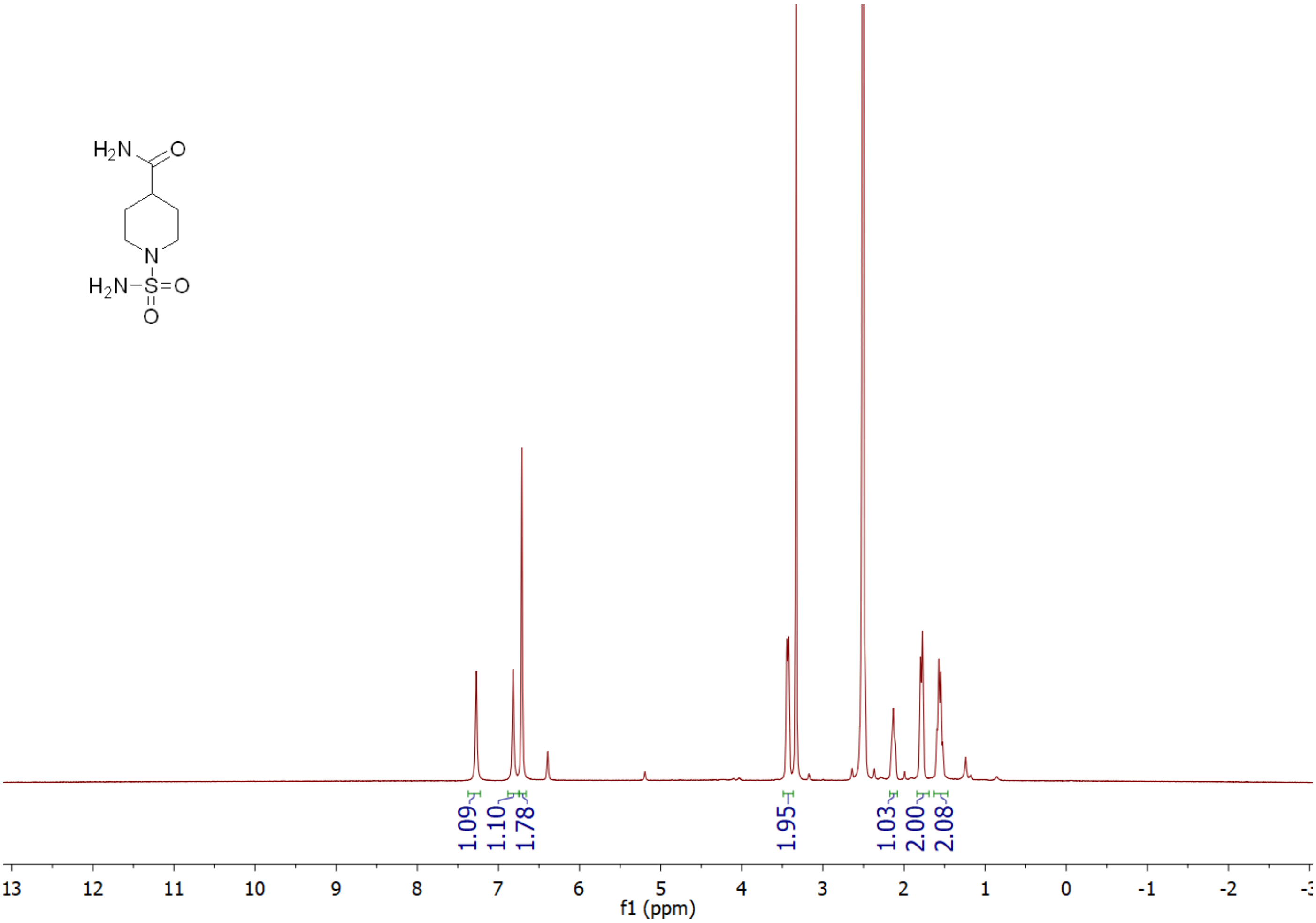

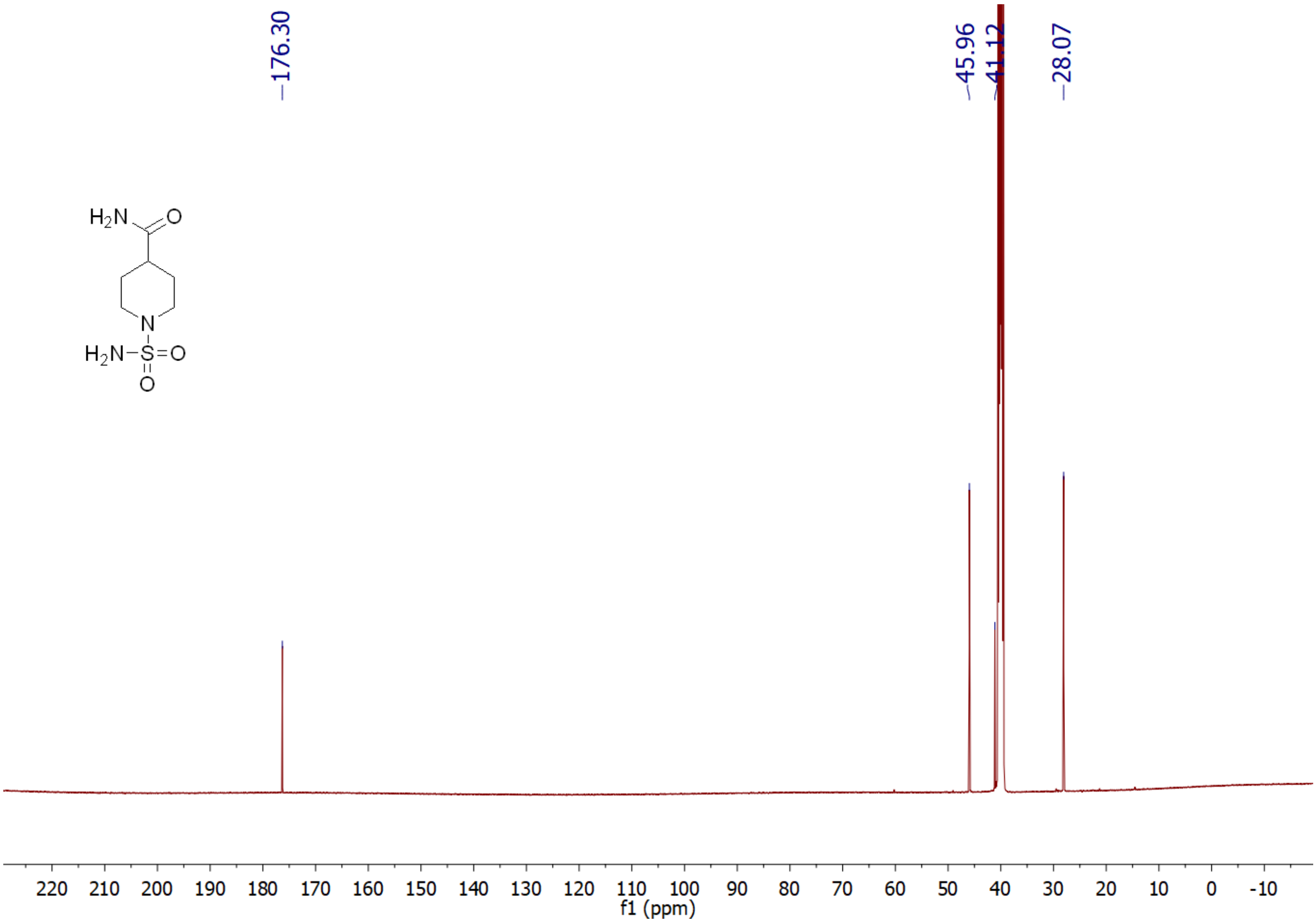

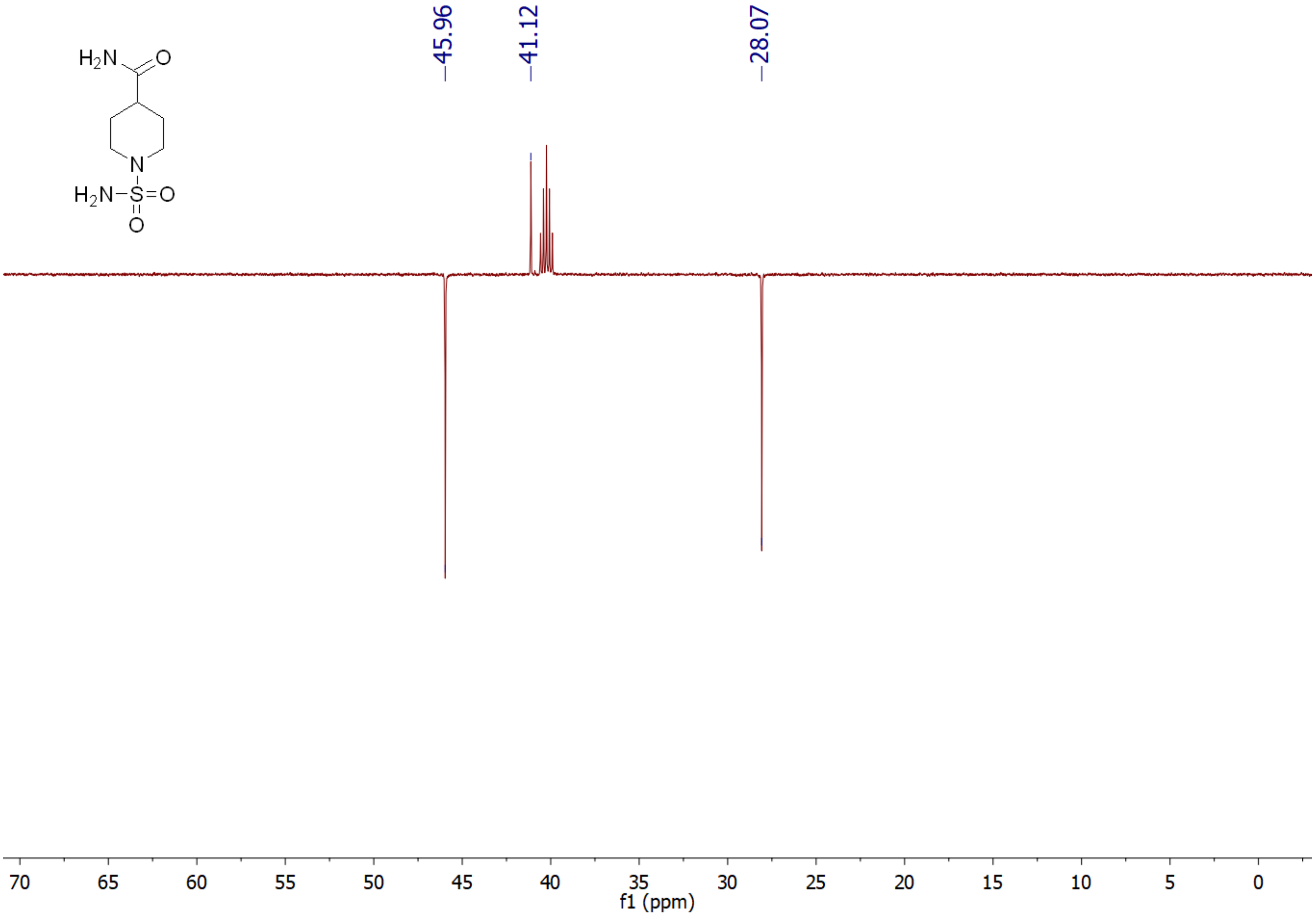

H2N
O
N
H2N
S
O
O
45.96
41.12
28.07
70
65
60
55
50
45
40
35
30
25
20
15
10
5
0
f1 (ppm)

^{1}H NMR and ^{13}C (DEPT) spectra of compound **2q**

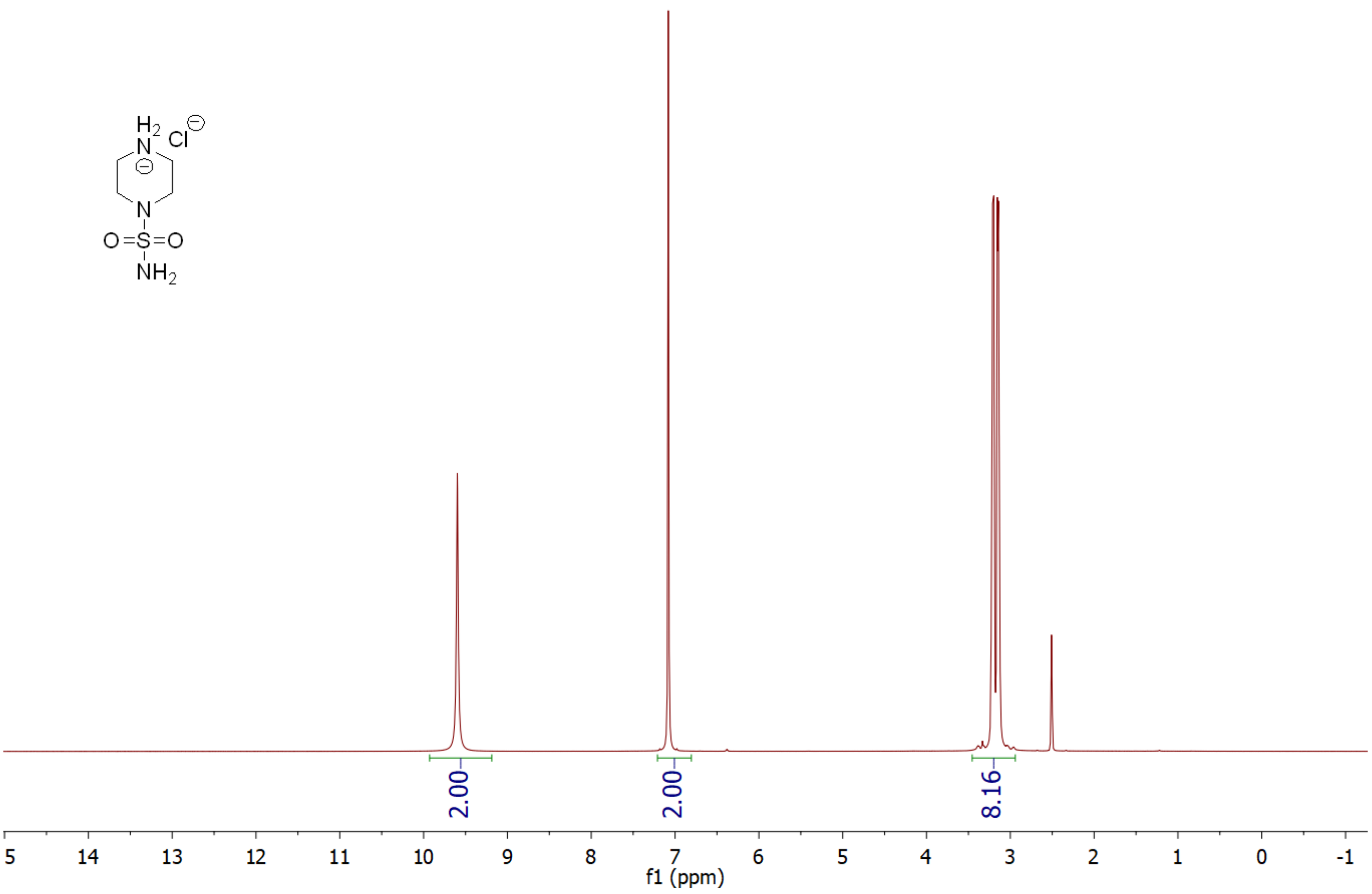

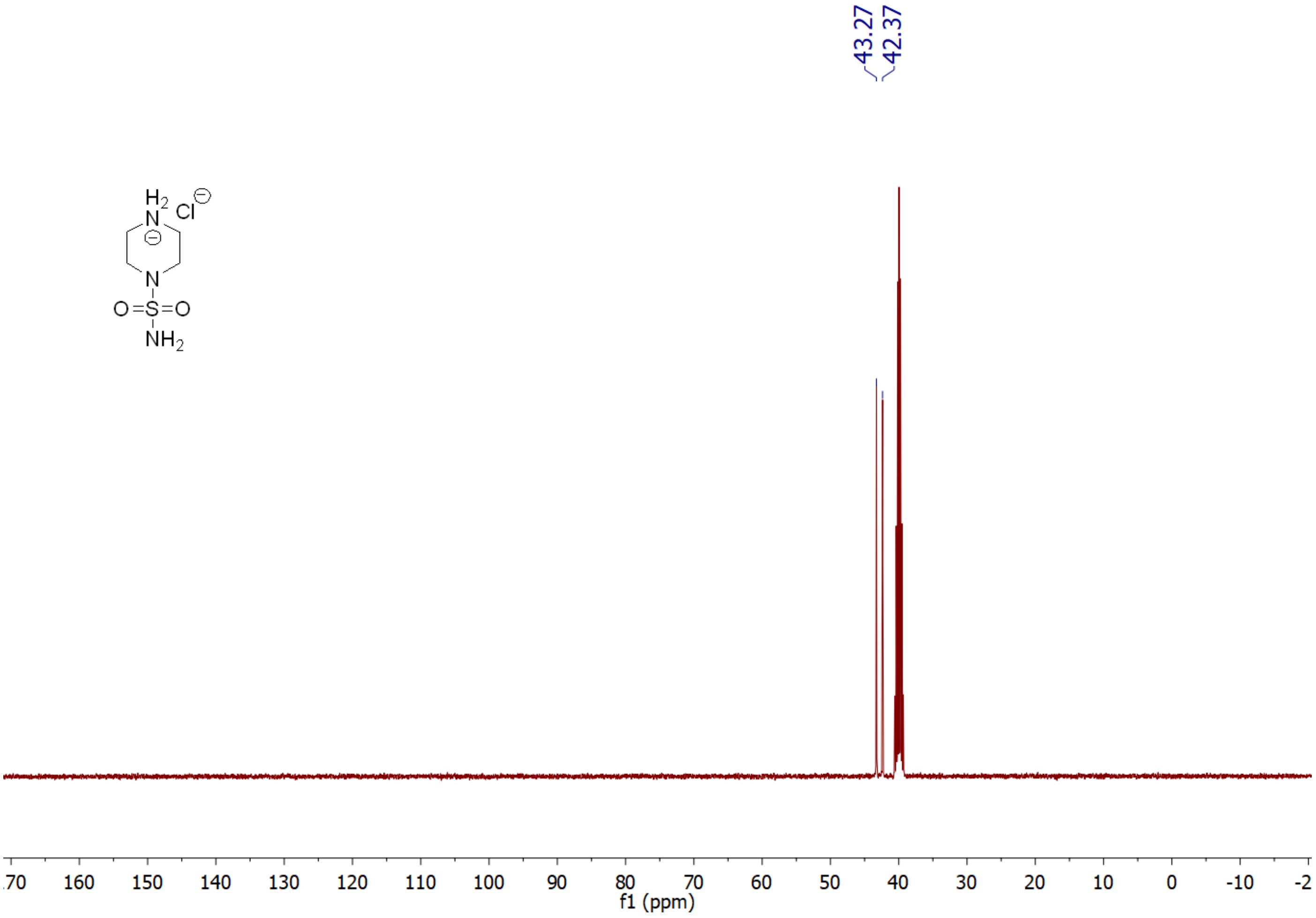

^{1}H NMR and ^{13}C spectra of compound **2r**

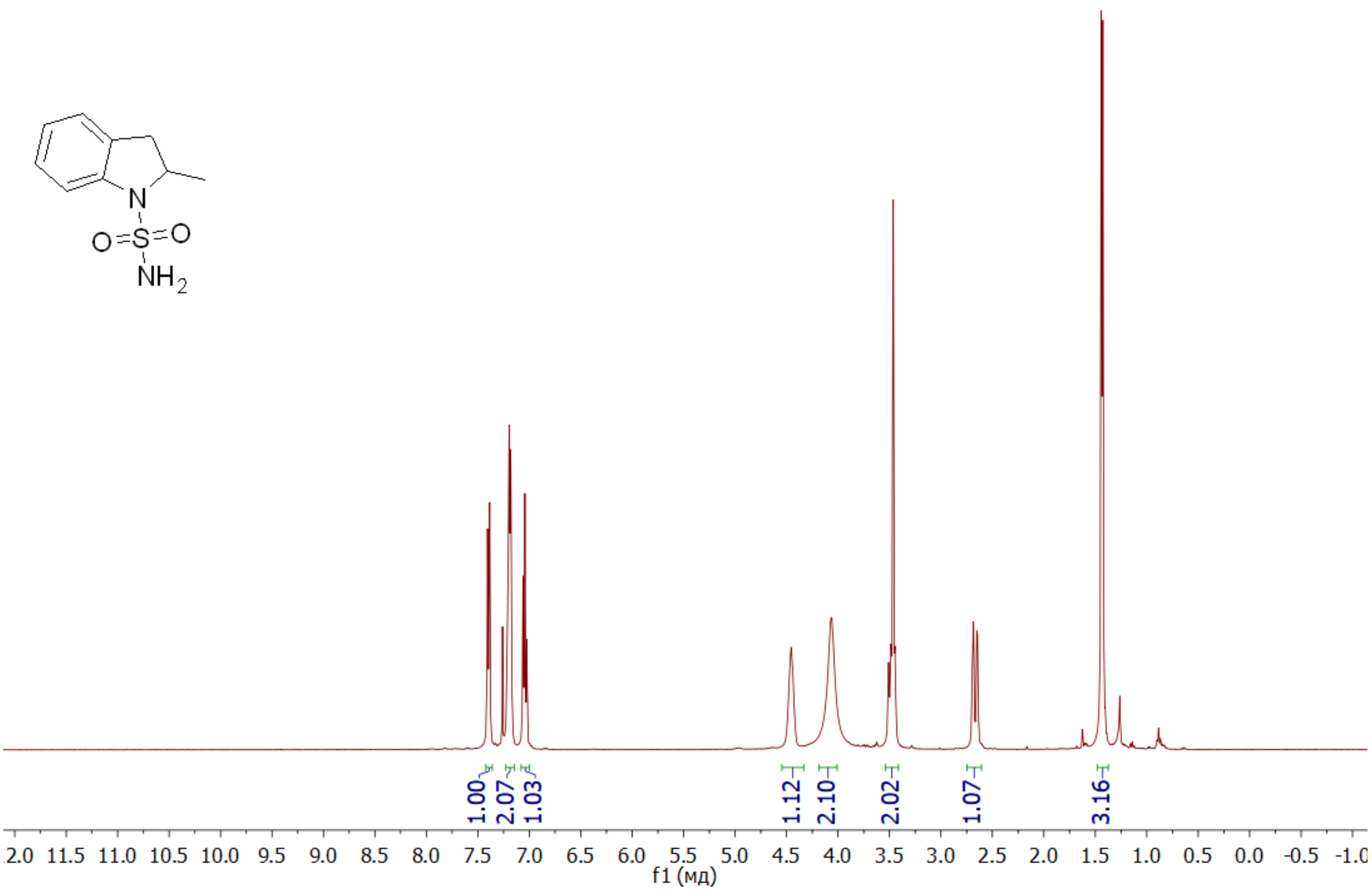

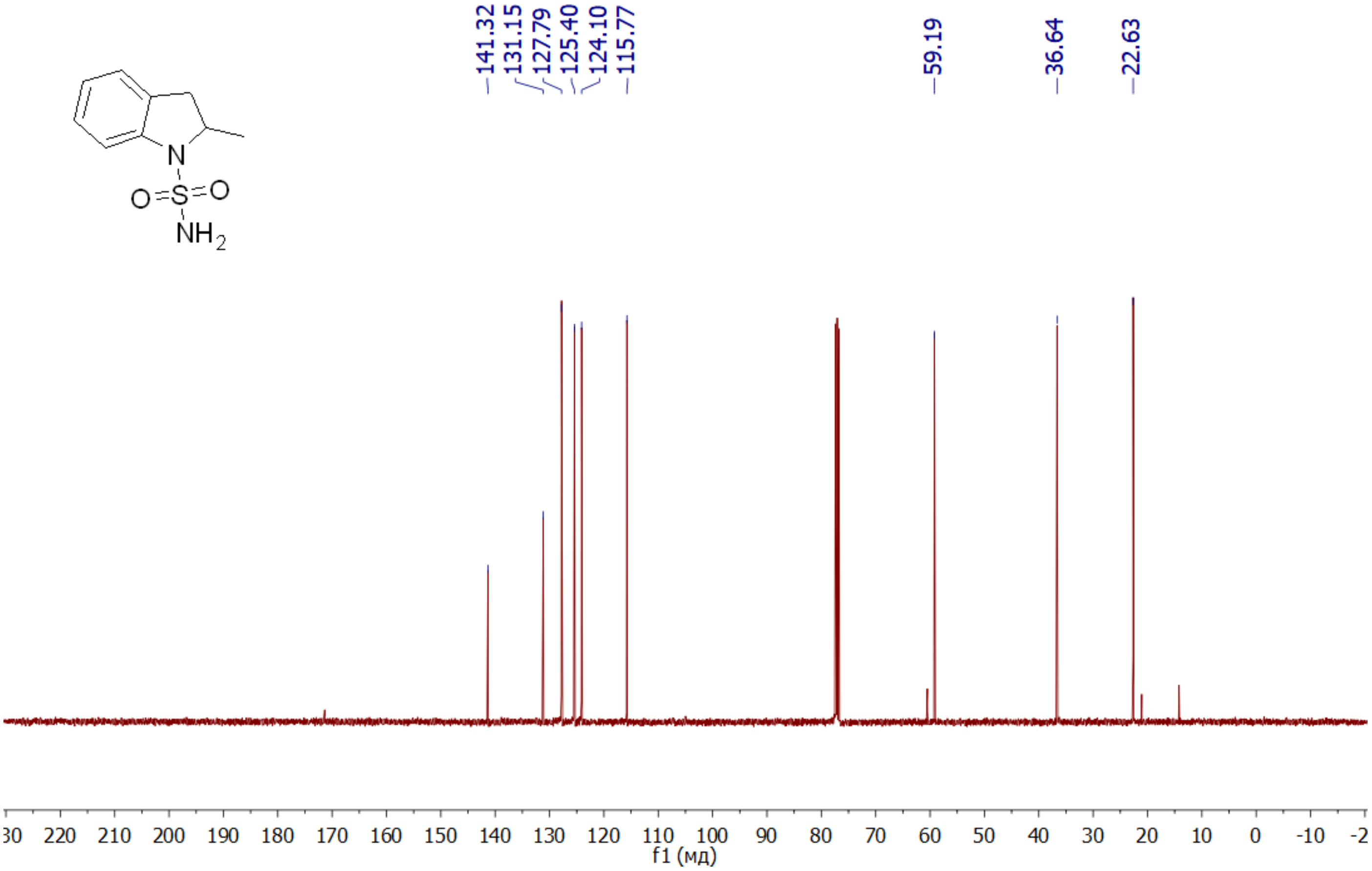

^{1}H NMR and ^{13}C spectra of compound **2s**

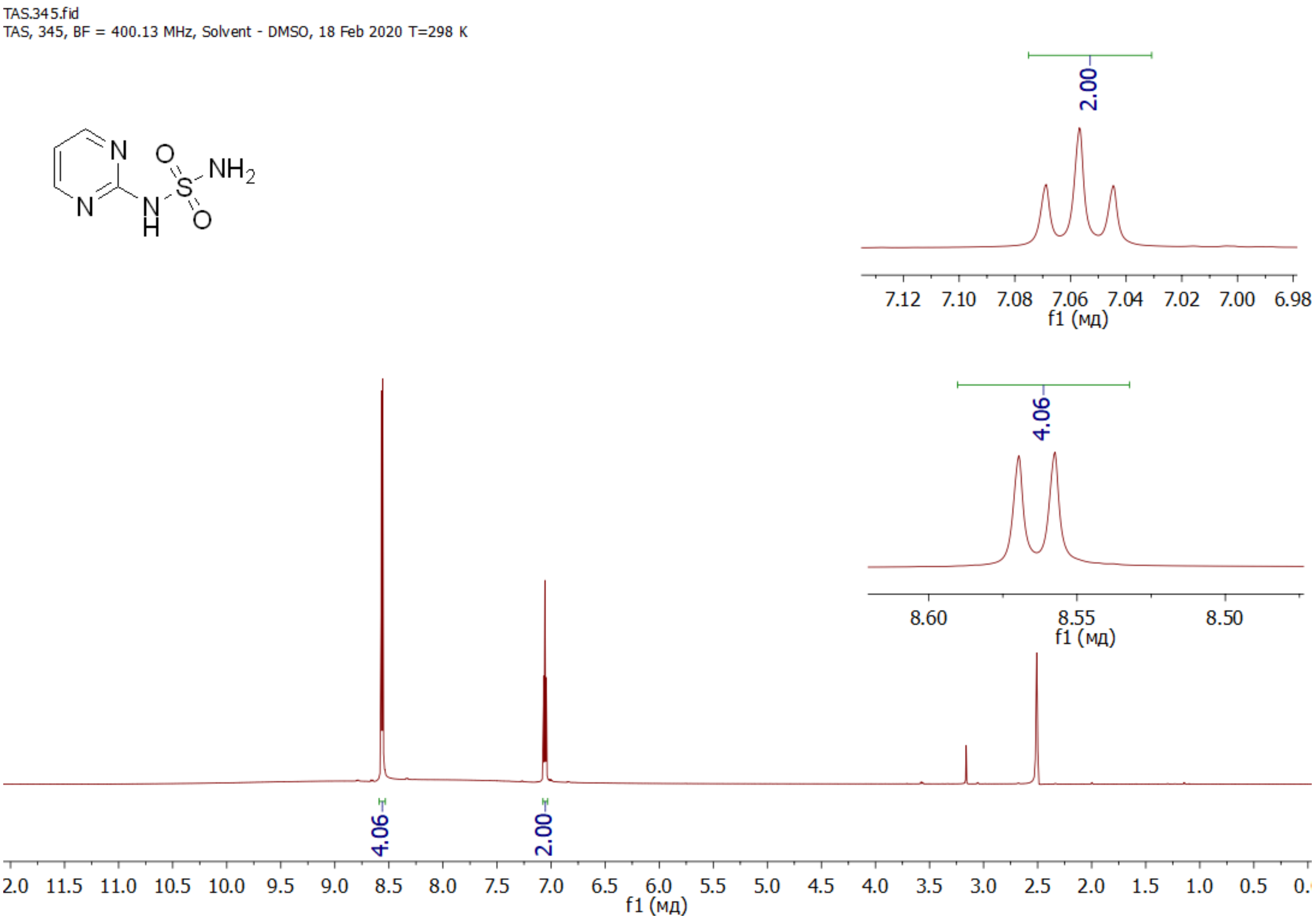

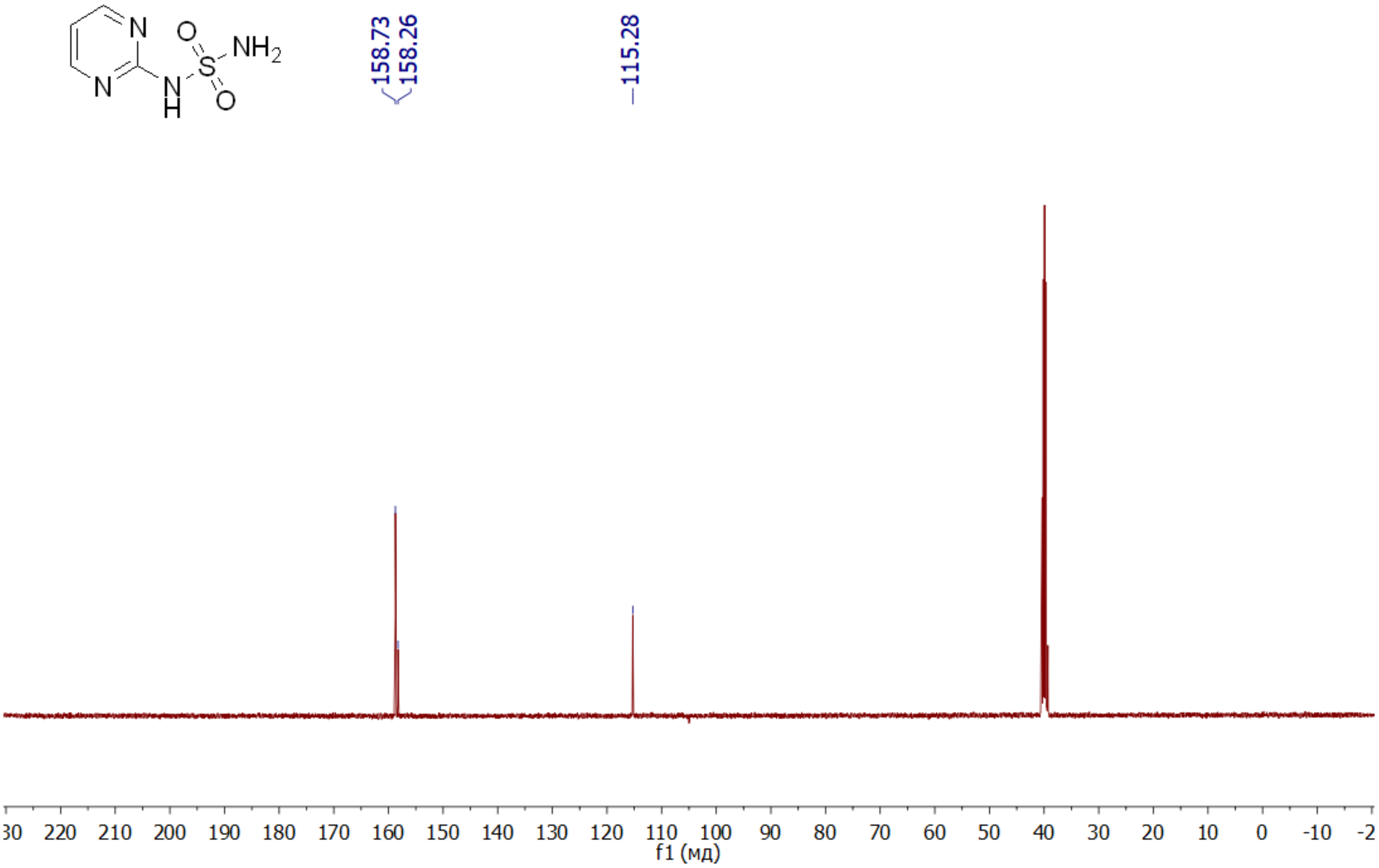

^{1}H NMR and ^{13}C (DEPT) spectra of compound **2t**

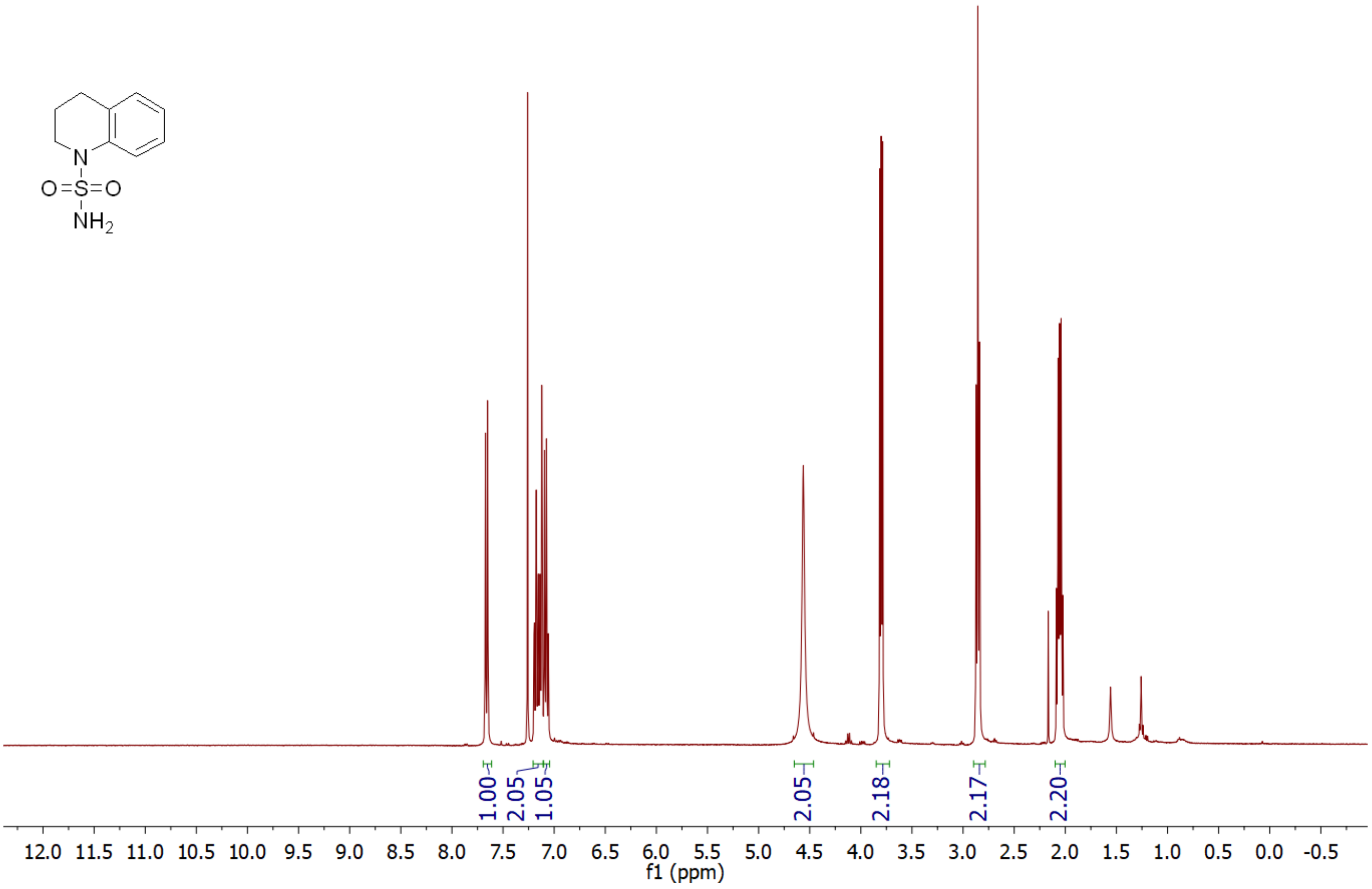

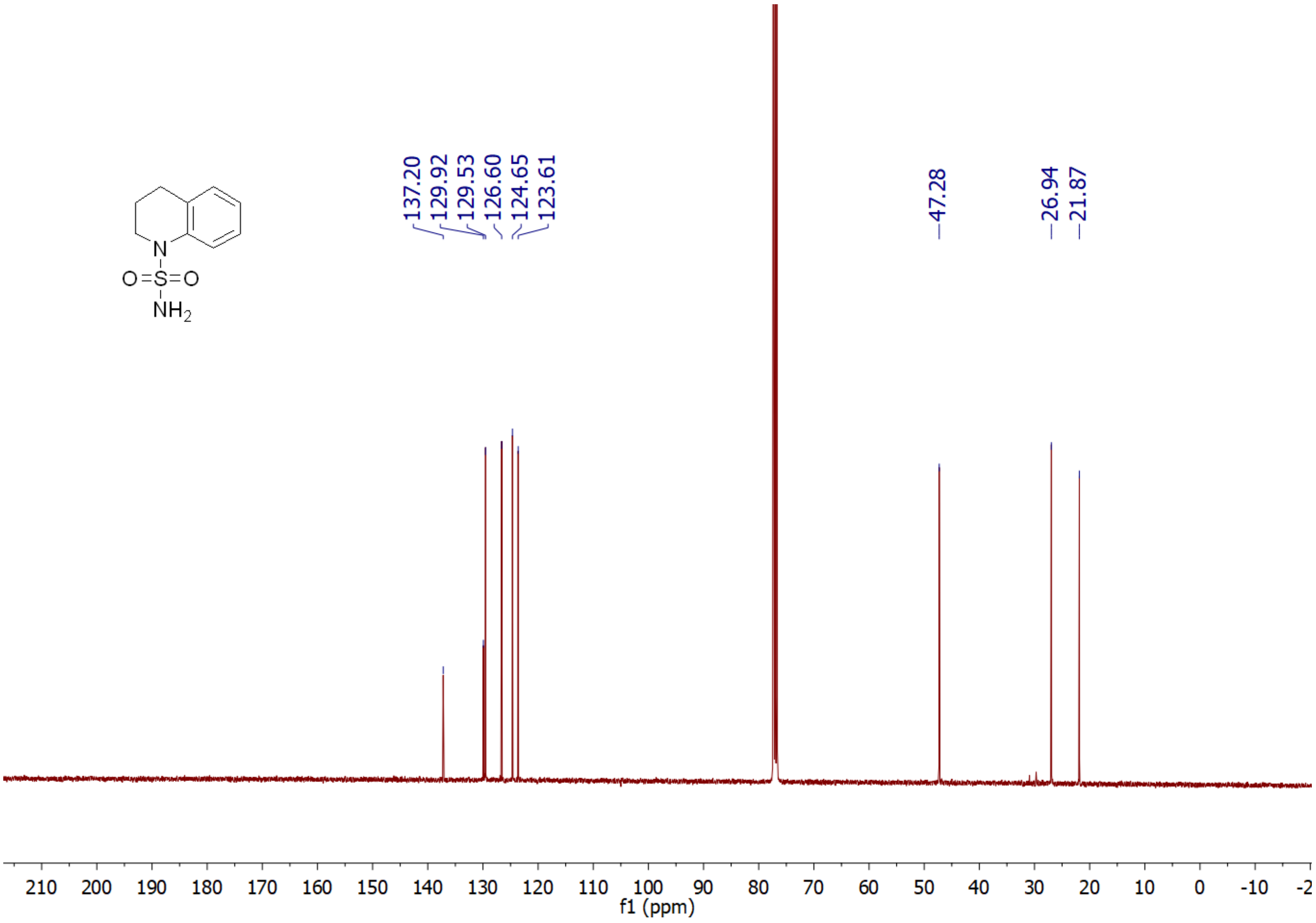

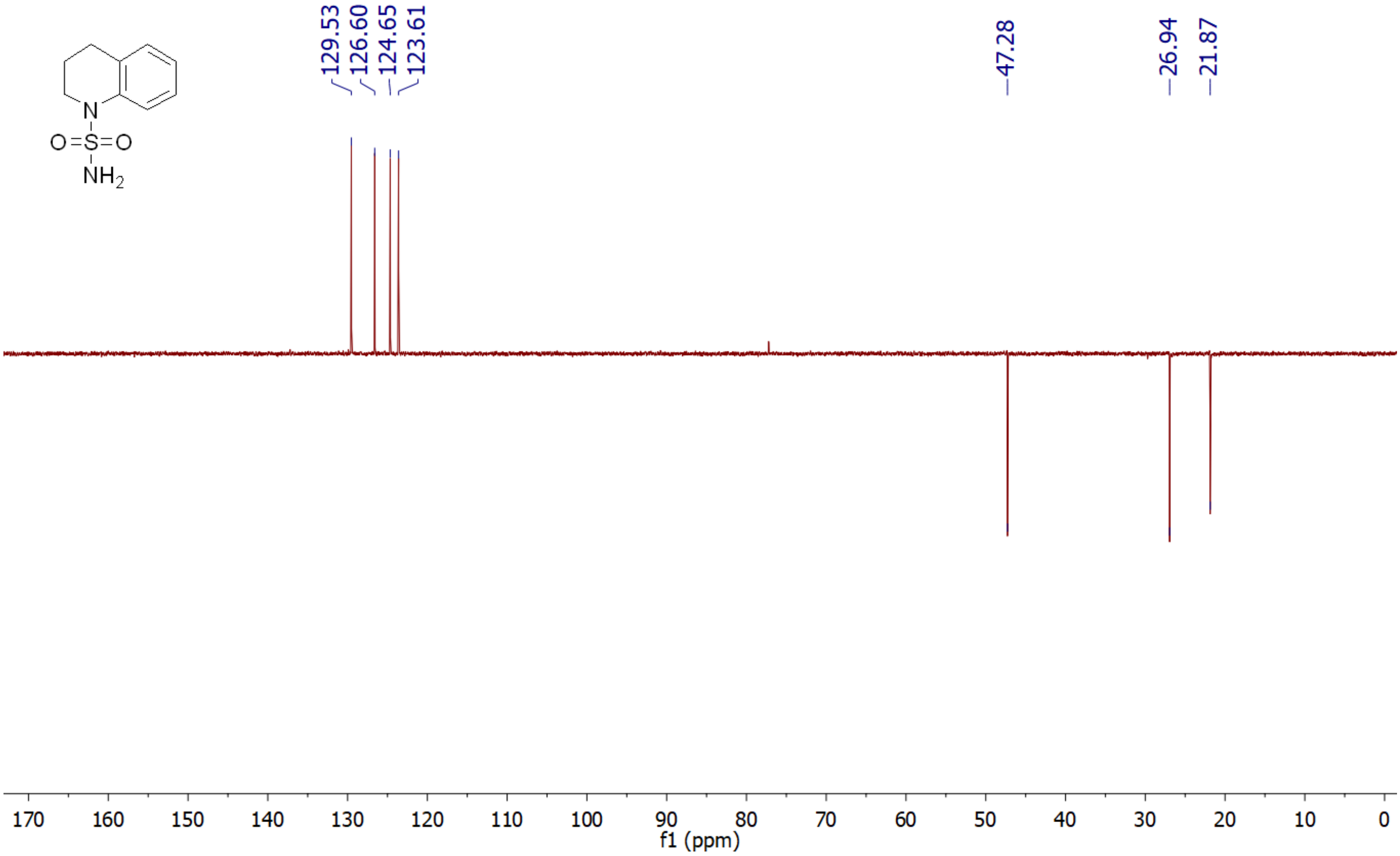
N
O=S=O
NH2
129.53
126.60
124.65
123.61
47.28
26.94
21.87
170 160 150 140 130 120 110 100 90 80 70 60 50 40 30 20 10 0
f1 (ppm)

^{1}H NMR and ^{13}C (DEPT) spectra of compound **2u**

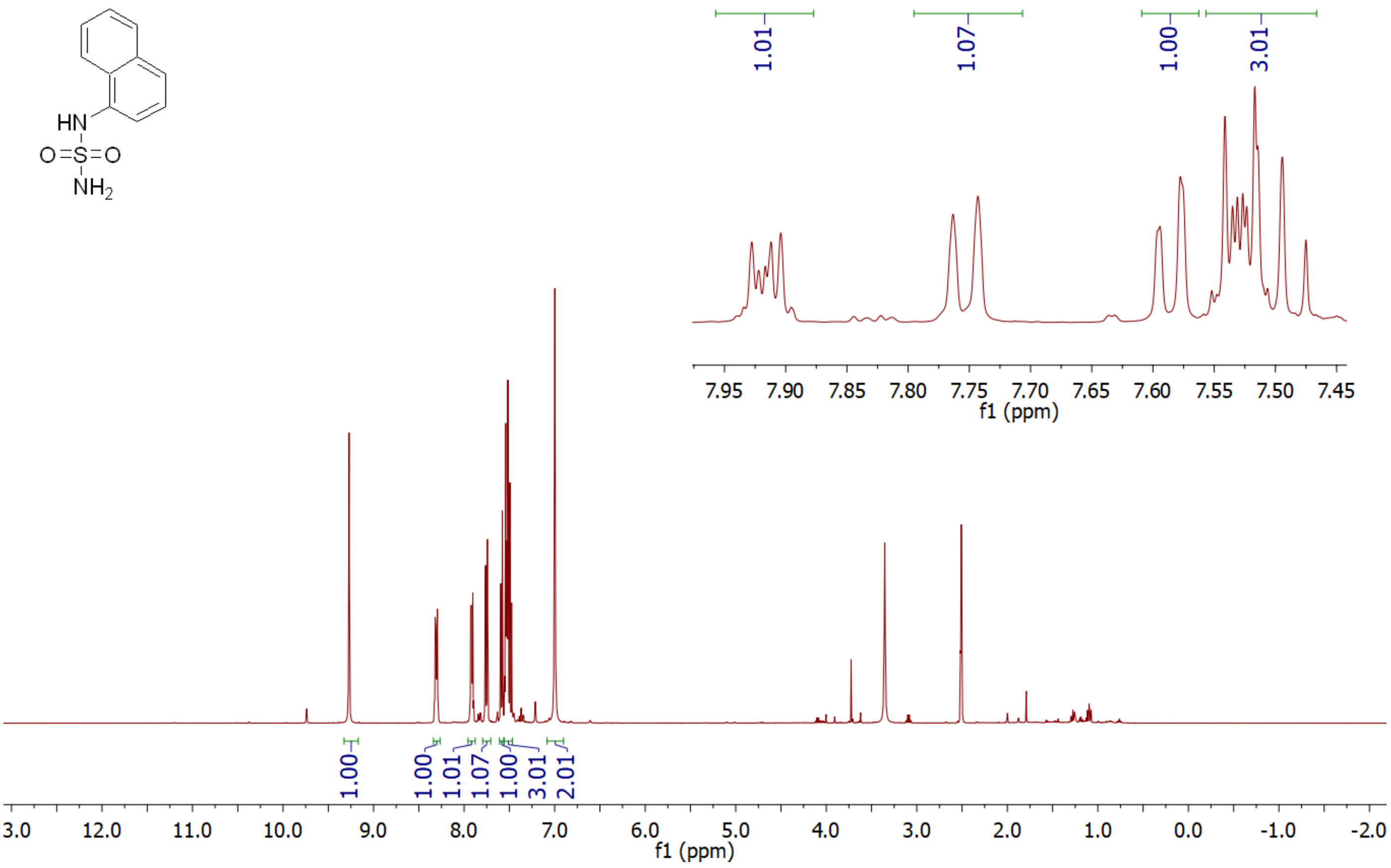

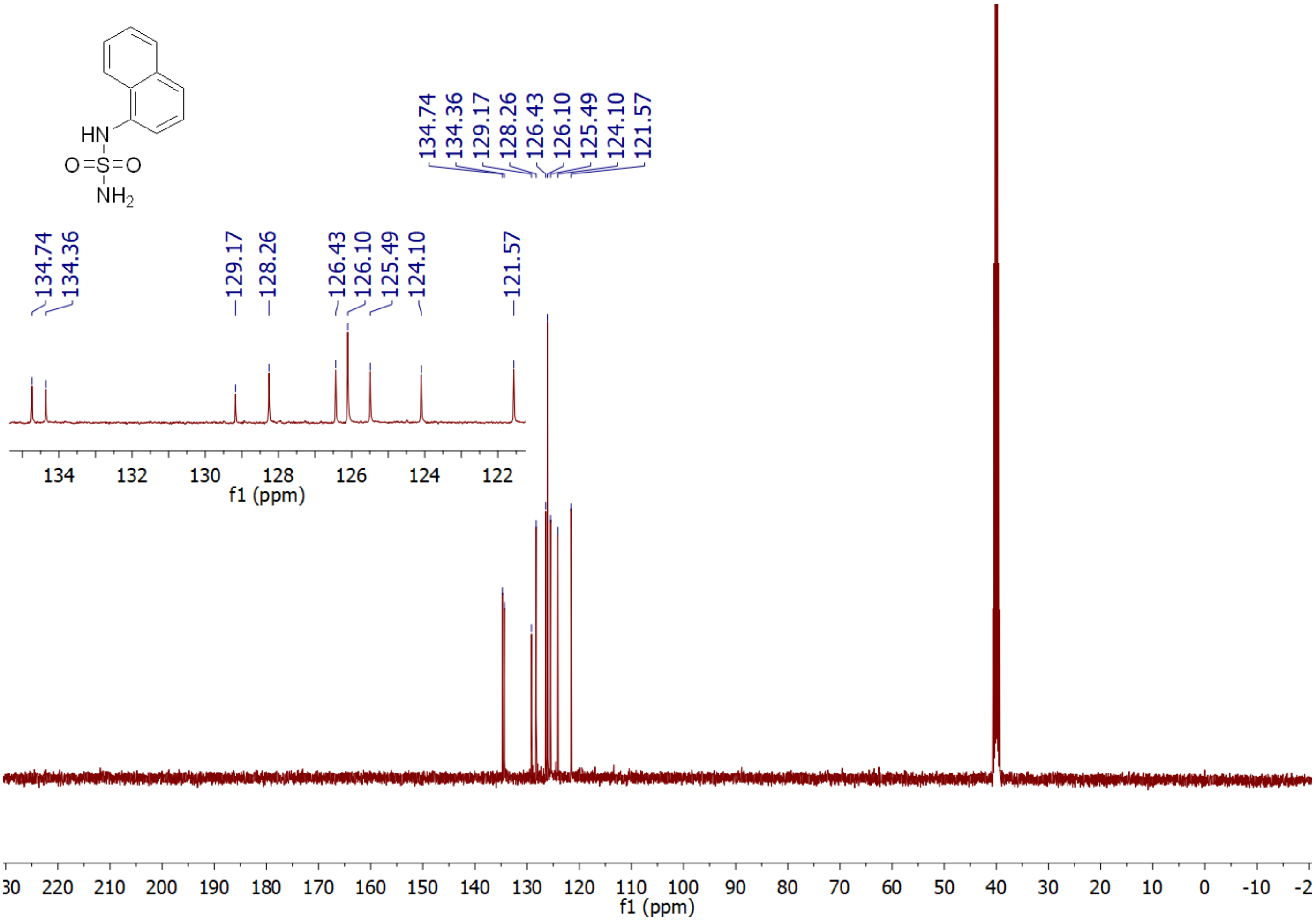

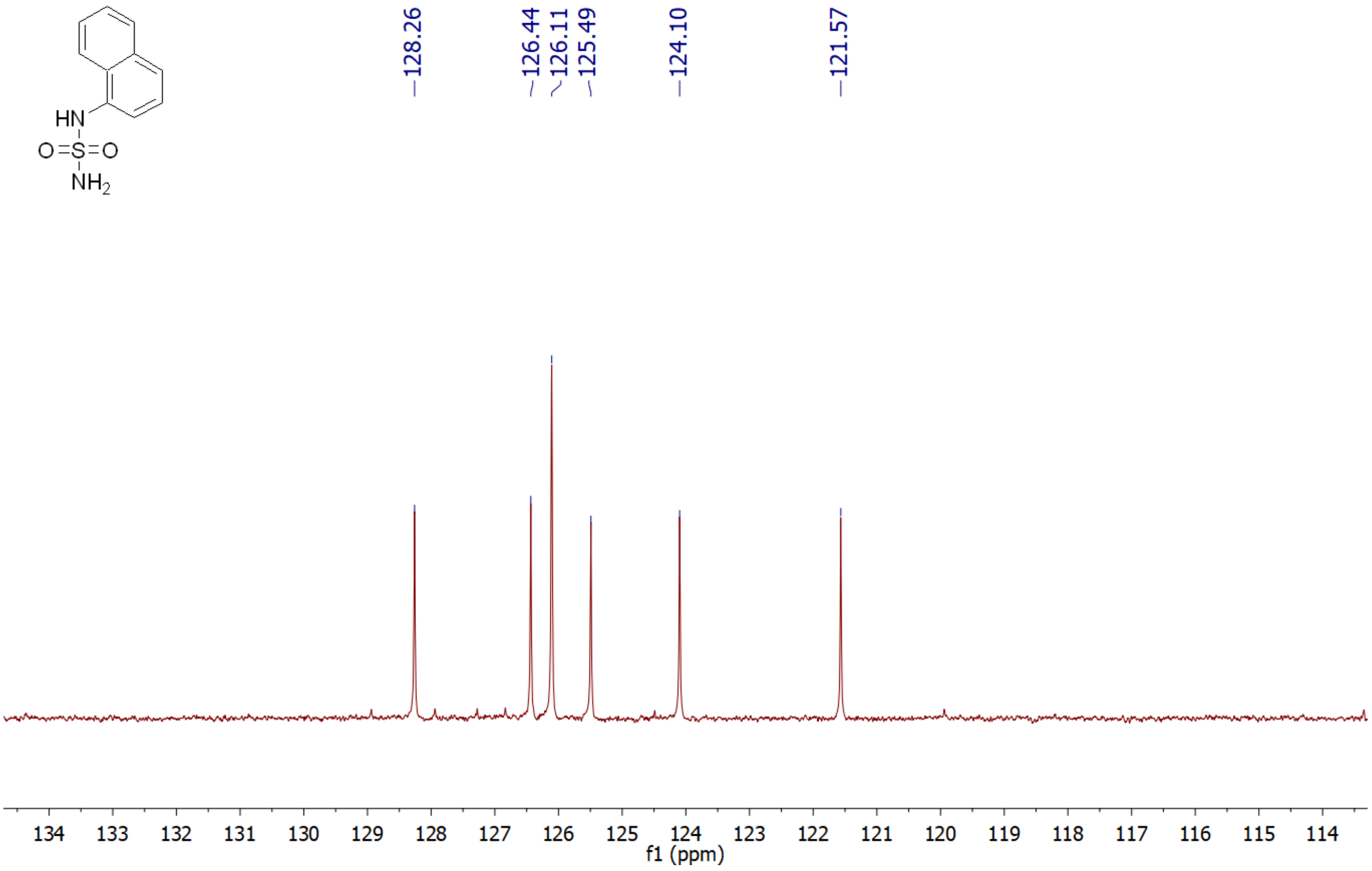
HN
O=S=O
NH2
128.26
126.44
126.11
125.49
124.10
121.57
134 133 132 131 130 129 128 127 126 125 124 123 122 121 120 119 118 117 116 115 114
f1 (ppm)

^1H NMR and ^{13}C (DEPT) spectra of compound **2v**

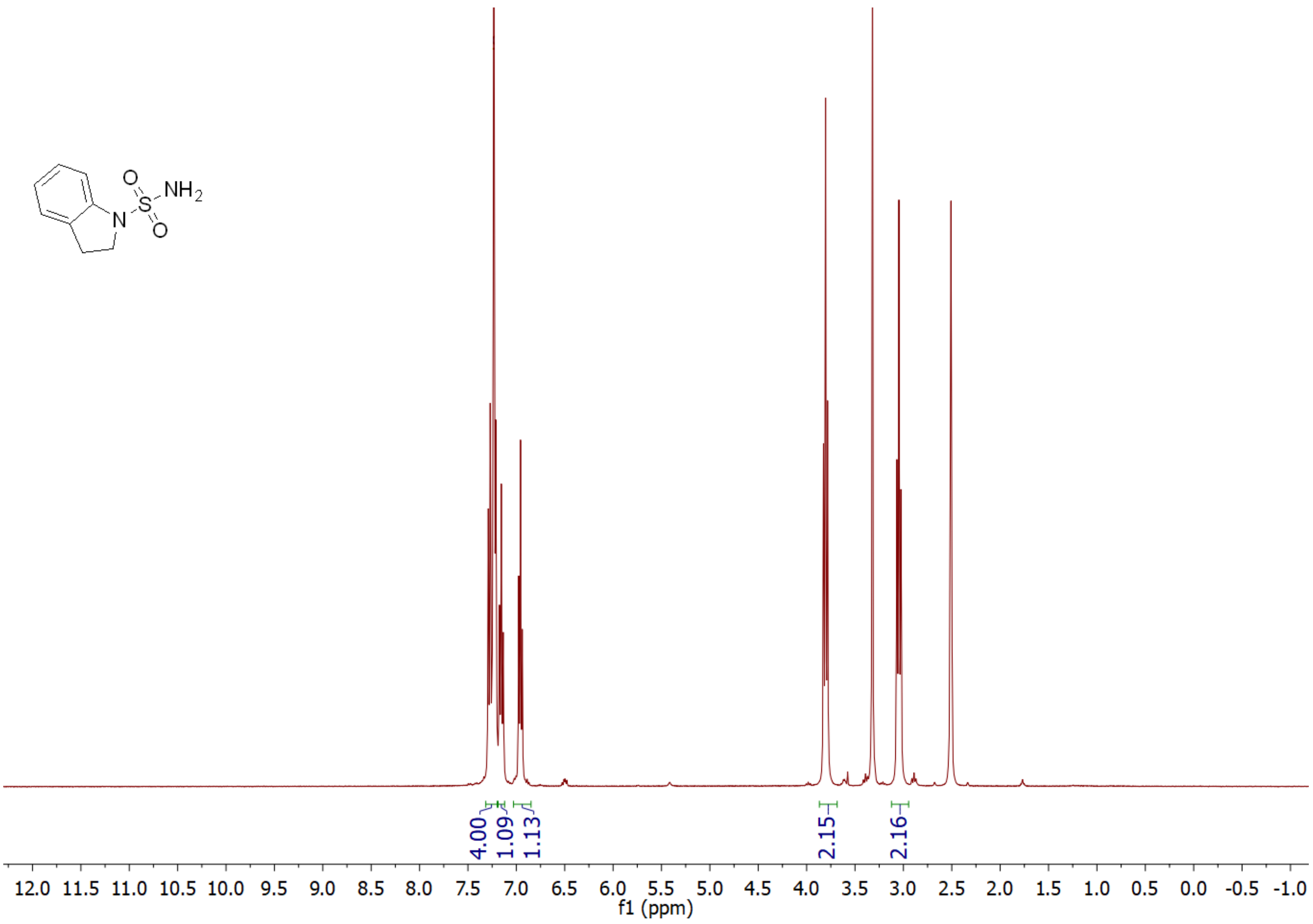

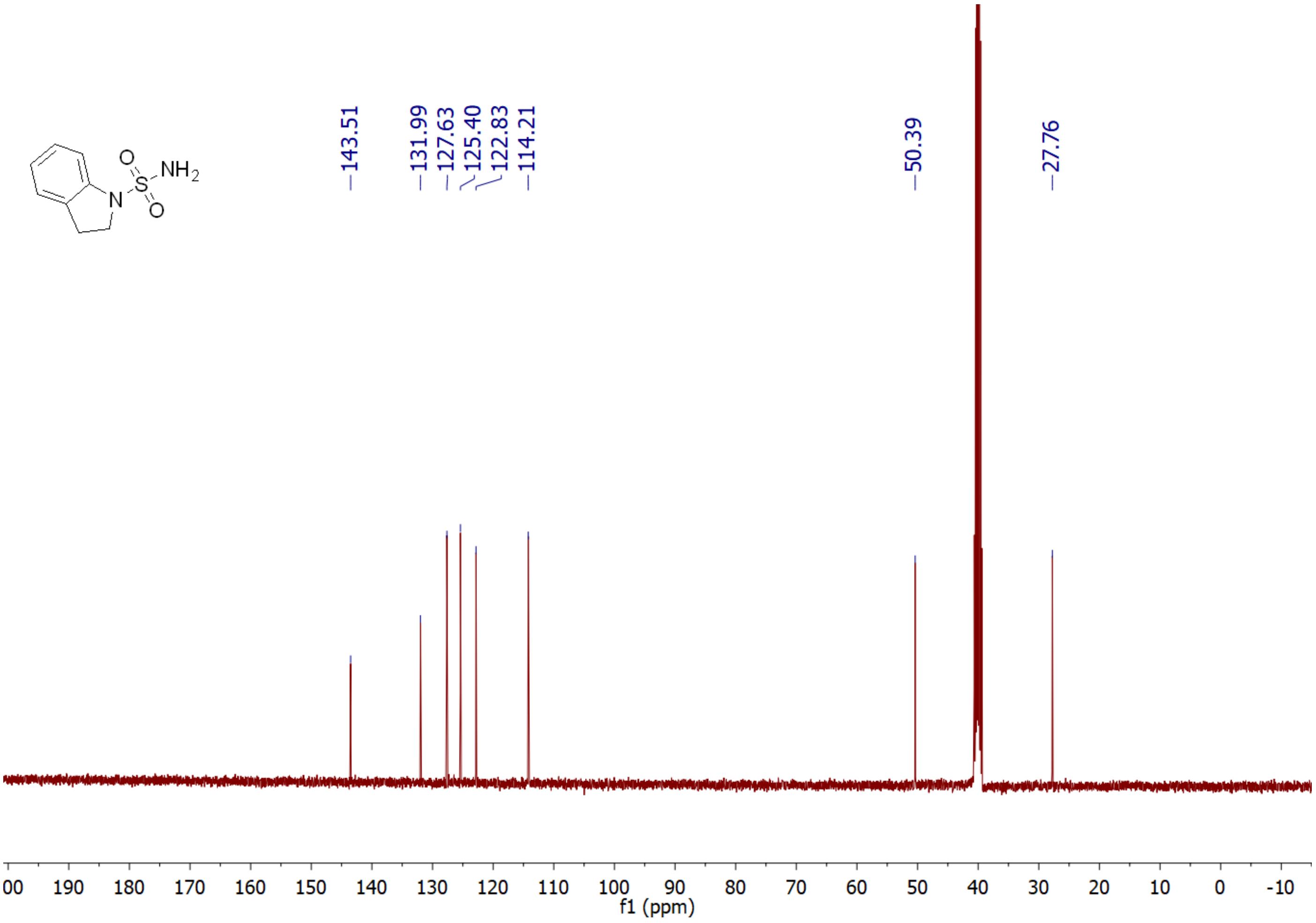

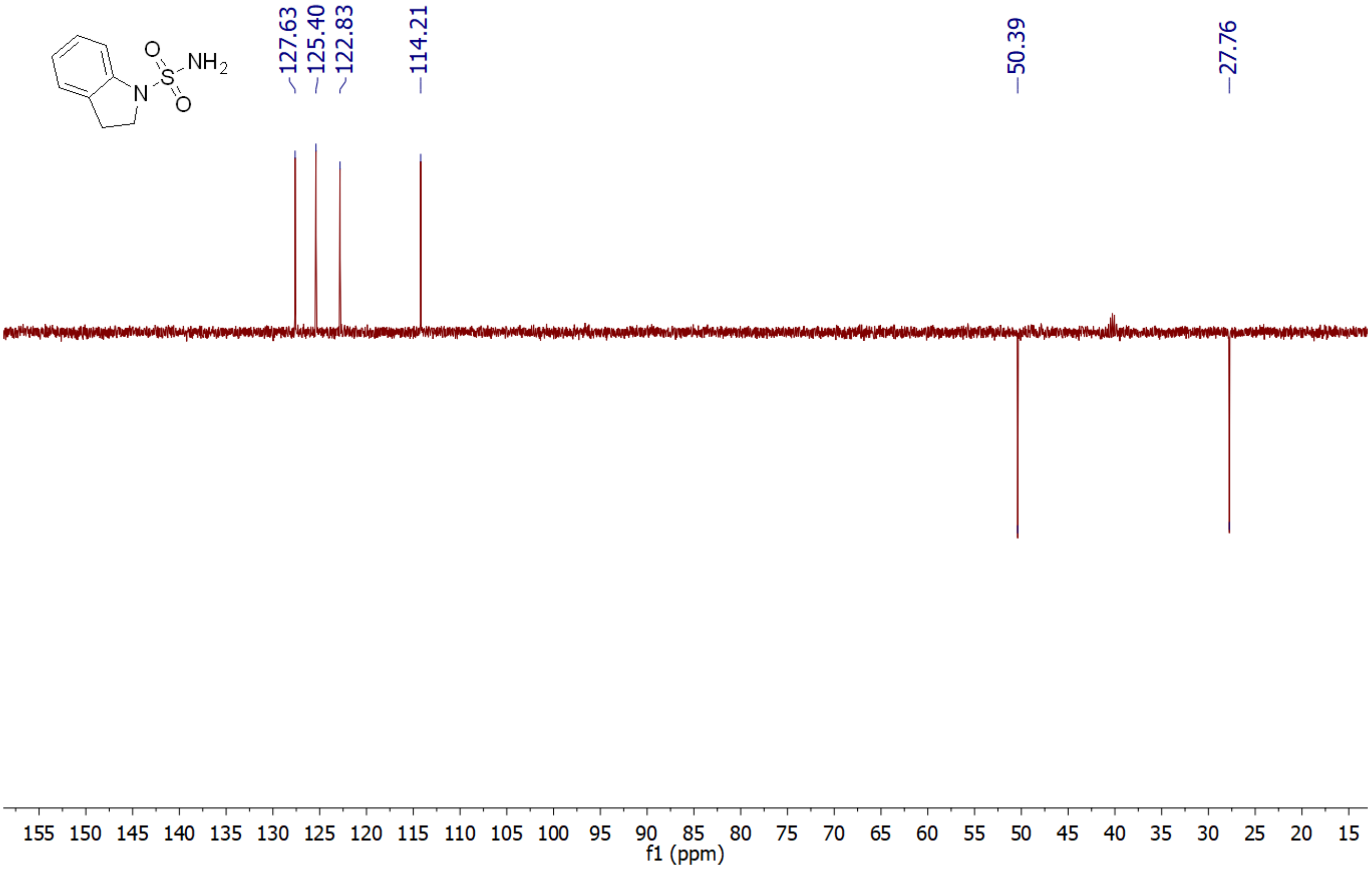
NH2
N
S
O
O
127.63
125.40
122.83
114.21
50.39
27.76
155 150 145 140 135 130 125 120 115 110 105 100 95 90 85 80 75 70 65 60 55 50 45 40 35 30 25 20 15
f1 (ppm)

^{1}H NMR,^{13}C (DEPT) and ^{19}F spectra of compound **2w**

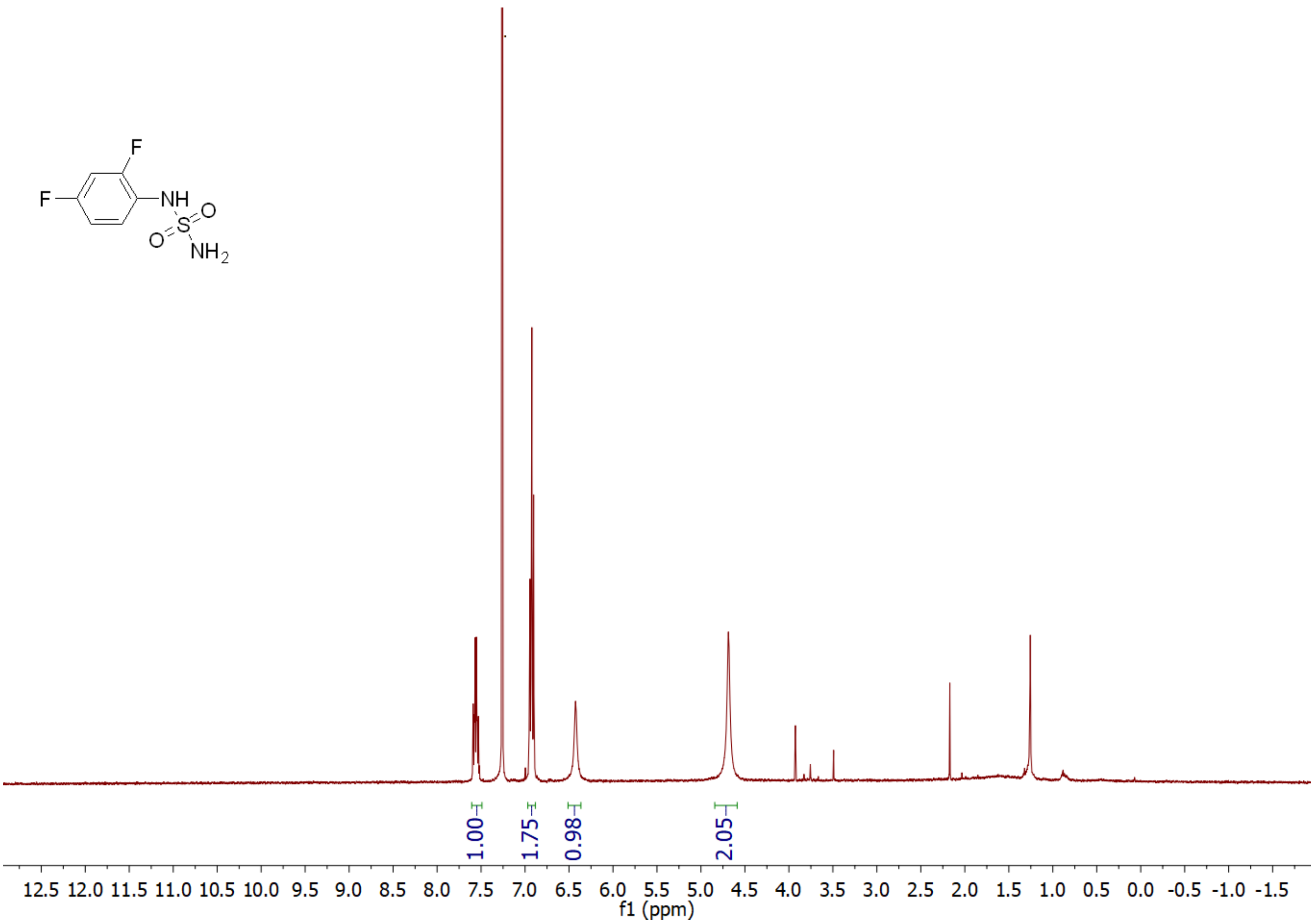

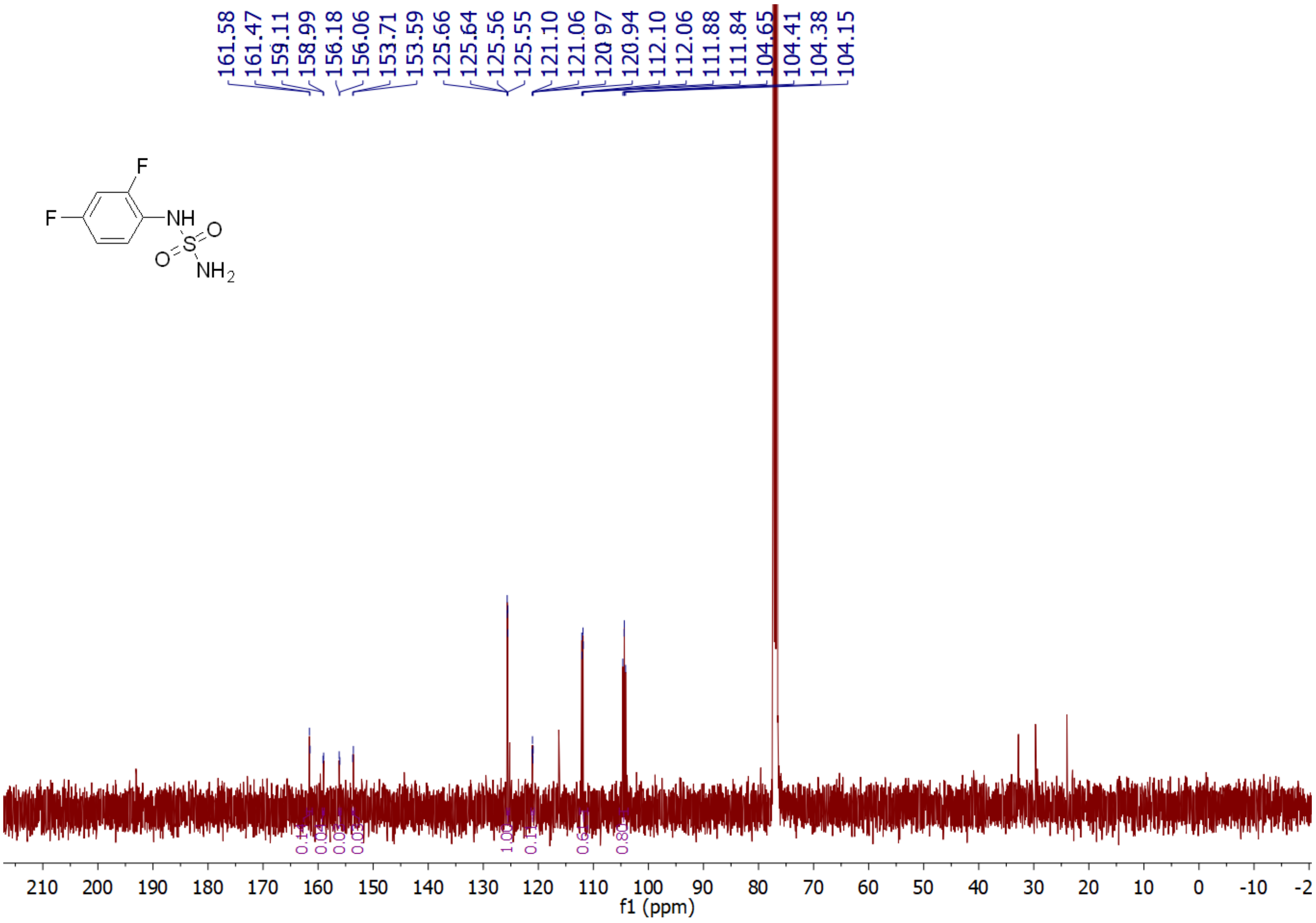

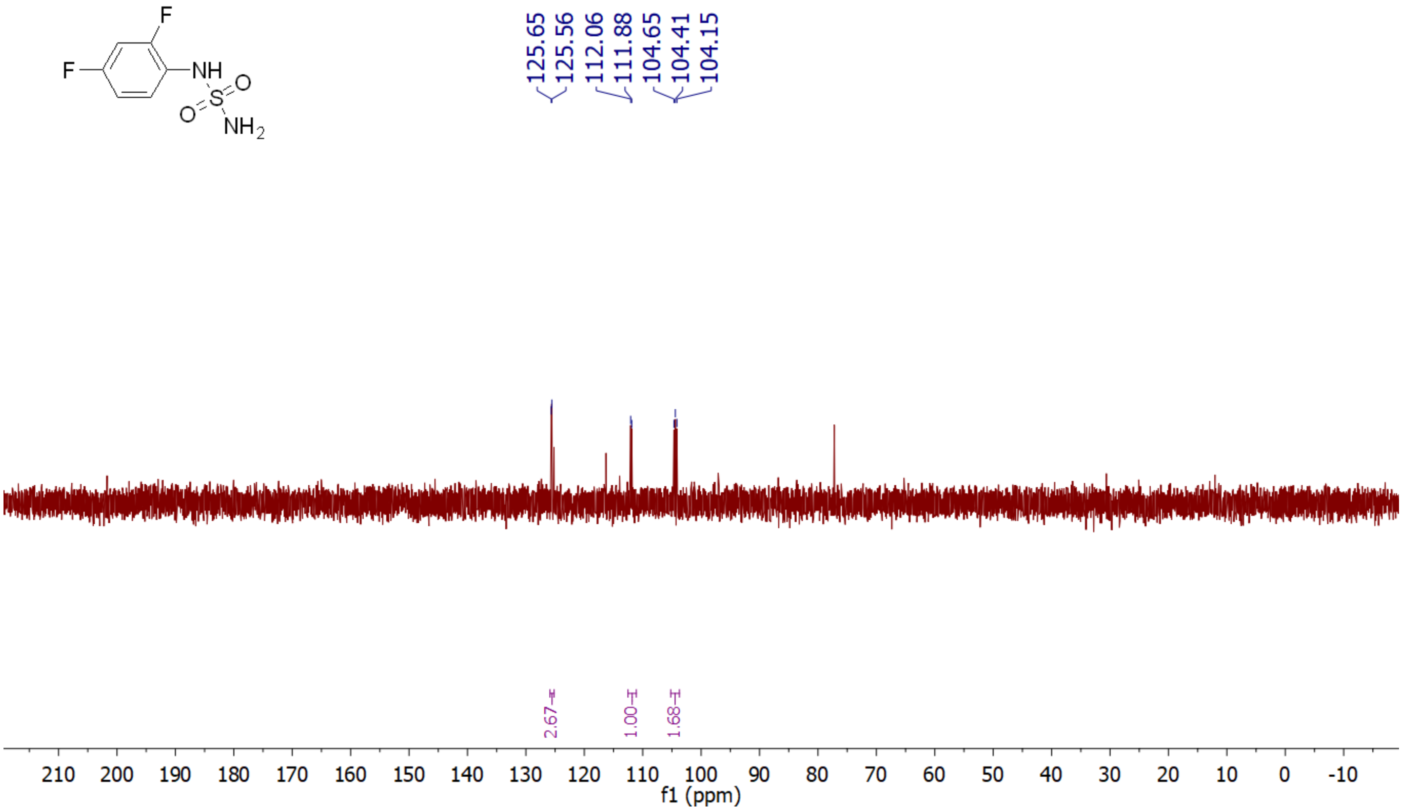
F
F
NH
O
S
O
NH2
125.65
125.56
112.06
111.88
104.65
104.41
104.15
2.67
1.00
1.68
210 200 190 180 170 160 150 140 130 120 110 100 90 80 70 60 50 40 30 20 10 0 -10
f1 (ppm)

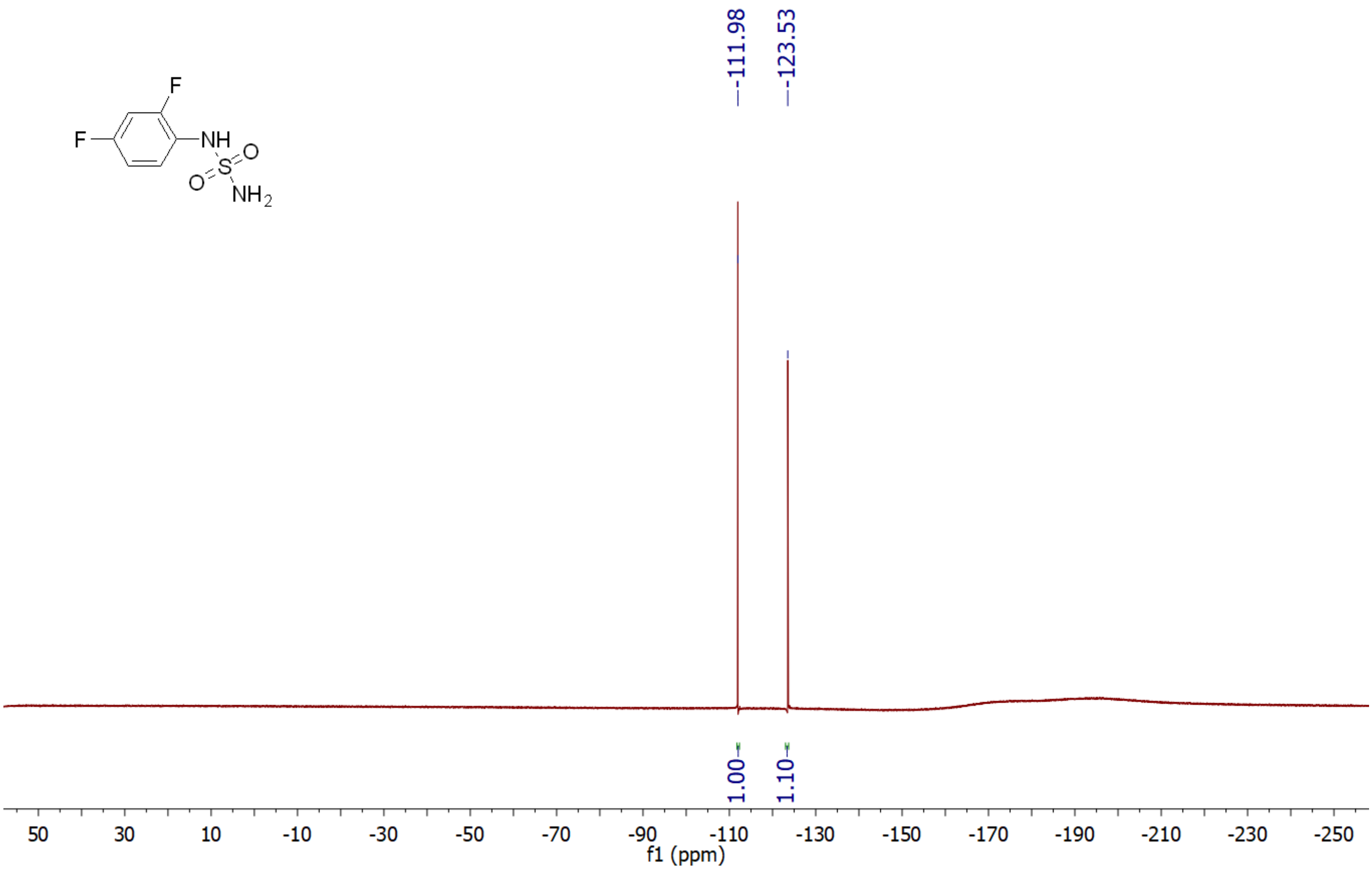
F
F
NH
O
S
O
NH2
-111.98
-123.53
1.00
1.10
50 30 10 -10 -30 -50 -70 -90 -110 -130 -150 -170 -190 -210 -230 -250
f1 (ppm)
